# Mapping interindividual dynamics of innate immune response at single-cell resolution

**DOI:** 10.1038/s41588-023-01421-y

**Published:** 2023-06-12

**Authors:** Natsuhiko Kumasaka, Raghd Rostom, Ni Huang, Krzysztof Polanski, Kerstin B. Meyer, Sharad Patel, Rachel Boyd, Celine Gomez, Sam N. Barnett, Nikolaos I. Panousis, Jeremy Schwartzentruber, Maya Ghoussaini, Paul A. Lyons, Fernando J. Calero-Nieto, Berthold Göttgens, Josephine L. Barnes, Kaylee B. Worlock, Masahiro Yoshida, Marko Z. Nikolić, Emily Stephenson, Gary Reynolds, Muzlifah Haniffa, John C. Marioni, Oliver Stegle, Tzachi Hagai, Sarah A. Teichmann

**Affiliations:** 1 Wellcome Sanger Institute, Wellcome Genome Campus, Cambridge, UK; 2 Medical Support Center of Japan Environment and Children’s Study (JECS), National Center for Child Health and Development, Tokyo, Japan; 3 European Molecular Biology Laboratory, European Bioinformatics Institute, Wellcome Genome Campus, Hinxton, UK; 4 Open Targets, Wellcome Genome Campus, Hinxton, UK; 5 Cambridge Institute of Therapeutic Immunology and Infectious Disease, Jeffrey Cheah Biomedical Centre, Cambridge, UK; 6 Department of Medicine, University of Cambridge, Cambridge, UK; 7 Wellcome-MRC Cambridge Stem Cell Institute, University of Cambridge, Cambridge, UK; 8 UCL Respiratory, Division of Medicine, University College London, London, UK; 9 University College London Hospitals NHS Foundation Trust, London, UK; 10 Biosciences Institute, Newcastle University, Newcastle upon Tyne, UK; 11 NIHR Newcastle Biomedical Research Centre, Newcastle Hospitals NHS Foundation Trust, Newcastle upon Tyne, UK; 12 Department of Dermatology, Newcastle Hospitals NHS Foundation Trust, Newcastle upon Tyne, UK; 13 Cancer Research UK Cambridge Institute, University of Cambridge, Cambridge, UK; 14 Division of Computational Genomics and Systems Genetics, German Cancer Research Center, Heidelberg, Germany; 15 European Molecular Biology Laboratory, Genome Biology Unit, Heidelberg, Germany; 16 Shmunis School of Biomedicine and Cancer Research, George S. Wise Faculty of Life Sciences, Tel Aviv University, Tel Aviv, Israel; 17 Theory of Condensed Matter Group, Cavendish Laboratory/Department of Physics, University of Cambridge, Cambridge, UK

**Keywords:** Transcriptomics, RNA sequencing, Software, Autoimmune diseases, Infectious diseases

## Abstract

Common genetic variants across individuals modulate the cellular response to pathogens and are implicated in diverse immune pathologies, yet how they dynamically alter the response upon infection is not well understood. Here, we triggered antiviral responses in human fibroblasts from 68 healthy donors, and profiled tens of thousands of cells using single-cell RNA-sequencing. We developed GASPACHO (GAuSsian Processes for Association mapping leveraging Cell HeterOgeneity), a statistical approach designed to identify nonlinear dynamic genetic effects across transcriptional trajectories of cells. This approach identified 1,275 expression quantitative trait loci (local false discovery rate 10%) that manifested during the responses, many of which were colocalized with susceptibility loci identified by genome-wide association studies of infectious and autoimmune diseases, including the *OAS1* splicing quantitative trait locus in a COVID-19 susceptibility locus. In summary, our analytical approach provides a unique framework for delineation of the genetic variants that shape a wide spectrum of transcriptional responses at single-cell resolution.

## Abstract (web summary)

GASPACHO is a statistical method that identifies nonlinear dynamic genetic effects using single-cell RNA-seq data. Analysis of an antiviral response in human fibroblasts identifies 1,275 expression QTLs, many of which colocalize with risk loci for autoimmune and infectious diseases.

## Main

The innate immune response is a cell-autonomous program that induces an antiviral state in infected and nearby cells and alerts the immune system of the invading pathogen^1^. Dysregulation of this response can affect a wide range of inflammatory and autoimmune diseases and determine the outcome of infection^2–6^. Common genetic variants have been shown to modulate transcriptional responses to various viral and bacterial stimuli, and to contribute to disease onset and progression^7–11^. Most past gene-expression-focused studies of this program are based on bulk RNA-sequencing (RNA-seq) technologies, which do not fully elucidate the continuous dynamics of transcriptional changes during the innate immune response. Single-cell genomic technologies are powerful approaches to study cell heterogeneity and transcriptional variability across cells^12^. Furthermore, by utilizing single-cell RNA-seq (scRNA-seq) profiling of tissues composed of several cell lineages, previous studies have successfully performed genetic association mapping of cell-type-specific expression^13–19^.

We here use full-length scRNA-seq of dermal fibroblasts from different human individuals, challenged with immune stimuli. Based on the pseudo-temporal reconstruction of these data, we map the transcriptional variation of the innate immune response at single-cell resolution. This provides the foundation for superimposing human genetic variation onto the transcriptional dynamics of this response. To this end, we develop a statistical approach based on a Gaussian process (GP) latent variable model (GPLVM)^20,21^ called GASPACHO (GAuSsian Processes for Association mapping leveraging Cell HeterOgeneity). This allows us to identify expression quantitative trait loci (eQTLs) that manifest at different stages of the response to stimuli.

We find more than a thousand eQTLs, hundreds of which are colocalized with known risk loci of diverse autoimmune and infectious diseases. We perform fine-mapping of the *OAS*1 locus, associated with COVID-19, to reveal the imbalanced expression of *OAS1* and *OAS3* genes during the antiviral innate immune response. We further integrate these data with eQTLs from a COVID-19 patient cohort dataset of peripheral blood mononuclear cell (PBMC) scRNA-seq^22^, as well as with scRNA-seq data of infected nasal epithelial cells from 33 patients with COVID-19 (ref. ^23^).

Overall, our study illustrates how coupling single-cell transcriptomics with a cutting-edge statistical approach can identify dynamic effects of human trait-associated genetic variants in different contexts of activation of antiviral innate immunity and, in general, in diverse cellular dynamic processes.

## Results

### Cell stimulation to study antiviral responses in fibroblasts

To study the innate immune expression program that is triggered upon viral infection, we exposed primary dermal fibroblasts from 68 donors from the Human Induced Pluripotent Stem Cell Initiative (HipSci)^24^ to two stimulants: (1) Poly(I:C), a synthetic double-stranded RNA (dsRNA) that is rapidly recognized by viral sensors and elicits primary antiviral and inflammatory responses; and (2) interferon-β (IFN-β), a cytokine that upregulates a secondary wave of response in both infected and bystander cells, and shifts the cells into an antiviral mode, where hundreds of interferon-stimulated genes (ISGs) are upregulated to contain the infection.

**Fig. 1:**
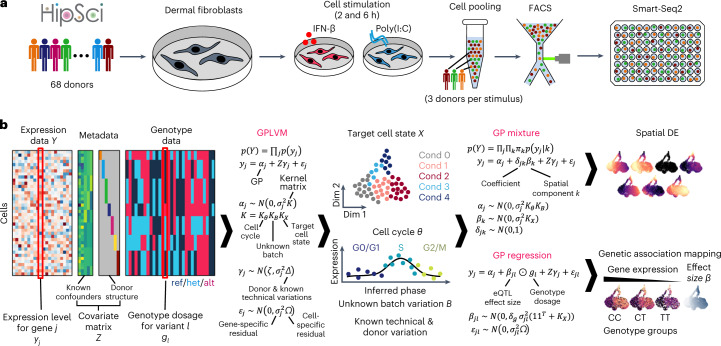
Schematics of experiment and statistical analysis. **a**, Experimental design using the in vitro fibroblast system. **b**, GASPACHO framework. Expression data and relevant metadata (known confounding factors) as well as donor (cell line) structure are used to construct a GPLVM to extract the target cell state, while dissecting cell cycle effect and other known and unknown technical variability including donor–donor variation. The result of the GPLVM is then utilized for the subsequent analyses of spatial DE analysis using a GP mixture model and the genetic association mapping using a GP regression model (Methods). Cond, experimental condition; Dim, dimension; ref/het/alt, reference homozygote/heterozygote/alternative homozygote.

**Extended Data Fig. 1:**
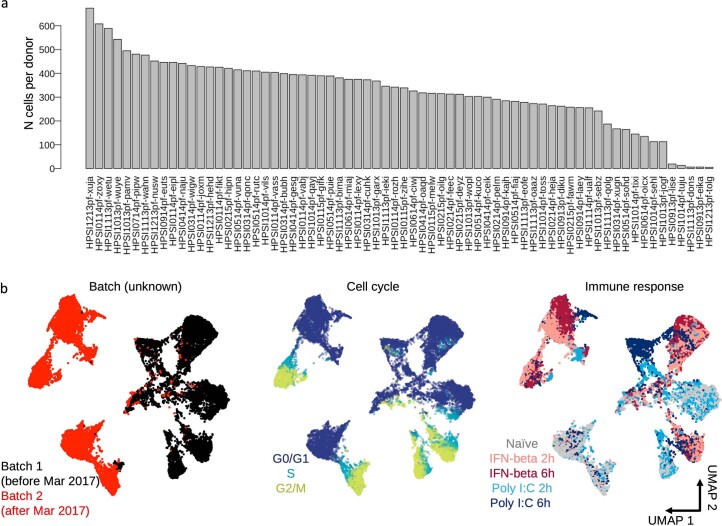
Data and quality controls. **a**. Barplot shows the number of cells for each donor (cell line). **b**. UMAPs calculated from the first 10 principal components from the data. Points are coloured by unknown batch effect (well correlated with experimental date), cell cycle phase estimated from known marker genes (Online Methods) and experimental conditions.

We collected cells exposed to each of the two stimuli after 2 and 6 h of stimulation (Fig. 1a). Following this, scRNA-seq profiling was performed using a plate-based full-length transcript approach (Methods). After quality control, 22,188 high-quality cells were obtained across 128 plates, with each plate containing cells from three donors (Fig. 1a). The donor identity for each cell was inferred from scRNA-seq read data using known genotypes made available by HipSci (Extended Data Fig. 1a and Methods). Preliminary analysis showed that our data display high cell-to-cell variability in gene expression both within and across donors, as observed in previous studies by us and others^25–27^. In fact, our data were confounded by various technical and biological factors, including library preparation in different batches, and cell cycle effects (Extended Data Fig. 1b). The complex nature of these data, along with their confounders, motivated us to develop an approach that reveals the genetic and physiologically relevant variation, while computationally masking confounding factors.

### Uncovering cell-state dynamics using GPs

Single-cell transcriptomics (as compared with bulk) enables us to uncover hidden states of complex biological processes, while also requiring regression of technical effects and biological variation that is not of interest (for example, proliferation). We developed GASPACHO, which utilizes a GPLVM to uncover the dynamic cell states of interest, while adjusting periodic cell cycle variation and both known and unknown technical variations (such as, donor and Smart-Seq2 plate variations) simultaneously (Fig. 1b and Methods). The use of a GPLVM allows us to capture smooth and continuous nonlinear trends in gene expression along the latent variables, for which other methods such as the standard linear principal component analysis will not work well.

Although there are other models that utilize a GPLVM to study single-cell dynamics^28,29^, the unique aspect of our GPLVM approach is that it explicitly takes account of the donor variation as well as other known confounding effects (such as technical batches) as additional random effect terms (Fig. 1b and Methods). These confounders are known to inflate the type I error in downstream analyses, such as in differential expression^30^ (DE), leading to false discovery of differentially expressed genes. As detailed below, the model output not only enabled us to look at the architecture of the antiviral response in the cell-state space, but also provided a rigorous statistical framework of (1) spatial DE analysis and (2) genetic association mapping using genotype data obtained from the donor of origin for each cell.

Specifically, the gene expression variation in the target cell-state space was inferred by a GP mixture model in which an additional GP component is introduced into the model to capture hidden spatial DE patterns^31^ of gene expression in the latent space (Fig. 1b and Methods). The genetic association mapping was also carried out by using a GP regression model in which the effect size of a quantitative trait locus (QTL) was modeled as a GP in the target cell-state space. Here, the additional GP was multiplied by the genotype dosage (the number of alternative alleles for each donor) to capture the gene–environment interaction^32^ (Fig. 1b and Methods). Importantly, the eQTL effect is obtained at single-cell resolution, and the model does not require aggregation of single-cell data into pseudo-bulk data, which is a common eQTL mapping strategy. Thus, we can study the effect of genetic variants without losing the continuum of transcriptional dynamics and its spectrum across individual cells. We performed a comprehensive analysis to assess our method in terms of both sensitivity and specificity with another single-cell-based eQTL mapping approach, CellRegMap^33^, using simulation-based datasets (section 3 of the Supplementary Notes). We have implemented the software in R, which is available from github (https://github.com/natsuhiko/GASPACHO).

### Primary and secondary responses of innate immunity

**Fig. 2:**
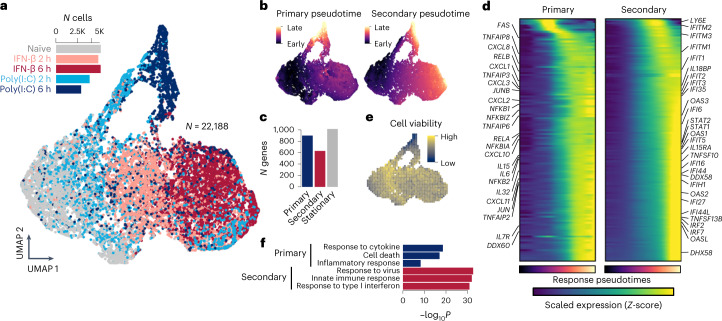
Innate immunity captured by GPLVM and GP mixture model. **a**, UMAP of latent variables capturing innate immune variation between cells. Cells are colored by the five experimental time points (gray, naïve state; pink, IFN-β 2 h; brown, IFN-β 6 h; blue, Poly(I:C) 2 h; navy, Poly(I:C) 6 h). **b**, Estimated pseudotime for primary and secondary responses using the GP mixture model. UMAP coordinates are identical to **a**. **c**, Barplot shows the numbers of response and stationary genes. **d**, Heatmaps show dynamic gene expression changes along primary or secondary response pseudotime. The pseudotime color scale corresponds to **b**. The expression color (navy to yellow) shows the magnitude of scaled expression for each gene (Z-score). **e**, UMAP shows a predicted Achilles cell viability using CEVIChE (Methods). UMAP coordinates are identical to **a**. **f**, Barplot shows the enrichment of gene ontology terms for primary and secondary genes. *P* values were computed using Fisher’s one-tailed test with Bonferroni multiple testing correction implemented in gprofiler2 on R. K, thousand.

**Extended Data Fig. 2:**
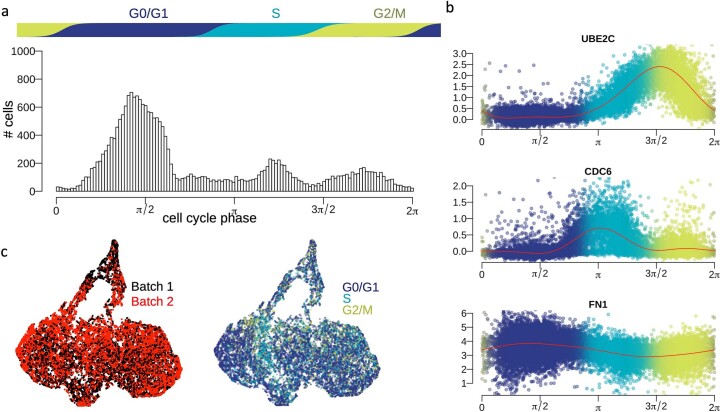
Observed cellular states in the fibroblast single cell data. **a**. Histogram shows the distribution of estimated cell cycle phase by GPLVM (Online Methods). **b**. Scatterplots show scaled expression of known cell cycle genes (UBE2C and CDC6) and a gene highly expressed in G0/G1 phase (FN1). The red curves show the posterior mean estimates of expression levels by GPLVM (Online Methods). **c**. UMAPs of the target cell states coloured by unknown batch or cell cycle phase. UMAP coordinates are identical to Fig. 2a.

We first applied the GPLVM to adjust for the cell cycle and unknown batch effects in our data (Extended Data Fig. 2a,b) and successfully extracted the innate immune state embedded in the data (Fig. 2a). We also confirmed that the extracted immune state was independent of cell cycle or the unknown batch variations (Extended Data Fig. 2c). We observed two major cell trajectories: one for response to IFN-β from the naïve state (*x* axis) and the other for response to Poly(I:C) (*y* axis).

**Extended Data Fig. 3:**
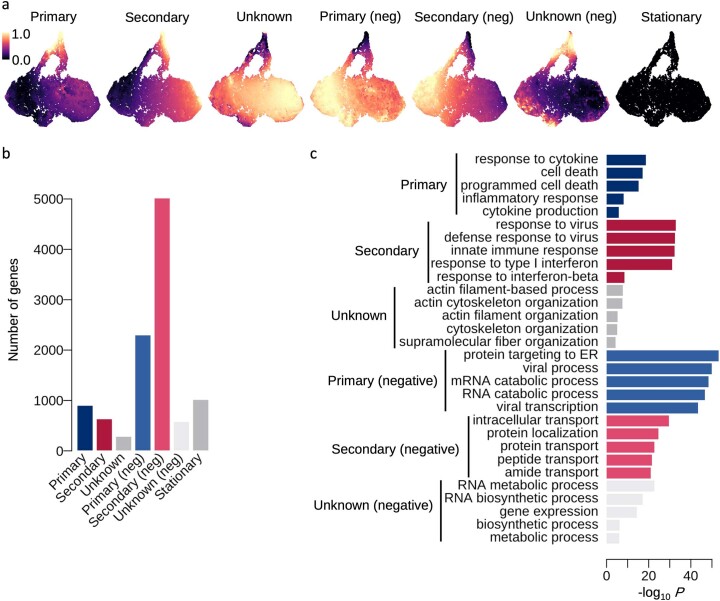
Spatial differential expression analysis to classify genes into different innate immune responses. **d**. UMAPs of the seven different mixture components estimated from the GP mixture model (Online Methods). The values are quantile-normalised as in [0,1], and also treated as pseudotime for the first two components (primary and secondary). UMAP coordinates are identical to Fig. 2a. **e**. The number of genes categorised in each of the seven components. **f**. Top GO terms enriched with the genes detected in each of the seven DE categories.

We then applied the GP mixture model which revealed two independent innate immune responses, the primary response by virus infection and the secondary response for bystander cells due to IFN-β secretion by the infected cells or direct IFN-β stimulation (Fig. 2b and Extended Data Fig. 3a). Those responses were highly overlapping on the Uniform Manifold Approximation and Projection (UMAP), suggesting those two processes are independently and simultaneously happening in each cell. In total, the GP mixture model discovered 903 and 636 genes upregulated during the primary and secondary responses, respectively (hereafter referred to as primary response genes and secondary response genes), while 1,020 genes were expressed uniformly across all cells in different experimental conditions (referred to as stationary genes) (Fig. 2c and Extended Data Fig. 3b). Many cytokine and chemokine genes were upregulated along the primary response, while ISGs were upregulated along the secondary response (Fig. 2d). Interestingly, the primary response was also correlated with the predicted cell viability by CEVIChE (CEll VIability Calculator from gene Expression) (Fig. 2e and Methods). The gene ontology enrichment analysis for the primary and secondary genes clearly demonstrated that primary response genes are enriched for cell death and inflammatory response, while secondary response genes are enriched for type I interferon response (Fig. 2f and Extended Data Fig. 3c).

### Dynamic genetic effect on innate immune response

**Extended Data Fig. 4:**
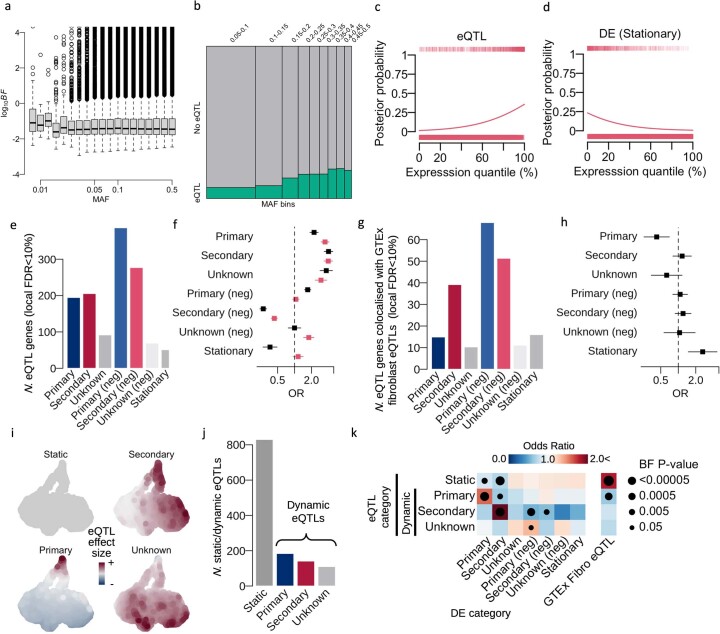
Characteristics of mapped response eQTLs. **a**. Distributions of Bayes factors from our fibroblast data for *N* = 1 million random gene-variant pairs. Here we used all variants with MAF > 0% for the Bayes factor calculation (not only variants with MAF > 5%). Here The bottom and top of each box (Q1 and Q3) are the 25th and 75th percentile (the lower and upper quartiles, respectively), and the band near the middle of the box is the 50th percentile (the median). The ends of the whiskers are defined as follows: upper whisker = min(max value, Q3 + 1.5*IQR) and lower whisker = max(min value, Q1–1.5*IQR), where IQR = Q3-Q1 is the box length. **b**. Mosaic plot shows the number of eQTLs (local FDR = 10%) in different MAF bins (column). We included the number of non-eQTL (the top row of the plot coloured by gray) in comparison to the number of eQTLs (bottom row coloured by green). **c-d**. The trend of posterior probabilities of eQTLs or stationary genes (one of the 7 differential expression categories) against gene expression levels. The line was estimated using logistic regression where the response variable is the posterior probability greater than 0.9 against the expression quantile based on the average expression across all cells for each gene. **e**. The number of eQTL genes stratified by the spatial DE genes demonstrated in Extended Data Fig. 2d. **f**. Forest plot showing the enrichment of the 1,275 eQTL genes in each of the 7 DE categories. The black dots show non-adjusted eQTL enrichment, and the red dots show the enrichment following adjustment for gene expression levels (Online Methods). The error bars in the forest plot show 95% confidence intervals (standard errors) of odds ratios using *N* = 10,748 genes as independent samples (see Online Methods for details). **g**. The number of eQTL genes colocalised with GTEx fibroblast eQTLs in the 7 different DE gene categories. **h**. Forest plot shows the enrichment of the eQTL colocalised with GTEx fibroblast eQTLs in the 7 different DE gene categories. The error bars in the forest plot show 95% confidence intervals (standard errors) of odds ratios using *N* = 10,748 genes as independent samples (see Online Methods for details). **i**. UMAPs showing spatial distributions of eQTL effect sizes estimated from the GP mixture model with 4 different spatial eQTL categories (static, primary, secondary and unknown; Online Methods). UMAP coordinates are identical to Fig. 2a. **j**. The numbers of static and dynamic eQTLs estimated from the GP mixture model. **k**. Heatmap showing the enrichment of spatial eQTL categories for the 7 DE categories and the GTEx fibroblast eQTLs. Colour scale shows the odds ratio of enrichment and the size of dots denotes the magnitude of Bonferroni-corrected *P*-values of the enrichment. The odds ratios and corresponding *P*-values (from a one-sided Chi-square test) were computed using *N* = 10,748 genes as independent samples (see Online Methods for details). Dots with *P* > 0.05 were omitted.

We then mapped eQTLs along innate immune responses using the GP regression model to assess genetic association in single-cell resolution (Methods). We discovered 1,275 eQTL genes (local false discovery rate (FDR) 10%) among 10,748 genes expressed, at least, in 10% of total cells. We mapped eQTLs in the HLA region where 91 genes were tested, and 25 genes were eQTLs (local FDR 10%). Because the number of eQTLs was high for the modest sample size, we examined whether there was an inflation of test statistics for eQTL variants with lower minor allele frequencies (MAFs) and confirmed that there was no inflation in the Bayes factors nor in the number of discovered eQTLs in lower MAF bins (Extended Data Fig. 4a,b). We note that the eQTL genes discovered are strongly enriched in highly expressed genes (Extended Data Fig. 4c), but this is not the case for differentially expressed genes (for example, stationary genes were depleted in highly expressed genes; Extended Data Fig. 4d).

**Fig. 3:**
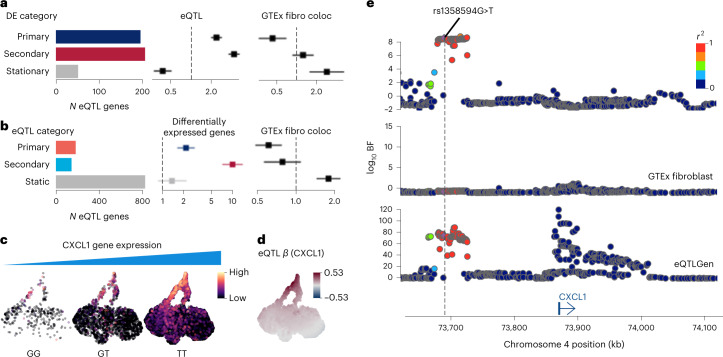
Characteristics of response eQTLs mapped using GP regression. **a**, Barplot showing the numbers of eQTLs (local FDR < 10%) that are primary and secondary response genes or stationary genes. Forest plots showing the enrichment of discovered eQTLs for the DE categories and GTEx fibroblast eQTL colocalizations. The error bars in the forest plots show 95% confidence intervals (standard errors) of odds ratios using *N* = 10,748 genes as independent samples (Methods). **b**, Barplot showing the numbers of static and dynamic eQTLs (primary and secondary response eQTLs). Forest plots showing the enrichment of response eQTLs for differentially expressed genes (primary, secondary response and static eQTLs for primary and secondary response genes, and for stationary genes, respectively) and the enrichment of response eQTLs of GTEx fibroblast eQTL colocalization. The error bars in the forest plots show 95% confidence intervals (standard errors) of odds ratios using *N* = 10,748 genes as independent samples (Methods). **c**, UMAPs showing *CXCL1* expression levels stratified by different genotype groups at rs1358594G>T. UMAP coordinates are identical to Fig. 2a. **d**, UMAP shows the distribution of eQTL effect size (*β*) at rs1358594. The alternative allele (T) is assessed. UMAP coordinates are identical to Fig. 2a. **e**, Locus zoom plot of *CXCL1* eQTL association Bayes factors around the *CXCL1* gene (top, in-house data; middle, GTEx fibroblast eQTL; bottom, eQTLGen blood eQTL). BF, Bayes factor; fibro coloc, colocalisation with GTEx fibroblast eQTLs.

We found that 15% and 16% of our eQTL genes are primary and secondary response genes, respectively (Fig. 3a and Extended Data Fig. 4e). These genes are strongly enriched within the discovered eQTLs (Fig. 3b and Extended Data Fig. 4f). Because eQTL genes tend to be highly expressed (Extended Data Fig. 4c), we adjusted enrichments for the average expression level (Methods) and confirmed that the result is broadly the same (Extended Data Fig. 4f). We also found that primary response genes are depleted in colocalization between our eQTLs and fibroblast eQTLs from the Genotype-Tissue Expression (GTEx) Project, while the stationary genes are enriched (odds ratio of 2.8) (Fig. 3a, Extended Data Fig. 4g,h and Methods), suggesting our eQTLs are highly context-specific. We note that the odds ratio observed suggests a notable agreement between the two datasets (GTEx and our data), which is meaningful given the experimental and technical differences between the two studies which can strongly affect gene expression and subsequent eQTL discovery.

Next, we classified our eQTLs into static and dynamic eQTLs. We then further classified the dynamic eQTLs into distinct spatial patterns of their effect sizes utilizing a similar GP mixture model that segregated response genes (Methods). We discovered 830 (65%) (posterior probability > 0.9) of our eQTLs were static eQTLs whose genetic effect is ubiquitous in any cellular state (Fig. 3b and Extended Data Fig. 4I,j). Furthermore, 184 (14%) and 141 (11%) of our eQTLs were primary and secondary response eQTLs, respectively, whose patterns resembled the spatial DE patterns of primary and secondary response genes (Extended Data Fig. 4i). We also found that the primary/secondary response eQTLs were enriched with primary/secondary response genes, respectively, while the static eQTLs were enriched with other types of differentially expressed genes (Fig. 3b and Extended Data Fig. 4k). The fact that the static eQTLs are significantly enriched with GTEx fibroblast eQTLs, while the primary response eQTLs are depleted, further supports the notion that our eQTLs are specific to the innate immune response and not detectable in a naïve cellular state. We note here that, as was shown in our simulation study (section 3 of the Supplementary Notes), the GP mixture model is robust against the lower detection limit of gene expression quantified by the sequencing technology. Therefore, the misclassification rate in the real data analysis is likely to be minimal.

As an example, the *CXCL1* gene is a known primary innate immune response gene and a primary response eQTL (as expected from its known functions as an important chemokine in this response). It is mostly expressed in later time points of Poly(I:C)-stimulated cells (Fig. 3c), and its expression level is higher for the alternative allele T at rs1358594 compared with the reference allele G (Fig. 3d). This eQTL signal was discovered more than 100 kilobases (kb) downstream of the gene’s transcription start site (TSS) and only detected upon cell stimulation by Poly(I:C), but not in the naïve condition, as also shown in the GTEx naïve fibroblast eQTL data (Fig. 3e), though the gene is highly expressed in the GTEx data (median transcripts per million (TPM) = 111). We note, however, that this eQTL was discovered in eQTLGen data with tens of thousands of blood samples (Fig. 3e). This might suggest that the eQTL signal is present in unstimulated conditions in immune cells (such as in blood).

**Extended Data Fig. 5:**
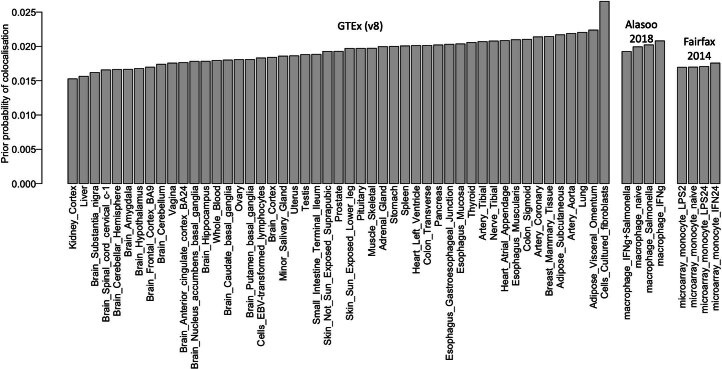
eQTL sharing with GTEx and other immune cell eQTLs. Prior probability inferred by the pairwise hierarchical model (Online Methods) fitted between our eQTLs and those for the 48 GTEx tissues as well as immune cells (monocytes and iPSC-derived macrophages) under different stimulation conditions.

To compare our eQTLs with other datasets, we repeated the colocalization analysis between our stimulated fibroblast eQTLs and all primary GTEx tissues^34^ as well as with immune cells (monocytes and induced pluiripotent stem cell (iPSC)-derived macrophages) stimulated using different conditions based on previous studies^7,9^. We found that the naïve GTEx fibroblast showed the highest prior probability of colocalization (Extended Data Fig. 5 and Methods).

### Fine-mapping eQTLs with epigenetic data

**Fig. 4:**
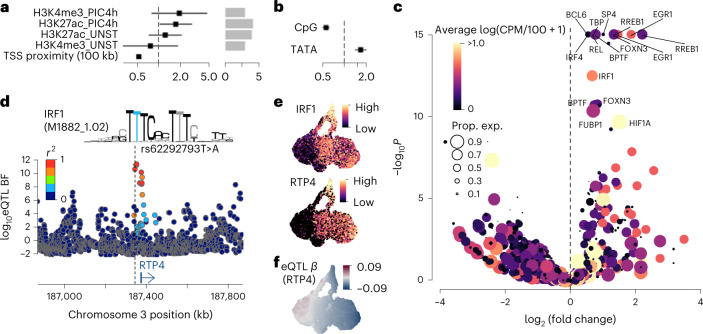
Fine-mapping eQTLs with epigenetic data. **a**, Enrichment eQTLs for regulatory regions characterized by ChIP–seq for the two histone modifications (H3K27ac and H3K4me3) under two different conditions (UNST, unstimulated; PIC4h, Poly (I:C) 4-h stimulation). The error bars in the forest plot show 95% confidence intervals (standard errors) of odds ratios using *N* = 10,748 genes as independent samples (Methods). **b**, eQTL enrichment for genes with TATA-box or CGI between TSS and 100 bp upstream. The error bars in the forest plots show 95% confidence intervals (standard errors) of odds ratios using *N* = 10,748 genes as independent samples (Methods). **c**, Enrichment of lead eQTL variants for various TF motifs. The color of each point shows the average expression across all cells and the point size shows the frequency of cells with CPM > 0 for each TF gene in our fibroblast data. **d**, Locus zoom plot showing the *RTP4* eQTL association Bayes factors. The lead eQTL variant rs62292793T>A disrupts the putative IRF1 binding motif (M1882_1.02; CIS-BP v.1.02) upstream of the *RTP4* gene. **e**, UMAPs showing expression levels of *IRF1* and *RTP4*. UMAP coordinates are identical to Fig. 2a. **f**, UMAP showing eQTL effect size of *RTP4*. UMAP coordinates are identical to Fig. 2a. CPM, counts per million; Prop. exp., proportion of cells the gene expressed.

We have also performed fine-mapping using epigenetic data (histone modification chromatin immunoprecipitation followed by sequencing (ChIP–seq) of active promoters and enhancers) originating in dsRNA-stimulation of human dermal fibroblasts^27^ (Methods). We identified that more than 10% of the putative causal eQTL variants discovered by the hierarchical model (Methods) are located in the *cis*-regulatory regions characterized by the ChIP–seq data (Fig. 4a). Those variants are especially enriched for promoter peaks characterized by H3K4me3 antibody in Poly(I:C)-stimulated cells (Fig. 4a). Importantly, our eQTLs were also strongly enriched around the TSS, and the number of eQTLs was reduced by 46% every 100 kb further away from the TSS (Fig. 4a).

We next tested whether promoter architecture affects the variability between individuals. It was previously shown by us and others^27,35^ that genes containing TATA boxes in their promoters tend to vary more in transcription between species and conditions and between individual cells responding to an immune stimulus, whereas promoters containing CpG islands (CGIs) tend to vary less and be transcriptionally more homogenous. We observe that genes with TATA-containing promoters are 1.4-times more highly enriched with eQTLs in comparison with genes with CGI-containing promoters (Fig. 4b).

Lastly, using the eQTL variants fine-mapped based on ChIP–seq annotations, we examined which transcription factor (TF) motifs were disrupted by the lead eQTL variants (Methods). We found interferon regulatory factors 1 and 4 (IRF1 and IRF4) as well as REL and ATF4 were significantly enriched (Fig. 4c). An example of putative TF binding disruption was discovered in *RTP4* eQTL (Fig. 4d), where the alternative allele of a promoter-flanking eQTL variant (rs62292793T>A) may disrupt an IRF1 motif that significantly reduces putative TF binding affinity, which subsequently downregulates the *RTP4* expression (Fig. 4e,f). Furthermore, the TATA motif (TBP) is also found to be disrupted by eQTL variants (Fig. 4c), further suggesting the importance of TATA regulation in modulating the response and its variability among individuals, as previously suggested in the mammalian immune response^27,35^ and in other systems^36^.

### Colocalization with autoimmune and infectious disease genome-wide association studies

**Fig. 5:**
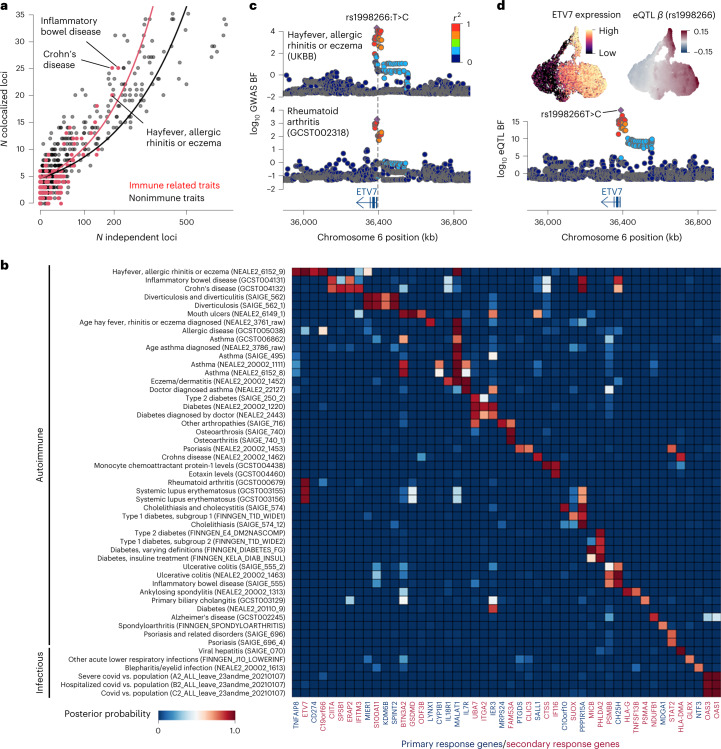
eQTL and disease GWAS colocalization. **a**, Scatter plot shows the number of colocalized eQTLs with posterior probability greater than 0.5 (*y* axis) against the number of independent loci where GWAS and eQTL do not share the putative causal variant (Methods). **b**, Heatmap shows the posterior probability of colocalization between eQTLs and GWAS loci. Only primary (colored by navy) and secondary (colored by red) response genes were shown. **c**, Locus zoom plots show the association of rheumatoid arthritis with hay fever, allergic rhinitis or eczema around the *ETV7* gene. Points are colored by the linkage disequilibrium index (*r*^2^) with the GWAS index variant rs1008266T>C. **d**, UMAPs show the scaled *ETV7* expression and the eQTL effect size (*β*) at the lead eQTL variant rs1998266T>C. Locus zoom plot shows the eQTL association for *ETV7*. UMAP coordinates are identical to Fig. 2a. UKBB, UK BioBank.

One of the purposes of eQTL mapping is to uncover the target genes and related cell states at each genetic locus implicated by genome-wide association studies (GWASs) of common complex traits. Here, we tested colocalization of our eQTLs with risk loci from 701 GWASs (each with five or more genome-wide significant loci), of which 112 were broadly immune-related, including autoimmune and chronic inflammatory diseases such as Crohn’s disease and infectious diseases such as COVID-19 (Methods). We discovered 7,934 unique gene–trait combinations with the posterior probability of a single shared causal variant between an eQTL and a GWAS locus greater than 0.5. The combinations consisted of 643 different GWAS traits and 988 unique genes. We observed an excess of colocalized eQTLs for immune-related traits over nonimmune traits (Fig. 5a, *P* = 2.8 × 10^−6^, and Methods), likely reflecting the known involvement of innate immunity in each of the disease pathologies.

**Extended Data Fig. 6:**
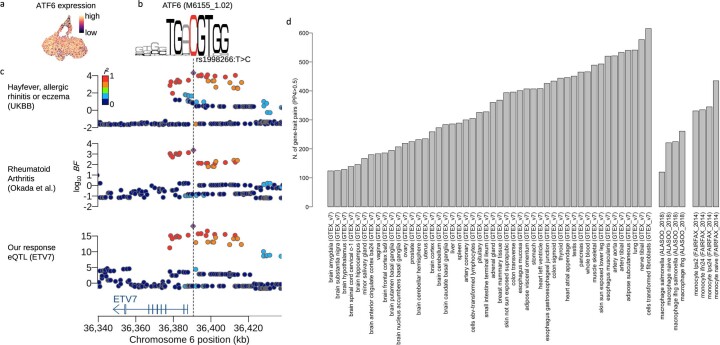
eQTL and disease GWAS colocalisation. **a**. ATF6 expression on the UMAP of fibroblast data. UMAP coordinates are identical to Fig. 2a. **b**. ATF motif (M6155_1.02; CIS-BP version 1.02). The nucleotide C coloured by red indicates the location of the eQTL variant rs1998266T>C. **c**. Locus zoom plots of hayfever, allergic rhinitis or eczema, rheumatoid arthritis and the ETV7 eQTL. **d**. The numbers of colocalised loci between our eQTLs and OpenTarges GWAS traits that were overlapping with the OpenTarges colocalisation resource for GTEx eQTLs (48 tissues) as well as monocytes and iPSC derived macrophages under various stimulation conditions.

We discovered 48 primary and secondary response genes that were specifically colocalized with 51 autoimmune and infectious disease loci, some of which were colocalized with multiple traits (Fig. 5b). For example, we detected an eQTL for the *ETV7* gene, which encodes a TF in the ETS family and plays a key role in hematopoiesis^37^. The eQTL was colocalized with rheumatoid arthritis (posterior probability = 0.98) and hay fever, allergic rhinitis or eczema (posterior probability = 0.99) (Fig. 5c). The gene is an ISG and the expression is upregulated during secondary response (Fig. 5d). The lead eQTL variant (rs1998266T>C) is shared with the GWAS traits, whose alternative allele C upregulates gene expression in stimulated conditions and also increases the risks of those GWAS traits (Fig. 5d). The alternative allele C also modifies the binding motif of the TF ATF6 putatively bound at the promoter region of *ETV7*, thereby potentially increasing the expression level (Extended Data Fig. 6a–c).

Lastly, we compared our colocalization results with those provided by Open Targets for 2,331 GWAS traits and 16 different eQTL studies (Data availability). We discovered that 6,327 gene–trait pairs were unique and only found in our colocalization results (posterior probability of colocalization > 0.5), whereas 1,607 gene–trait pairs are shared in both datasets (posterior probability of colocalization > 0.5), of which 615 also overlap with the GTEx fibroblast colocalization analysis (Extended Data Fig. 6d).

### Fine-mapping *OAS1* eQTL associated with SARS-CoV-2 infection

**Fig. 6:**
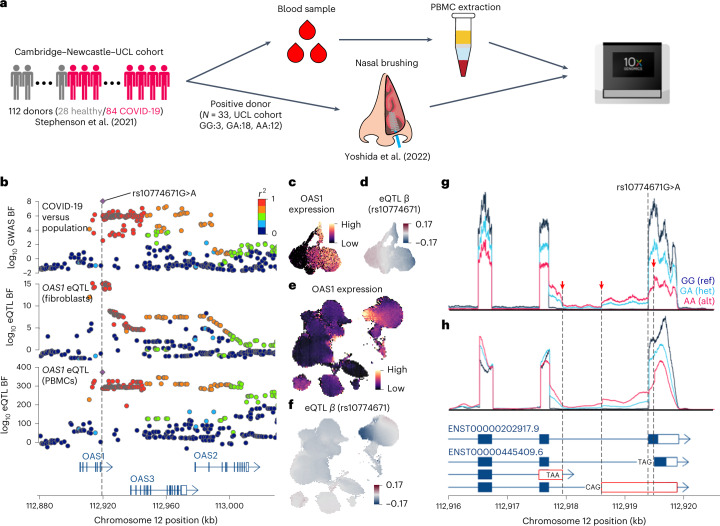
Fine-mapping *OAS1* locus using three different model systems. **a**, Schematic of in vivo system, COVID-19 study of PBMCs and nasal brushings to confirm the splicing QTL association in the *OAS1* gene. **b**, Locus zoom plots show the COVID-19 GWAS (COVID-19 versus population) association Bayes factors as well as eQTL associations of the *OAS1* gene in fibroblasts and PBMCs. **c**, UMAP shows the expression levels of the *OAS1* gene in fibroblasts. UMAP coordinates are identical to Fig. 2a. **d**, UMAP shows the eQTL effect size of the *OAS1* gene at rs10774671G>A. UMAP coordinates are identical to Fig. 2a. **e**, UMAP shows *OAS1* expression level in PBMCs. UMAP coordinates are identical to Extended Data Fig. 7a. **f**, UMAP shows the eQTL effect size of the *OAS1* gene at rs10774671G>A in PBMCs. UMAP coordinates are identical to Extended Data Fig. 7a. **g**, Sequencing coverage depth around the splicing variant rs10774671G>A, which creates three different isoforms, two of which are not annotated in Ensembl 90. scRNA-seq reads in fibroblasts were aggregated and stratified by the three different genotype groups (GG, reference homozygote; GA, heterozygote; AA, alternative homozygote). **h**, The 10x RNA-seq coverage depth of epithelial cells around *OAS1* 3′ end in nasal brushing samples taken from 33 COVID-19-positive adult patients was stratified and aggregated by the three genotype groups of rs10774671G>A as demonstrated in **g**. UCL, University College London.

In conjunction with the fibroblast system, we used two additional in vivo systems (Fig. 6a) to further fine-map the 12q24.13 (*OAS1*) locus that was reported in a GWAS of SARS-CoV-2 positive-infected individuals against population controls^38^ (index SNP: rs10774671G>A). The locus is colocalized with the *OAS1* eQTL in fibroblasts with a posterior probability of 1.0 (Figs. 5b and 6b). *OAS1* is a secondary response gene and is highly expressed upon IFN-β (at 2 h and later) and Poly(I:C) stimulation (at 6 h) (Fig. 6c). The alternative allele A of rs10774671 downregulates the expression level (Fig. 6d).

**Extended Data Fig. 7:**
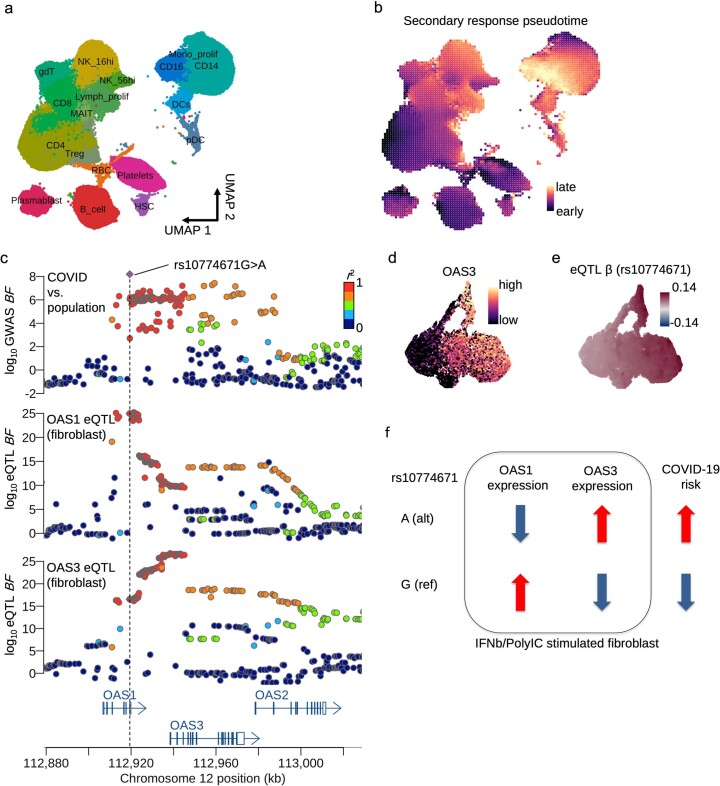
Fine-mapping OAS locus. **a**. UMAP shows the 18 different cell types annotated previously in the PBMC data^22^. **b**. UMAP shows the secondary response pseudotime calculated from the secondary response genes discovered in the fibroblast data. **c**. Locus zoom plot shows the COVID-19 GWAS, OAS1 and OAS3 eQTL (both in fibroblasts) associations. UMAP coordinates are identical to Extended Data Fig. 7a. **d**. UMAP shows the OAS3 gene expression. UMAP coordinates are identical to Fig. 2a. **e**. UMAP shows the eQTL effect size at rs10774671. UMAP coordinates are identical to Fig. 2a. **f**. Effect directions of OAS1/3 eQTLs and the risk allele of COVID-19 GWAS at the lead variant rs10774671.

We investigated our recently published PBMC scRNA-seq data^22^ obtained from 112 donors, including 84 COVID-19-positive individuals, and profiled using the CITE-seq approach^39^, as an independent in vivo validation of *OAS1* eQTL colocalization with GWAS locus for COVID-19 susceptibility (Methods). There are 18 major blood cell types annotated in this dataset (Extended Data Fig. 7a), of which myeloid cells and certain T cell subtypes show higher expression of the secondary response genes discovered in our fibroblast data (Extended Data Fig. 7b and Methods). As expected, *OAS1* is highly expressed as a secondary response gene in PBMCs (Fig. 6e). In addition, we confirmed that the *OAS1* gene is also a strong eQTL in PBMCs and colocalizes well with the COVID-19 GWAS locus with the posterior probability of 0.99 (Fig. 6b). The GWAS index variant rs10774671G>A is the lead eQTL variant in PBMCs whose alternative allele A is strongly negatively correlated with *OAS1* expression. This is especially clear in CD16^+^ monocytes, among other immune cell types (Fig. 6f and Extended Data Fig. 7a).

The index SNP rs10774671 is known to be a splicing QTL^40^ that disrupts the splicing motif right next to the last exon of the *OAS1* gene (Fig. 6g). In our fibroblast data, this variant also increased the intron expression between the last two exons and created three different isoforms (Fig. 6g), all of which are known to cause impaired OAS1 protein expression^40^. These alternative isoforms are also observed in nasal epithelial cells from 33 brushing samples from a recent COVID-19 cohort^23^. We confirmed that the alternative allele of the lead eQTL variant in stimulated fibroblasts is associated with lower expression of the *OAS1* gene in the epithelial cells and the occurrence of alternative isoforms, as expected (Fig. 6h). This suggests that the in vitro stimulation fibroblast system is an appropriate model to study physiologically relevant eQTLs in the context of infection, such as COVID-19.

In addition to the *OAS1* eQTL, *OAS3* eQTL in fibroblasts was also colocalized with the COVID-19 GWAS locus (posterior probability = 0.99) (Fig. 5b and Extended Data Fig. 7c). Because *OAS1* and *OAS3* are both ISGs, the expression patterns of *OAS1* and *OAS3* along the innate immune response trajectory are expected to be very similar, as indeed observed (Fig. 6c and Extended Data Fig. 7d). However, the direction of the eQTL effect was opposite for the two genes: *OAS1* gene expression is downregulated by the alternative allele of the COVID-19 GWAS index SNP rs10774671G>A, whereas the expression level of *OAS3* gene is upregulated by the alternative allele (Fig. 6d and Extended Data Fig. 7e,f).

## Discussion

In this work we developed GASPACHO, a statistical framework that fulfils two tasks: firstly, to infer the trajectory of gene expression over a dynamic process, and secondly, to model nonlinear dynamic genetic effects in every individual cell. Using GASPACHO, we integrated scRNA-seq data from fibroblasts from 68 donors triggered by innate immune stimuli and obtained a low-dimensional gene expression space representing the response dynamics across stimulated cells. This approach provides us with a unique map of interindividual transcriptional variation at single-cell resolution, which was often linked with noncoding regulatory regions (such as TF binding sites), previously profiled during fibroblast stimulation^27^. This approach discovered 1,275 eQTL loci, of which 988 were colocalized with one or more GWAS loci of autoimmune and infectious diseases, including COVID-19 at the *OAS1* locus. We also found 1,607 colocalizations shared with the Open Targets colocalizations database, of which 615 also overlap with the GTEx fibroblast colocalization analysis. We note here that some of the colocalizations in the Open Targets database are likely to be missed because Open Targets has only tested colocalizations against genome-wide significant loci in GWAS traits (*P* < 5 × 10^−8^), while we tested colocalizations for all GWAS loci (regardless of their *P* values) overlapping with 1-megabase (Mb) *cis*-windows of genes tested for eQTL mapping.

Previous studies used scRNA-seq profiling of tissues composed of several cell types from dozens to hundreds of donors, and performed genetic association mapping of cell-type-specific expression by using a pseudo-bulk approach^13–19^. GASPACHO, as well as cellRegMap^33^, allows mapping of dynamic genetic effects of gene expression in individual cells. These two approaches are particularly suitable when considering a continuous genetic effect along cellular states rather than several discrete cell populations or states. GASPACHO and CellRegMap both incorporate context-specific donor (donor by context interaction) effects to adjust for dynamic genetic effects, for a better statistical calibration (because dynamic cellular states, such as immune responses, often vary between donors due to environmental and trans genetic effects). However, CellRegMap and GASPACHO differ in their model assumptions on genetic effects: CellRegMap assumes linearity on context-specific genetic (genotype by context interaction) effects, while GASPACHO assumes that those effects are nonlinear, suggesting GASPACHO is more flexible, yet computationally intensive (section 3 of the Supplementary Notes). Therefore, further studies are required to implement faster GP regression in modern computational environments, such as GPU. Lastly, the GPLVM implemented in GASPACHO is currently applicable for rapid analysis of dozens of thousands of cells. An accelerated version of GPLVM will be needed in the future for scaling, and a cutting-edge Bayesian inference technique, such as the stochastic variational inference implemented in GPy, should be able to achieve this goal.

The innate immune response is a genetic program that is elicited by most cells invaded by pathogens; however, the response varies between infected cells in terms of magnitude, the specific set of regulated genes and their cellular fate. This variability is observed both between cells originating from different lineages and between individual cells from the same homogenous cellular population^25–27^. Furthermore, genetic variation has been shown to significantly modulate the innate immune response in many previous studies and its dynamics are thought to be nonlinear^4,6–9,35^. GASPACHO is thus particularly useful to study how genetic effects are associated with different stages and cellular trajectories during this response, as demonstrated, for example, in our analysis of primary and secondary response genes. Furthermore, we also observe eQTLs that appear only during stimulation, as previously suggested using bulk RNA-seq^4,6–9,35^, but without the need to partition samples into discretized conditions.

Our in vitro immune stimulation of dermal fibroblasts can be used as a model system to study genetic effects in innate immune responses in primary cells. Using this analysis, we detected 6,327 colocalizations between eQTLs and various autoimmune and infectious disease GWAS loci. While fibroblasts are not the primary cellular target of SARS-CoV-2 infection (which mostly targets epithelial cells), we detected a colocalization between *OAS1* eQTL and COVID-19 GWAS locus, which we then also found in PBMCs from patients with COVID-19. This colocalization was also previously found using bulk RNA-seq^41^. Our findings suggest an association between a particular risk variant (rs10774671) and COVID-19 infection and severity, and that this risk allele may generate alternative isoforms of the *OAS1* gene in nonclassical monocytes in peripheral blood. We further found that these alternative isoforms are expressed in nasal epithelial cells from a set of patients with COVID-19 carrying the alternative allele. Since the alternative allele is also a risk allele in COVID-19 GWAS, this implies that these *OAS1* RNA splicing isoforms may be associated with impaired OAS1 protein expression and viral clearance in host cells, as previously suggested in other viral diseases^40,42^. Interestingly, we also observe a colocalization in this locus between *OAS3* eQTL and COVID-19 GWAS locus; however, in this case, the alternative allele of rs10774671 is linked to an increase in the *OAS3* gene expression level. Further studies are needed to mechanistically determine the impact of OAS3 expression on SARS-CoV-2 infection.

In summary, our study demonstrates how coupling single-cell transcriptomics with a statistical approach can identify dynamic nonlinear effects of genetic variants across cellular contexts.

## Methods

### Ethical compliance

This project was approved by the Wellcome Sanger Institute Animal Welfare and Ethical Review Body and complied with all relevant ethical regulations regarding animal research and human studies. Human cells were obtained from HipSci^24^, where they were collected from volunteers recruited from the National Institute for Health and Care Research (NIHR) Cambridge BioResource (written consent was given). Human skin profiling was performed in accordance with protocols approved by the Newcastle Research Ethics Committee (REC approval 08/H0906/95+5). Patients with a confirmed diagnosis of COVID-19 were recruited from Addenbrooke’s and Royal Papworth hospitals under ethical approval obtained from the East of England Cambridge Central Research Ethics Committee (NIHR BioResource, REC no. 17/EE/0025). Informed consent was obtained for all participants.

### Dermal fibroblast cell culture and stimulation

Primary dermal fibroblast cells from HipSci were used (http://www.hipsci.org/). The cells were derived from healthy individuals spanning a range of ages (from 30 to 79 and 57.2 on average) and both sexes (40 female and 28 male). Following a similar protocol used in our previous work^27^, cells were cultured in DMEM (high glucose, pyruvate, Life Technologies), with 10% FBS, GlutaMAX and 1% penicillin-streptomycin. In each experimental batch, we cultured in parallel cells from three different individuals. Cells were split the day before the experiment into separate wells and on the day of experiment were stimulated with either dsRNA (0.5 µg ml^−1^ high-molecular-weight rhodamine-labeled Poly(I:C) (Invivogen, tlrl-pic), transfected with 1 µl ml^−1^ lipofectamine 2000 (Thermo Fisher, 11668027), for 2 or 6 h) or 1,000 U ml^−1^ human recombinant IFN-β (11410-2, PBL), for 2 or 6 h, or left untreated. In this manner, for each individual, we obtained five separate conditions.

After the relevant period of time, cells were detached by trypsinization and resuspended in PBS. Samples from the three individuals with the same treatment were then mixed (for example, ‘unstimulated’ cells from the three donors would be pooled together). The primary aim of this mixing step was to reduce downstream experimental variability between the three donors, while simultaneously streamlining the collection stage. In this manner, we obtained plates for each of the five conditions, with each having a mixture of all three individuals.

### Sorting and single-cell library preparation

Cells were sorted on a Becton Dickinson Influx into 96-well plates containing 2 µl per well of lysis buffer, as described in the Smart-Seq2 protocol^43^, or in our previous work^27^. Importantly, each 96-well plate contained cells from the same condition of all three individuals used for each experimental batch. Single cells were sorted individually (using FSC-W versus FSC-H), and apoptotic cells were excluded using DAPI. Rhodamine-positive cells were selected in the Poly(I:C) treatments. Cells from each three-plex cell pool were sorted across four plates. Reverse transcription and complementary DNA amplification were performed according to the Smart-Seq2 protocol (Picelli et al., 2014), and library preparation was performed using an Illumina Nextera kit. Samples were sequenced using paired-end 75-bp reads on an Illumina HiSeq 2500 machine. For library preparation, cells were loaded into 384-well plates. We note that cells in one of the four stimulated conditions were assigned in a 384-well plate in conjunction with cells in naïve condition (rows C, H and M).

### Smart-Seq2 data preprocessing and quality control

All sequence data were aligned to human genome assembly GRCh38 using STAR (v.2.5.3a; https://github.com/alexdobin/STAR/releases) and ENSEMBL human gene assembly 90 as the reference transcriptome. We performed adapter trimming of Tn5 transposon and PCR primer sequences using skewer (v.0.1.127; https://github.com/relipmoc/skewer) before alignment. Following alignment, we used featureCounts (v.1.5.3; http://subread.sourceforge.net/) to count fragments for each annotated gene. In total, we observed 58,394 cells, of which 22,188 cells passed the quality control criteria: the minimum number of sequenced fragments (>10,000 autosomal fragments), the minimum number of expressed genes (>500 autosomal genes), mitochondrial fragment percentage (<20%) and the library complexity (percentage of autosomal fragment counts for the top 100 highly expressed genes <30%). We also performed demuxlet^44^ (v.0.1.0; https://github.com/statgen/demuxlet) to identify the genetic origin of each cell as well as to remove doublets using the genotype data from HipSci.

### Genotype data

We obtained the SNP genotype data from HipSci^24^ (Data availability). We also genotyped 112 COVID-19 PBMC samples using the Affymetrix Axiom UK Biobank array (Data availability). We converted the genome coordinates from hg19 to GRCh38 using CrossMap (v.0.5.2; http://crossmap.sourceforge.net/). We then performed the whole-genome imputation using Beagle (v.5.1; https://faculty.washington.edu/browning/beagle/beagle.html) with the reference panel from the 1000 Genomes Project (Data availability).

### Cell viability prediction

The cell viability was predicted by the web-based tool CEVIChE (https://saezlab.shinyapps.io/ceviche/). Because the tool is designed for bulk RNA-seq data, we aggregated gene expression levels for neighboring cells based on the UMAP in Fig. 2a. We constructed 30 × 30 equispaced grids and took geometric means of logCPM (log of counts per million) values within each grid.

### GPLVM

The GASPACHO framework incorporated a GPLVM as a core model to estimate the latent variables and model parameters subsequently used in the spatial DE analysis and eQTL mapping. We assumed that the gene expression vector ${y}_{j}={(\,{y}_{{ij}}{;i}=1,\ldots ,N\,)}^{T}$ for the gene $j$ across $N$ cells is independently drawn from

$${y}_{j}\sim N({\alpha }_{j}+Z{\gamma }_{j},{{\sigma }_{j}}^{2}\varOmega )$$

$${\alpha }_{j}\sim N(0,{{{\sigma }_{j}}^{2}K}_{\theta }{K}_{B}{K}_{X})$$

$${\gamma }_{j}\sim N(\zeta ,{{\sigma }_{j}}^{2}\varDelta )$$

where ${\alpha }_{j}$ is a baseline GP governed by three different kernel matrices, periodic kernel matrix $\,{K}_{\theta }$ for the cell cycle state ($\theta$) and two other squared exponential kernel matrices ${K}_{B}$ and ${K}_{X}$ for unknown batch effects (*B*) and the target cell state (*X*), respectively. Here, $Z$ is a design matrix for the known covariates, such as donor and sequencing plates (Fig. 1b), and ${\gamma }_{j}$ is a random effect to adjust the known confounding effects whose mean and variance were defined by $\zeta$ and the diagonal matrix $\varDelta$ shared across all genes $j=1,\ldots ,J$. The residual expression was determined by the gene-specific residual variance ${{\sigma }_{j}}^{2}$ and the cell-specific residual variance $\varOmega ={\rm{diag}}({\omega }_{i}{;i}=1,\ldots ,N)$. The variance of the GP and random effect for gene *j* was properly scaled by the gene-specific residual variance ${{\sigma }_{j}}^{2}$.

The model parameters $\{\varDelta ,\varOmega ,\varSigma ,\zeta \,\}$ and the latent variables $\{\theta ,B,X\,\}$ were inferred by maximizing the marginal likelihood

$$L(\theta ,B,X,\varDelta ,\varOmega ,\varSigma ,\zeta )=\mathop{\prod }\limits_{j=1}^{J}\int p({y}_{j}{\rm{|}}{\alpha }_{j},{\gamma }_{j})p({\alpha }_{j})p({\gamma }_{j}){\mathrm{d}}{\alpha }_{j}{\mathrm{d}}{\gamma }_{j},$$

where $\varSigma ={\rm{diag}}({{\sigma }_{j}}^{2}{;j}=1,\ldots ,J\,)$. We used the L-BFGS algorithm with the analytic gradient of the likelihood function with respect to the parameters and the latent variables. In reality, the kernel matrices are not tractable for large $N$; we computed the Titsias bound using the sparse GP^21^ to approximate the above likelihood (see section 1.3 of the Supplementary Notes for more details).

### GP mixture model for gene classification

We employed a GP mixture model to perform the DE analysis in the target cell-state space defined by $X$ which was estimated by the GPLVM. Specifically, we introduced one extra GP *β*_*k*_ for the *k*th differentially expressed gene group (*k* = 1,…,*K*) to which a gene *j* belongs:

$${y}_{j}\sim N({\alpha }_{j}+{{\delta }_{{jk}}\beta }_{k}+Z{\gamma }_{j},{{\sigma }_{j}}^{2}\varOmega )$$

$${\alpha }_{j}\sim N(0,{{{\sigma }_{j}}^{2}K}_{\theta }{K}_{B})$$

$${\beta }_{k}\sim N(0,{{{\sigma }_{j}}^{2}K}_{X})$$

$${\gamma }_{j}\sim N(\zeta ,{{\sigma }_{j}}^{2}\varDelta )$$

$${\delta }_{{jk}}\sim N(0,1)$$

Here, the effect size of the GP was properly scaled by a coefficient ${\delta }_{{jk}}$ to allow the GP to be both positively and negatively correlated with the gene expression. The model parameters $\{\varDelta ,\varOmega ,\varSigma ,\zeta \,\}$ and the latent variables $\{\theta ,B,X\,\}$ were replaced by the estimators of the GPLVM. Then, we maximized the likelihood of a finite mixture of GPs:

$$\begin{array}{l}L({{\pi }_{1},\ldots ,{\pi }_{K},\beta }_{1},\ldots ,{\beta }_{K})\\=\mathop{\prod }\limits_{j=1}^{J}\mathop{\sum }\limits_{k=1}^{K}{\displaystyle{\int}} {\pi }_{k}p(\,{y}_{j}{\rm{|}}{\alpha }_{j},{\beta }_{k},{\gamma }_{j},{\delta }_{{jk}})p({\alpha }_{j})p(\,{\beta }_{j})p({\gamma }_{j})p({\delta }_{{jk}}){\mathrm{d}}{\alpha }_{j}{{\mathrm{d}}\gamma }_{j}{\mathrm{d}}{\delta }_{{jk}}\end{array}$$

with respect to ${\pi }_{k}$ and ${\beta }_{k}$ for $k=1,\ldots ,K$. Note that the number of total mixture components $K$ is fixed in the current implementation and *K* = 3 was used in the fibroblast data. We used the sparse approximation to make the likelihood tractable (see section 1.4 of the Supplementary Notes for more details). Note that this model can be readily extended to classify dynamic eQTL effect sizes into finite spatial patterns (see section 1.4.1 of the Supplementary Notes).

For the pseudotime analysis, we computed the posterior mean *E*[*β*_*k*_|*y*_1_,…,*y*_*J*_] for the *k*th component, which provided the underlying cellular states regarding the primary and secondary innate immune responses.

### GP regression for association mapping

We employed a GP regression model to map eQTLs in the target cell-state space defined by *X* which was estimated by the GPLVM. Specifically, we introduced one extra GP *β*_*jl*_ for the gene *j* multiplied by the *l*th genetic variant *g*_*l*_ = (*g*_*l1*_,…,*g*_*lN*_)^*T*^ whose *i*th element *g*_*li*_ is alternative allele dosages for the individual *i* as a gene–environment interaction:

$${y}_{j}\sim N({\alpha }_{j}+{\beta }_{{jl}}\odot {g}_{l}+Z{\gamma }_{j},{{\sigma }_{jl}}^{2}\varOmega )$$

$${\alpha }_{j}\sim N(0,{{{\sigma }_{jl}}^{2}K}_{\theta }{K}_{B}{K}_{X})$$

$${\beta }_{{jl}}\sim N(0,{{\delta }_{g}}^{2}{{\sigma }_{jl}}^{2}(1{1}^{T}+K_{X}))$$

$${\gamma }_{j}\sim N(\zeta ,{{\sigma }_{jl}}^{2}\varDelta )$$

Here the eQTL effect size was properly scaled by a coefficient ${\delta }_{g}$ to allow for controlling of the genetic contribution on the expression level. The model parameters $\{\varDelta ,\varOmega ,\varSigma ,\zeta \,\}$ and the latent variables $\{\theta ,B,X\,\}$ were replaced by the estimated values obtained by the GPLVM. The Bayes factor of genetic association can be obtained by:

$${\mathrm{BF}}=\frac{\int p(\,{y}_{j}{\rm{|}}{\alpha }_{j},{\beta }_{{jl}},{\gamma }_{j})p({\alpha }_{j})p(\,{\beta }_{{jl}})p({\gamma }_{j}){\mathrm{d}}{\alpha }_{j}{\mathrm{d}}{\beta }_{{jl}}{\mathrm{d}}{\gamma }_{j}}{\int p(\,{y}_{j}{\rm{|}}{\alpha }_{j},{\beta }_{{jl}}=0,{\gamma }_{j})p({\alpha }_{j})p({\gamma }_{j}){\mathrm{d}}{\alpha }_{j}{\mathrm{d}}{\gamma }_{j}}$$

where we set ${\delta }_{g}=0.1$ (see section 1.5 of the Supplementary Notes for more details).

As is implemented in CellRegMap, our model can also be extended to take the context-specific donor (context-by-donor interaction) effect into account. Here, the gene expression model can be written as:

$${y}_{j}\sim N\left({\alpha }_{j}+{\beta }_{{jl}}\odot {g}_{l}+Z{\gamma }_{j}+\mathop{\sum }\limits_{i=1}^{{N}_{\mathrm{d}}}{f}_{{ij}}\odot {z}_{i},{{\sigma }_{j}}^{2}\varOmega\right )$$

$${f}_{{ij}}\sim N(0,{{{\delta }_{{\mathrm{dxc}}}}^{2}{{\sigma }_{j}}^{2}K}_{X}){\rm{;}}\;i=1,\ldots ,{N}_{d},$$

where ${f}_{{ij}}$ denotes an additional GP for the individual *i*, ${z}_{i}$ denotes the indicator vector to specify which cells belong to the individual *i* and ${N}_{\mathrm{d}}$ denotes the number of donors in the data. The additional variance parameter ${\delta }_{{\mathrm{dxc}}}$ for the context-by-donor interaction effect is estimated under the null model using all genes (see section 1.5.1 of the Supplementary Notes for more details). All the real data analyses using the fibroblast data in this manuscript were based on the Bayes factors with this context-specific donor effect.

The eQTL effect size was estimated using the posterior distribution $p(\,{\beta }_{{jl}}|\,{y}_{j})\propto p(\,{y}_{j}|\,{\beta }_{{jl}})p(\,{\beta }_{{jl}})$ and the posterior mean $E[{\beta }_{{jl}}|\,{y}_{j}]$ was computed for each variant *l* and used for the visualization on a UMAP (see section 1.5.2 of the Supplementary Notes for more details).

### Hierarchical model for eQTL mapping and enrichment analysis

We tested genetic variants whose MAF is greater than 0.05 in a 1-Mb *cis*-regulatory window centered at each gene TSS. To control the FDR in a Bayesian framework, we used the hierarchical model^45^ to obtain the posterior probability that a gene is an eQTL as well as the posterior probability that a variant is an eQTL variant within the *cis* window. The model allows incorporating various genomic annotations in the gene-level and variant-level as demonstrated previously^45^. We used the ChIP–seq peak annotations obtained by Hagai et al.^27^ in conjunction with TSS proximity to estimate the contribution of epigenetic information to the eQTL variant discovery (see section 2.1 of the Supplementary Notes for more details). Note that we only consider genes expressed in at least 10% of the cells, resulting in a tested dataset of 10,748 genes. We did not introduce the gene-level prior probability to weight highly expressed genes for the eQTL discovery.

### eQTL enrichment in differentially expressed genes and other annotations

The enrichment analysis was carried out based on the posterior probability *Z*_*j*_ that the gene *j* is an eQTL obtained from the hierarchical model. We then computed a 2 × 2 table using a corresponding binary annotation ${X}_{j}$ (if the gene *j* belongs to some annotation, for example, a TATA-box, then ${X}_{j}=1$, and otherwise ${X}_{j}=0$) or alternatively the posterior probability ${X}_{j}\in [\mathrm{0,1}]$ that the gene *j* is a differentially expressed gene (one of multiple differentially expressed gene categories defined above), such that

$${T}_{{kl}}=\mathop{\sum }\limits_{j=1}^{J}{(1-{X}_{j})}^{(1-k)}{(1-{Z}_{j})}^{(1-l)}{{X}_{j}}^{k}{{Z}_{j}}^{l}$$

for $k,l=\mathrm{0,1}$. From the 2-by-2 table $T$, we computed the log odds ratio $r=\,\log ({T}_{00}{T}_{11}/({T}_{01}{T}_{10}))$ and its standard error ${\mathrm{Var}}(r)=(1/{T}_{00}+1/{T}_{01}+1/{T}_{10}+1/{T}_{11})$ to perform hypothesis testing. The confidence interval of the log odds ratio was given by $r\pm 1.96\sqrt{{\mathrm{Var}}(r)}$. We also computed the *P* value from the *Χ*^2^ statistic ${\chi }^{2}={r}^{2}/{\rm{Var}}(r)$.

If the occurrence of eQTLs and an annotation $X$ were confounded by a factor $C$ (such as expression level for a gene), we split genes into 100 quantile bins according to the confounding factor $C$ to compute the log odds ratio and its standard error for each bin as demonstrated above, and then we combined them using the inverse variance method to derive the meta statistic for an adjusted enrichment statistic.

### eQTL sharing with GTEx tissues

We used the pairwise hierarchical model^45,46^ to jointly map eQTLs in two different cell types (a similar approach to the pairwise fGWAS model^47^). We employed the association Bayes factor at each variant for each gene to compute the regional Bayes factors in a *cis* region of 1 Mb centered at the TSS under the following five different hypotheses:

*H*_0_: a gene is not an eQTL in cell/tissue types 1 and 2.

*H*_1_: a gene is an eQTL in cell/tissue type 1, but not in cell/tissue type 2.

*H*_2_: a gene is an eQTL in cell/tissue type 2, but not in cell/tissue type 1.

*H*_3_: a gene is an eQTL in cell/tissue types 1 and 2 with two independent putative causal variants.

*H*_4_: a gene is an eQTL in cell/tissue types 1 and 2 with the shared putative causal variant.

Those regional Bayes factors were used in a hierarchical model to estimate prior probabilities that eQTLs are shared between two cell types (see section 2.2 of the Supplementary Notes for more details).

The hypothesis testing of eQTL enrichment in different DE categories, which are also colocalized with other eQTLs (such as GTEx fibroblasts), was performed by computing the pseudo counts:

$${T}_{{kl}}=\mathop{\sum }\limits_{j=1}^{J}{(1-{X}_{j})}^{(1-k)}{({{Z}_{j}}^{(1)}+{{Z}_{j}}^{(3)})}^{(1-l)}{{X}_{j}}^{k}{({Z_{j}}^{(4)})}^{l}$$

for $k,l=\mathrm{0,1}$, where ${X}_{j}$ denotes the posterior probability that the gene *j* is differentially expressed, ${{Z}_{j}}^{(1)}$ denotes the probability that the gene *j* is an eQTL in our data and not in a GTEx tissue, ${{Z}_{j}}^{(3)}$ denotes the probability that the gene *j* is an eQTL in our data and a GTEx tissue and not sharing the putative causal variant, and ${{Z}_{j}}^{(4)}$ denotes the probability that the gene *j* is an eQTL both in our data and a GTEx tissue and colocalized. The odds ratio and its standard error were computed as described in the section ‘eQTL enrichment in differentially expressed genes and other annotations’.

### Annotating TATA and CpG genes

To look for TATA-box motifs in gene promoters, we used TATA-motifs from CIS-BP (Data availability). We used the CpG annotation (Data availability) from the UCSC Genome browser to search for genes whose promoters overlap with a CGI. In both cases, we used the region 100 bp upstream from the TSS as the promoter region and referred to these genes as TATA genes and CpG genes, respectively.

### eQTL variant enrichment at TF motifs

The hierarchical model provided the posterior probability that each variant *l* in the *cis*-regulatory region for the gene *j* is the eQTL ${Z}_{{jl}}$, so that ${\sum }_{l=1}^{{L}_{j}}{Z}_{{jl}}=1$ where *L*_*j*_ is the number of variants in the *cis* window. We first selected the lead eQTL variant according to the posterior probability for each gene *j*. We then used the position weight matrices of TF motifs in CIS-BP (Data availability) to call motifs overlapping with lead eQTL variants as described elsewhere^45^.

To perform the hypothesis testing that a TF motif is significantly overlapping with eQTL variants, we set ${Z}_{j}=\mathop{\mathrm{max}}\limits_{l=1,\ldots ,{L}_{j}}\{{Z}_{jl}\}$ and *X*_*j*_ to be the binary variable whose value is *X*_*j*_ = 1 if the lead eQTL variant *l* is overlapping with a TF motif; otherwise, *X*_*j*_ = 0. We then computed the 2 × 2 table to perform the enrichment analysis as described in the section ‘eQTL enrichment in differentially expressed genes and other annotations’.

### GWAS summary statistics

GWAS summary statistics were obtained from Open Targets which collected and harmonized summary statistics from the GWAS Catalog, FinnGen and UK Biobank (in total, 4,744 traits) (Data availability). We also downloaded summary statistics of four different COVID-19-related traits for all samples excluding 23andMe (‘Very severe respiratory confirmed covid vs. population’, ‘Hospitalized covid vs. not hospitalized covid’, ‘Hospitalized covid vs. population’ and ‘Covid vs. population’ in release 5) from the COVID-19 Host Genetics Initiative (Data availability). We selected 701 GWAS traits out of 4,748 traits with the criterion of five or more genome-wide significant loci, of which 112 were broadly immune-related, including autoimmune and chronic inflammatory diseases as well as infectious diseases.

### Colocalization with GWAS traits

We used the same pairwise hierarchical model as in the section ‘eQTL sharing with GTEx tissues’ to perform the GWAS colocalization analysis, where the prior probabilities of the pairwise hierarchical model were fixed as $\{{\varPi }_{1},\,{\varPi }_{2},\,{\varPsi }_{12}\}=\{0.2,\,0.05,\,0.01\}$, so that we can compare different studies with different statistical power to detect GWAS associations due to varying sample sizes. Here, ${\varPi }_{1}$ denotes the prior probability that a gene is an eQTL, ${\varPi }_{2}$ denotes that a genomic region for the corresponding gene (a 1-Mb window centered at TSS) is a significant GWAS locus and ${\varPsi }_{12}$ is a prior probability that the eQTL and the GWAS locus are colocalized. To fit the model, we converted the effect sizes and standard errors of each GWAS trait into Bayes factors using Wakefield’s approximation^48^. See section 2.3 of the Supplementary Notes for more details.

### PBMC data analysis and eQTL mapping

We used human PBMC scRNA-seq data^22^ from 112 donors, including 84 COVID-19-positive individuals, profiled with the CITE-seq approach from 10x Genomics. We reduced the full GASPACHO approach to accommodate the PBMC single-cell data of over 700,000 cells in a reasonable time scale. The kernel functions used in the model were restricted to the linear kernel without the cyclic kernel for the cell cycle effect. The latent factors were estimated with the covariates of the number of genes expressed, the number of mapped reads, the sequencing center, sex, age, COVID-19 status, COVID-19 severity, patient ID and the first three genotype principal components. The latent factors were then used to define the two GPs

$${\alpha }_{j}\sim N(0,{{\sigma }_{j}}^{2}X{X}^{T})$$

$${\beta }_{{jl}}\sim N(0,{{\delta }_{g}}^{2}{{\sigma }_{j}}^{2}(1{1}^{T}+X{X}^{T}))$$

for the intercept and the eQTL effect size of variant *l* for gene *j*.

### *OAS1* locus analysis using COVID-19 nasal brushing samples

To fine-map the *OAS1* locus in cells in vivo infected with COVID-19, we used human single-cell data of 33 nasal brushing samples from patients with COVID-19 from a recent work^23^, profiled using CITE-seq. We used the aligned bam files to quantify allele-specific expression at rs10774671 using RASQUAL allele-specific expression caller^49^. The genotypes were assigned by fitting a binomial distribution on the allele-specific expression with probability parameters *p* = {0.01, 0.5, 0.99} for reference homozygote, heterozygote and alternative homozygote, respectively. We inferred that there are three reference homozygotes, 18 heterozygotes and 12 alternative homozygotes at rs10774671 in this dataset.

### Reporting summary

Further information on research design is available in the Nature Portfolio Reporting Summary linked to this article.

## Online content

Any methods, additional references, Nature Portfolio reporting summaries, source data, extended data, supplementary information, acknowledgements, peer review information; details of author contributions and competing interests; and statements of data and code availability are available at 10.1038/s41588-023-01421-y.

## Supplementary information

Supplementary Information Supplementary Notes sections 1–3.

Reporting Summary

## Data Availability

All Smart-Seq2 cram files of our fibroblast data are available from the European Nucleotide Archive (Accession ID: PRJEB20147). The genotype data of fibroblast samples are under managed access and available through the HipSci portal (https://www.hipsci.org/data). The lines used in this study have the identifiers: HPSI0114pf-eipl, HPSI0114pf-fikt, HPSI0114pf-joxm, HPSI0114pf-lexy, HPSI0114pf-rozh, HPSI0114pf-vabj, HPSI0114pf-vass, HPSI0114pf-zoxy, HPSI0115pf-gifk, HPSI0115pf-melw, HPSI0115pf-zihe, HPSI0214pf-feec, HPSI0214pf-heja, HPSI0214pf-pelm, HPSI0215pf-deyz, HPSI0215pf-fawm, HPSI0215pf-hipn, HPSI0215pf-oilg, HPSI0314pf-bubh, HPSI0314pf-cuhk, HPSI0314pf-qonc, HPSI0314pf-wigw, HPSI0314pf-xugn, HPSI0414pf-ceik, HPSI0414pf-gesg, HPSI0414pf-naju, HPSI0414pf-oaqd, HPSI0514pf-fiaj, HPSI0514pf-kuco, HPSI0514pf-puie, HPSI0514pf-rutc, HPSI0514pf-sohd, HPSI0514pf-vuna, HPSI0614pf-ciwj, HPSI0614pf-miaj, HPSI0614pf-oicx, HPSI0714pf-pipw, HPSI0913pf-diku, HPSI0913pf-eika, HPSI0913pf-lise, HPSI0914pf-euts, HPSI0914pf-kajh, HPSI0914pf-laey, HPSI1013pf-garx, HPSI1013pf-jogf, HPSI1013pf-pamv, HPSI1013pf-sebz, HPSI1013pf-wopl, HPSI1013pf-wuye, HPSI1014pf-qayj, HPSI1014pf-sehl, HPSI1014pf-tixi, HPSI1014pf-toss, HPSI1014pf-tuju, HPSI1014pf-vils, HPSI1113pf-bima, HPSI1113pf-dons, HPSI1113pf-eofe, HPSI1113pf-ieki, HPSI1113pf-oaaz, HPSI1113pf-qolg, HPSI1113pf-wahn, HPSI1113pf-wetu, HPSI1114pf-ualf, HPSI1213pf-hehd, HPSI1213pf-nusw, HPSI1213pf-tolg and HPSI1213pf-xuja. The genotype data of COVID-19 PBMC samples around OAS1 gene are available at Zenodo^50^. The genome-wide genotype data are available upon request under the managed access of the NIHR BioResource’s Data Access Committee. The annotation of the CpG site was downloaded from the UCSC website (https://hgdownload.soe.ucsc.edu/goldenPath/hg38/database/cpgIslandExt.txt.gz). The position weight matrices (PWMs) of transcription factor motifs were obtained from CIS-BP (http://cisbp.ccbr.utoronto.ca/bulk.php). The PWMs used to find TATA-box in the gene promotor have the following identifiers: M1641_1.02, M2191_1.02, M4011_1.02, M4266_1.02, M6502_1.02, M1642_1.02, M4010_1.02, M4014_1.02 and M4708_1.02. Open Targets GWAS summary statistics are available from the GWAS Catalog (https://www.ebi.ac.uk/gwas/), FINNGEN (https://www.finngen.fi/en/access_results) and UK Biobank (https://www.nealelab.is/uk-biobank). COVID-19 GWAS summary statistics (release 5) are available from the COVID-19 Host Genetics Initiative (https://www.covid19hg.org/results/r5/). The Open Targets colocalization data are obtained from the website (https://ftp.ebi.ac.uk/pub/databases/opentargets/genetics/210608/). The eQTL summary statistics of GTEx 48 tissues as well as immune cells (iPSC-derived macrophages and monocytes) under different stimulation conditions were obtained from the eQTL catalog (http://ftp.ebi.ac.uk/pub/databases/spot/eQTL/). The 1000 Genomes Project VCF data (version: shapeit2_integrated_snvindels_v2a_27022019.GRCh38.phased) were obtained from: http://hgdownload.soe.ucsc.edu/gbdb/hg38/1000Genomes/. The summary statistics of fibroblast eQTLs are available from Zenodo (10.5281/zenodo.7680146).

## Code availability

GASPACHO (v.1.0.0), example data and script code to reproduce the results are available at Zenodo^51^.
