# Supplementary material for: Mapping interindividual dynamics of innate immune response at single-cell resolution
Source: Nat Genet. 2023 Jun 12;55(6):1066–75. doi: 10.1038/s41588-023-01421-y (PMC10260404; doi:10.1038/s41588-023-01421-y)
Supplement: Supplementary file 1 — Supplementary Notes sections 1–3. [file 41588_2023_1421_MOESM1_ESM.pdf]

nature genetics

Technical Report

https://doi.org/10.1038/s41588-023-01421-y

Mapping interindividual dynamics of innate immune response at single-cell resolution

In the format provided by the authors and unedited

1 GASPACHO

The GAuSsian Processes for Association mapping leveraging Cell HeterOgeneity (GASPACHO) is an analysis framework utilising Gaussian Processes (GP) for dimensional reduction and genetic association mapping. GASPACHO consists of three different components: (1) Gaussian Process Latent Variable Model (GPLVM), (2) Gaussian Process mixture model for spatial differential expression anlaysis; and (3) Gaussian Process regression for genetic association mapping in single cell resolution. The subsequent sections demonstrate the model derivation and detailed parameter inference in an ordinary maximum likelihood framework.

1.1 Nomenclature

N : number of cells (samples)
J : number of genes (features)
M : number of inducing points
O : number of fixed variables
P : dimension of design matrix of random effects
Q : number of latent variables
$\mathcal{Y} \in \mathbb{R}^{N\times J}$: normalised expression matrix
$\mathcal{B} \in \mathbb{R}^{P\times J}$: random effects
$\mathcal{F} \in \mathbb{R}^{N\times J}$: sparse GP
$\mathcal{U} \in \mathbb{R}^{M\times J}$: inducing variables
$\mathcal{X} \in \mathbb{R}^{N\times Q}$: latent variables (N training points)
$W \in \mathbb{R}^{N\times O}$: design matrix for fixed effects
$\mathcal{A} \in \mathbb{R}^{N\times O}$: Fixed effect parameter matrix
$\mathcal{T} \in \mathbb{R}^{M\times Q}$: inducing points
$Z \in \mathbb{R}^{N\times P}$: design matrix for random effects
$\zeta \in \mathbb{R}^{P}$: mean of random effects
$\Delta \in \mathbb{R}^{P\times P}$: covariance matrix of random effects (diagonal)
$\rho \in \mathbb{R}^{P}$: length parameters of kernel
$\theta \in \mathbb{R}^{1}$: variance parameter of kernel
$\Sigma \in \mathbb{R}^{J\times J}$: residual variance matrix for genes (diagonal)
$\Omega \in \mathbb{R}^{N\times N}$: residual variance matrix for cells (diagonal)
$\theta K \in \mathbb{R}^{M\times M}$: Covariance matrix of M inducing points (kernel is defined later)
$\theta \mathcal{K}_{NM} \in \mathbb{R}^{N\times M}$: Covariance matrix between N training points and M inducing points
$\theta \mathcal{K}_{NN} \in \mathbb{R}^{N\times N}$: Covariance matrix of N training points
$1_J \in \mathbb{R}^{J}$: J dimensional vector of all 1's
$I_N \in \mathbb{R}^{N\times N}$: N dimensional identity matrix
$\mathcal{N}(Y|M,U,V)$: matrix normal distribution of random variable matrix $Y \in \mathbb{R}^{N\times P}$ with mean $M \in \mathbb{R}^{N\times P}$, row variance matrix $U \in \mathbb{R}^{N\times N}$ and column variance matrix $V \in \mathbb{R}^{P\times P}$

1.2 Kernel

We assume gene expression is affected by cell cycle and other biological and technical effects. Those effects are not known a priori, and therefore to be inferred from the data. For Q-dimensional latent vectors $x, x' \in \mathbb{R}^Q$, the kernel function specifically used is given by the product of the periodic and ARD SE kernel:

$$\theta k(x, x') = \theta \exp\left\{ -\frac{2\sin^2(|x_1 - x'_1|/2)}{\rho_1^2} \right\} \exp\left\{ -\sum_{q=2}^{Q} \frac{(x_q - x'_q)^2}{2\rho_q^2} \right\},$$

where the periodic term models the cell cycle effect and the ARD SE kernel for the other effects.

For the N training points $\mathcal{X}^\top = (x_k \in \mathbb{R}^Q; 1 \leq k \leq N)$ and M inducing points $\mathcal{T}^\top = (t_k \in \mathbb{R}^Q; 1 \leq k \leq M)$, we define the covariance matrices:

$$\begin{aligned} K &= \{k(t_k, t_l); 1 \leq k, l \leq M\}, \\ \mathcal{K}_{NM} &= \{k(x_k, t_l); 1 \leq k \leq N, 1 \leq l \leq M\}, \\ \mathcal{K}_{NN} &= \{k(x_k, x_l); 1 \leq k, l \leq N\}. \end{aligned}$$

1.3 GPLVM

Our GPLVM is an extension of Titsias and Lawrence [1] with introducing both fixed and random effect terms that internally adjust the expression matrix $\mathcal{Y}$. The joint probability of the GPLVM is written as a product of matrix normal distributions:

$$\begin{aligned} p(\mathcal{Y}, \mathcal{B}, \mathcal{F}, \mathcal{U}, \mathcal{X}) = &\ \mathcal{N}(\mathcal{Y} | W\mathcal{A} + Z\mathcal{B} + \mathcal{F}, \Omega, \Sigma) \mathcal{N}(\mathcal{B} | \zeta 1_J^\top, \Delta, \Sigma) \\ &\times \mathcal{N}(\mathcal{F} | \mathcal{K}_{NM} K^{-1} \mathcal{U}, \theta \tilde{\mathcal{K}}_{NN}, \Sigma) \mathcal{N}(\mathcal{U} | 0, \theta K, \Sigma) \mathcal{N}(\mathcal{X} | 0, I_N, I_Q), \end{aligned}$$

where $\tilde{\mathcal{K}}_{NN} = \mathcal{K}_{NN} - \mathcal{K}_{NM} K^{-1} \mathcal{K}_{MN}$. Because $\mathcal{K}_{NN}$ is not tractable for large N, we calculate the lower bound

$$\begin{aligned} \log p(\mathcal{Y} | \mathcal{B}, \mathcal{U}, \mathcal{X}) &= \log \int p(\mathcal{F} | \mathcal{U}) p(\mathcal{Y} | \mathcal{B}, \mathcal{F}, \mathcal{U}) d\mathcal{F} \\ &\geq \int p(\mathcal{F} | \mathcal{U}) \log p(\mathcal{Y} | \mathcal{B}, \mathcal{F}, \mathcal{U}) d\mathcal{F} \\ &= \log p(\mathcal{Y} | W\mathcal{A} + Z\mathcal{B} + \mathcal{K}_{NM} K^{-1} \mathcal{U}, \Omega, \Sigma) - \frac{J}{2} \mathrm{tr}(\Omega^{-1} \theta \tilde{\mathcal{K}}_{NN}) \\ &\equiv \mathcal{L}_1. \end{aligned}$$

We then have the marginal probability of $\mathcal{Y}$ given $\mathcal{X}$ as the Titsias lower bound, such that

$$\begin{aligned} p(\mathcal{Y} | \mathcal{X}) p(\mathcal{X}) &\geq p(\mathcal{X}) \int \exp\{\mathcal{L}_1\} p(\mathcal{B}) p(\mathcal{U}) d\mathcal{B} d\mathcal{U} \\ &= \mathcal{N}(\mathcal{Y} | W\mathcal{A} + Z\zeta 1_J^\top, \Omega + Z\Delta Z^\top + \theta \mathcal{K}_{NM} K^{-1} \mathcal{K}_{MN}, \Sigma) \\ &\quad \times \mathcal{N}(\mathcal{X} | 0, I_N, I_Q) \exp\{-J \mathrm{tr}(\Omega^{-1} \theta \tilde{\mathcal{K}}_{NN})/2\} \\ &\equiv \exp\{\mathcal{L}_2\}. \end{aligned}$$

We maximise $\mathcal{L}_2$ with respect to $\Theta \equiv \{\Sigma, \Omega, \mathcal{X}, \mathcal{T}, \rho, \theta, \Delta, \zeta, \mathcal{A}\}$ using the quasi Newtom method (L-BFGS) with the gradient analytically obtained.

After some matrix algebra, the Titsias bound of our model can be written as

$$\mathcal{L}_2 = -\frac{NJ}{2} \log(2\pi) - \frac{N}{2} \log|\Sigma| - \frac{J}{2} \log|V| - \frac{J}{2} \mathrm{tr}(SV^{-1}) - \frac{J}{2} \mathrm{tr}(\Omega^{-1} \theta \tilde{\mathcal{K}}_{NN}),$$

where

$$\begin{aligned} V &= \Omega + \tilde{Z}\tilde{\Delta}\tilde{Z}^\top, \\ V^{-1} &= \Omega^{-1} - \Omega^{-1}\tilde{Z}\Phi\tilde{Z}^\top\Omega^{-1}, \\ S &= (\mathcal{Y} - \tilde{W}\tilde{\mathcal{A}})\Sigma^{-1}(\mathcal{Y} - \tilde{W}\tilde{\mathcal{A}})^\top / J \end{aligned}$$

with modified matrix notations

$$\begin{aligned} \tilde{Z} &= (Z, \mathcal{K}_{NM}) \\ \tilde{\Delta} &= \begin{pmatrix} \Delta & 0 \\ 0 & \theta K^{-1} \end{pmatrix} \\ \tilde{W} &= (W, Z\zeta) \\ \tilde{\mathcal{A}} &= \begin{pmatrix} \mathcal{A} \\ 1_J^\top \end{pmatrix} \\ \Phi^{-1} &= \tilde{\Delta}^{-1} + \tilde{Z}^\top\Omega^{-1}\tilde{Z} \end{aligned}$$

to make the lower bound simpler.

The model parameters are alternatively updated to maximise the lower bound where some of the model parameters have the analytical solution at each iteration, while others don't. The following subsections provide either the first derivative of the lower bound (the gradient) or the exact solution of each model parameters where the gradient to be 0.

1.3.1 The fixed parameters $\mathcal{A}$ and ζ

The fixed parameters have the exact solution at each iteration of optimisation. The maximum of the lower bound is attained by

$$\begin{aligned} \hat{\zeta} &= (Z^\top V^{-1} Z + \lambda I_P)^{-1} Z^\top V^{-1} [(1_J^\top \Sigma^{-1} \mathcal{Y}^\top)^\top - W\mathcal{A}\Sigma^{-1}1_N] / (1_J^\top \Sigma^{-1} 1_J), \\ \hat{\mathcal{A}} &= (W^\top V^{-1} W)^{-1} W^\top V^{-1} (\mathcal{Y} - Z\zeta 1_J^\top), \end{aligned}$$

where $\lambda > 0$ is a ridge parameter to avoid the parameter undetermined, because $\mathrm{rank}(Z) < P$ in general. We set $\lambda = 0.01$ in the current implementation. Note that, when we optimise $\mathcal{L}_2$ with respect to ζ or $\mathcal{A}$, all there parameters are fixed constant at each iteration.

1.3.2 Gene specific residual variance Σ

The matrix derivative of $\mathcal{L}_2$ with respect to the precision Σ^{-1} (but not variance Σ) gives

$$\partial_{\Sigma^{-1}}\mathcal{L}_2 = \frac{N}{2}\mathrm{tr}(\Sigma\partial\Sigma^{-1}) - \frac{1}{2}\mathrm{tr}\left[\partial\Sigma^{-1}(\mathcal{Y} - \tilde{W}\tilde{\mathcal{A}})^\top V^{-1}(\mathcal{Y} - \tilde{W}\tilde{\mathcal{A}})\right],$$

suggesting the diagonal element of $(\mathcal{Y} - \tilde{W}\tilde{\mathcal{A}})^\top V^{-1}(\mathcal{Y} - \tilde{W}\tilde{\mathcal{A}})/N$ gives the optimum variance parameter. Note that it requires the matrix computation

$$\begin{aligned} (\mathcal{Y} - \tilde{W}\tilde{\mathcal{A}})^\top V^{-1}(\mathcal{Y} - \tilde{W}\tilde{\mathcal{A}}) &= \mathcal{Y}^\top\Omega^{-1}\mathcal{Y} - \mathbb{C}\tilde{\mathcal{A}} - \tilde{\mathcal{A}}^\top\mathbb{C}^\top + \tilde{\mathcal{A}}^\top(\tilde{W}^\top\Omega^{-1}\tilde{W})\tilde{\mathcal{A}} \\ &\quad - \mathbb{D}\Phi\mathbb{D}^\top + \mathbb{D}\Phi\mathbb{E}\tilde{\mathcal{A}} + \tilde{\mathcal{A}}^\top\mathbb{E}^\top\Phi\mathbb{D}^\top - \tilde{\mathcal{A}}^\top\mathbb{E}^\top\Phi\mathbb{E}\tilde{\mathcal{A}} \end{aligned}$$

where

$$\begin{aligned} \mathbb{C} &\equiv \mathcal{Y}^\top\Omega^{-1}\tilde{W}, \\ \mathbb{D} &\equiv \mathcal{Y}^\top\Omega^{-1}\tilde{Z}, \\ \mathbb{E} &\equiv \tilde{Z}^\top\Omega^{-1}\tilde{W} \end{aligned}$$

should be precomputed for a scalable optimisation.

Although the variance parameter in the original model above has the exact solution, the model can be easily extended when the parameter estimate is unstable because of many 0's in the expression data. Then we can assume the precision parameter to be Gamma distributed with the following shape and rate parameters:

$$\frac{1}{\sigma_j^2} \equiv \tau_j \sim \mathcal{G}(\nu/2, \nu\mu_j/2)$$
$$\mu_j = x_j^\top \lambda$$

so that $\mathbb{E}[1/\tau_j] = \mathbb{E}[\sigma_j^2] = \mu_j$ and x_j is an arbitrary covariate vector. We normally use the percentage p_j of cells that express each gene j and $x_j^\top = (p_j, p_j - p_j^2)$. Here the Gamma distribution is the conjugate prior on $\mathcal{L}_2$, we can analytically obtain the posterior probability distribution of τ_j:

$$\tau_j|\mathcal{Y} \sim \mathcal{G}\left(\frac{\nu+N}{2}, \frac{\nu\mu_j + SSE_j}{2}\right),$$

where SSE_j is the jth diagonal element of $(\mathcal{Y} - \tilde{W}\tilde{\mathcal{A}})^\top V^{-1}(\mathcal{Y} - \tilde{W}\tilde{\mathcal{A}})$. The hyperparameter λ can be learned from the data in a standard generalised linear model with Gamma outcome, where the dependent variable τ_j and its $\log \tau_j$ are replaced at each iteration by the posterior means

$$\mathbb{E}[\tau_j|\mathcal{Y}] = \frac{\nu+N}{\nu\mu_j + SSE_j},$$
$$\mathbb{E}[\log \tau_j|\mathcal{Y}] = \psi\left(\frac{\nu+N}{2}\right) - \log\left(\frac{\nu\mu_j + SSE_j}{2}\right),$$

where$\psi(\cdot)$ is the digamma function.

1.3.3 Cell specific residual variance Ω

Because we introduce the column variance Σ and the row variance Ω for $\mathcal{Y}$, there is a parameter uncertainty. To overcome this issue and guarantee the uniqueness of parameter estimation, we assume an inverse Gamma distribution with fixed shape and rate parameters (α and β respectively) on each cell specific residual variance for cell i (the ith diagonal element of Ω), such that

$$\omega_i^{-2} \sim \mathcal{G}(\alpha, \beta).$$

This implies that we maximise $\tilde{\mathcal{L}}_2 \equiv \mathcal{L}_2 + (\alpha - 1)\log|\Omega^{-1}| - \beta \mathrm{tr}(\Omega^{-1})$ instead of $\mathcal{L}_2$ for Ω. The first derivative with respect to Ω^{-1} is given by

$$\partial_{\Omega^{-1}}\tilde{\mathcal{L}}_2 = \frac{J}{2}\mathrm{tr}\{\Omega V^{-1}\Omega(\partial\Omega^{-1})\} - \frac{J}{2}\mathrm{tr}\{\Omega V^{-1}SV^{-1}\Omega(\partial\Omega^{-1})\} - \frac{J}{2}\mathrm{tr}\{\theta\tilde{\mathcal{K}}_{NN}(\partial\Omega^{-1})\}$$
$$+ (\alpha - 1)\mathrm{tr}\{\Omega(\partial\Omega^{-1})\} - \beta\mathrm{tr}\{\partial\Omega^{-1}\}.$$

In order to make a scalable computation, we calculate

$$\Omega V^{-1}SV^{-1}\Omega = (\mathcal{Y}\Sigma^{-1}\mathcal{Y}^\top - \mathcal{Y}\Sigma^{-1}\mathbb{D}\Phi\tilde{Z}^\top + \mathcal{Y}\Sigma^{-1}\tilde{\mathcal{A}}^\top\mathbb{F}^\top$$
$$- \tilde{Z}\Phi\mathbb{D}^\top\Sigma^{-1}\mathcal{Y}^\top + \tilde{Z}\Phi\mathbb{D}^\top\Sigma^{-1}\mathbb{D}\Phi\tilde{Z}^\top - \tilde{Z}\Phi\mathbb{D}^\top\Sigma^{-1}\tilde{\mathcal{A}}^\top\mathbb{F}^\top$$
$$+ \mathbb{F}\tilde{\mathcal{A}}\Sigma^{-1}\mathcal{Y}^\top - \mathbb{F}\tilde{\mathcal{A}}\Sigma^{-1}\mathbb{D}\Phi\tilde{Z}^\top + \mathbb{F}\tilde{\mathcal{A}}\Sigma^{-1}\tilde{\mathcal{A}}^\top\mathbb{F}^\top)/J$$

with precomputed matrices

$$\mathbb{D} = \mathcal{Y}^\top\Omega^{-1}\tilde{Z},$$
$$\mathbb{E} = \tilde{Z}^\top\Omega^{-1}\tilde{W},$$
$$\mathbb{F} = -\tilde{W} + \tilde{Z}\Phi\mathbb{E}.$$

1.3.4 Variance parameters $\tilde{\Delta}$ for the random effects and GP

There is no closed form for the solution of $\tilde{\Delta}$, therefore the first derivative

$$\begin{aligned}
\partial_{\tilde{\Delta}}\mathcal{L}_2 &= -\frac{J}{2}\mathrm{tr}\{V^{-1}(\partial_{\tilde{\Delta}}V)\} + \frac{J}{2}\mathrm{tr}\{V^{-1}SV^{-1}(\partial_{\tilde{\Delta}}V)\} - \frac{J}{2}\mathrm{tr}\{(\partial\theta)\Omega^{-1}(I-\mathcal{K}_{NM}K^{-1}\mathcal{K}_{MN})\} \\
&= -J\mathrm{tr}\{\tilde{\Delta}\tilde{Z}^\top V^{-1}(\partial\tilde{Z})\} + J\mathrm{tr}\{\tilde{\Delta}\tilde{Z}^\top V^{-1}SV^{-1}(\partial\tilde{Z})\} \\
&\quad -\frac{J}{2}\mathrm{tr}\{\tilde{Z}^\top V^{-1}\tilde{Z}(\partial\tilde{\Delta})\} + \frac{J}{2}\mathrm{tr}\{\tilde{Z}^\top V^{-1}SV^{-1}\tilde{Z}(\partial\tilde{\Delta})\} - \frac{J}{2}\mathrm{tr}\{(\partial\theta)\Omega^{-1}(I-\mathcal{K}_{NM}K^{-1}\mathcal{K}_{MN})\}
\end{aligned}$$

is used to update $\tilde{\Delta}$ by using L-BFGS algorithm, where

$$\begin{aligned}
\tilde{Z}^\top V^{-1} &= \tilde{\Delta}^{-1}\Phi\tilde{Z}^\top\Omega^{-1}, \\
\tilde{Z}^\top V^{-1}\tilde{Z} &= \tilde{\Delta}^{-1}(\tilde{\Delta}-\Phi)\tilde{\Delta}^{-1}.
\end{aligned}$$

After the matrix manipulation, we obtain

$$\partial_{\tilde{\Delta}}\mathcal{L}_2 = -\frac{J}{2}\mathrm{tr}\{\mathbb{A}(\partial\tilde{\Delta})\} + \frac{J}{2}\mathrm{tr}\{\mathbb{B}(\partial\tilde{\Delta})\} - \frac{J}{2}\mathrm{tr}\{\Omega^{-1}(I-\mathcal{K}_{NM}K^{-1}\mathcal{K}_{MN})\}(\partial\theta),$$

where

$$\begin{aligned}
\mathbb{A} &\equiv \tilde{Z}^\top V^{-1}\tilde{Z} = \tilde{\Delta}^{-1}(\tilde{\Delta}-\Phi)\tilde{\Delta}^{-1}, \\
\mathbb{B} &\equiv \tilde{\Delta}^{-1}\Phi\tilde{Z}^\top\Omega^{-1}S\Omega^{-1}\tilde{Z}\Phi\tilde{\Delta}^{-1} = \tilde{\Delta}^{-1}\mathbb{C}\Sigma^{-1}\mathbb{C}^\top\tilde{\Delta}^{-1}/J, \\
\mathbb{C} &\equiv \Phi(\mathbb{D}^\top - \mathbb{E}\tilde{\mathcal{A}}), \\
\mathbb{D} &\equiv \mathcal{Y}^\top\Omega^{-1}\tilde{Z}, \\
\mathbb{E} &\equiv \tilde{Z}^\top\Omega^{-1}\tilde{W}
\end{aligned}$$

should again be precomputed for a scalable optimisation.

1.3.5 Latent variables $\mathcal{X}$ and $\mathcal{T}$

There is also no closed form for the solution of $\mathcal{X}$, we here provide the first derivative

$$\begin{aligned}
\partial_{\mathcal{X}}\mathcal{L}_2 &= -\frac{J}{2}\mathrm{tr}\{V^{-1}(\partial_{\mathcal{X}}V)\} + \frac{J}{2}\mathrm{tr}\{V^{-1}SV^{-1}(\partial_{\mathcal{X}}V)\} - \frac{J}{2}\mathrm{tr}\{\Omega^{-1}\theta\partial_{\mathcal{X}}(I-\mathcal{K}_{NM}K^{-1}\mathcal{K}_{MN})\} \\
&= -J\mathrm{tr}\{\tilde{\Delta}\tilde{Z}^\top V^{-1}(\partial_{\mathcal{X}}\tilde{Z})\} + J\mathrm{tr}\{\tilde{\Delta}\tilde{Z}^\top V^{-1}SV^{-1}(\partial_{\mathcal{X}}\tilde{Z})\} + \frac{J}{2}\mathrm{tr}\{\Omega^{-1}\theta\partial_{\mathcal{X}}(\mathcal{K}_{NM}K^{-1}\mathcal{K}_{MN})\} \\
&= -J\mathrm{tr}\{\Phi\tilde{Z}^\top\Omega^{-1}(0,\partial_{\mathcal{X}}\mathcal{K}_{NM})\} + J\mathrm{tr}\{\Phi\tilde{Z}^\top\Omega^{-1}SV^{-1}(0,\partial_{\mathcal{X}}\mathcal{K}_{NM})\} + J\mathrm{tr}\{\theta K^{-1}\mathcal{K}_{MN}\Omega^{-1}(\partial_{\mathcal{X}}\mathcal{K}_{NM})\}
\end{aligned}$$

with

$$\begin{aligned}
\Phi\tilde{Z}^\top\Omega^{-1}SV^{-1} &= \Phi\tilde{Z}^\top\Omega^{-1}(\mathcal{Y}-\tilde{W}\tilde{\mathcal{A}})\Sigma^{-1}(\mathcal{Y}^\top\Omega^{-1} - \mathbb{D}\Phi\tilde{Z}^\top\Omega^{-1} + \tilde{\mathcal{A}}^\top\mathbb{F}^\top\Omega^{-1})/J \\
&= \Phi(\mathbb{D}^\top - \mathbb{E}\tilde{\mathcal{A}})\Sigma^{-1}(\mathcal{Y}^\top\Omega^{-1} - \mathbb{D}\Phi\tilde{Z}^\top\Omega^{-1} + \tilde{\mathcal{A}}^\top\mathbb{F}^\top\Omega^{-1})/J \\
&= \Phi(\mathbb{D}^\top\Sigma^{-1}\mathcal{Y}^\top - \mathbb{E}\mathbb{H} - \mathbb{G}\mathbb{D}\Phi\tilde{Z}^\top + \mathbb{G}\tilde{\mathcal{A}}^\top\mathbb{F}^\top)\Omega^{-1}/J,
\end{aligned}$$

where the following matrices are precomputed a priori:

$$\begin{aligned}
\mathbb{D} &= \mathcal{Y}^\top\Omega^{-1}\tilde{Z}, \\
\mathbb{E} &= \tilde{Z}^\top\Omega^{-1}\tilde{W}, \\
\mathbb{F} &= -\tilde{W} + \tilde{Z}\Phi\mathbb{E}, \\
\mathbb{G} &= (\mathbb{D}^\top - \mathbb{E}\tilde{\mathcal{A}})\Sigma^{-1}, \\
\mathbb{H} &= \tilde{\mathcal{A}}\Sigma^{-1}\mathcal{Y}^\top.
\end{aligned}$$

Likewise,

$$\partial_{\mathcal{T}}\mathcal{L}_2 = -J\mathrm{tr}\{\Phi\tilde{Z}^\top\Omega^{-1}(0,\partial_{\mathcal{T}}\mathcal{K}_{NM})\} + J\mathrm{tr}\{\Phi\tilde{Z}^\top\Omega^{-1}SV^{-1}(0,\partial_{\mathcal{T}}\mathcal{K}_{NM})\} + J\mathrm{tr}\{\theta K^{-1}\mathcal{K}_{MN}\Omega^{-1}(\partial_{\mathcal{T}}\mathcal{K}_{NM})\}.$$

1.3.6 The length parameter ρ in the kernel

Similar calculation is also required for the length parameter of the kernel. The first derivative with respect to ρ is then given by

$$\begin{aligned}\partial_\rho\mathcal{L}_2 = & -J\mathrm{tr}\{\Phi\tilde{Z}^\top\Omega^{-1}(0,\partial\mathcal{K}_{NM})\} + J\mathrm{tr}\{\Phi\tilde{Z}^\top\Omega^{-1}SV^{-1}(0,\partial\mathcal{K}_{NM})\} + J\mathrm{tr}\{\theta K^{-1}\mathcal{K}_{MN}\Omega^{-1}(\partial\mathcal{K}_{NM})\} \\ & + \frac{J}{2}\mathrm{tr}\left\{(\tilde{\Delta}-\Phi)\begin{pmatrix} 0 & 0 \\ 0 & \theta^{-1}\partial K\end{pmatrix}\right\} - \frac{J}{2}\mathrm{tr}\left\{\Phi\tilde{Z}^\top\Omega^{-1}S\Omega^{-1}\tilde{Z}\Phi\begin{pmatrix} 0 & 0 \\ 0 & \theta^{-1}\partial K\end{pmatrix}\right\} \\ & - \frac{J}{2}\mathrm{tr}\{\theta K^{-1}\mathcal{K}_{MN}\Omega^{-1}\mathcal{K}_{NM}K^{-1}(\partial K)\}\end{aligned}$$

where

$$\begin{aligned}\Phi\tilde{Z}^\top\Omega^{-1}S\Omega^{-1}\tilde{Z}^\top\Phi &= \Phi(\mathbb{D}^\top - \mathbb{E}\tilde{\mathcal{A}})\Sigma^{-1}(\mathbb{D}^\top - \mathbb{E}\tilde{\mathcal{A}})^\top\Phi/J, \\ \mathbb{D} &= \mathcal{Y}^\top\Omega^{-1}\tilde{Z}, \\ \mathbb{E} &= \tilde{Z}^\top\Omega^{-1}\tilde{W}.\end{aligned}$$

1.4 GP mixture model for spatial differential expression

Once we fit the GPLVM and obtained the kernel parameters and latent variables, we then split $\mathcal{X}$ into the target cell state $\mathcal{X}_1$ and the rest $\mathcal{X}_2$ for other technical or unexpected biological variations including cell cycle variation (likewise $\mathcal{T}$ into $\mathcal{T}_1$ and $\mathcal{T}_2$). Then we compute the kernel matrices

$$\begin{aligned}\mathcal{K}_{NN}^{(i)} &= k_i(\mathcal{X}_i,\mathcal{T}_i), \\ \mathcal{K}_{NM}^{(i)} &= k_i(\mathcal{X}_i,\mathcal{T}_i), \\ K^{(i)} &= k_i(\mathcal{T}_i,\mathcal{T}_i),\end{aligned}$$

for $i = 1, 2$, where

$$\begin{aligned}\hat{\theta}k_1(x,x') &= \hat{\theta}\exp\left\{-\sum_{q\in Q_1}\frac{(x_q-x'_q)^2}{2\rho_q^2}\right\}, \\ \hat{\theta}k_2(x,x') &= \hat{\theta}\exp\left\{-\frac{2\sin^2(|x_1-x'_1|/2)}{\rho_1^2}\right\}\exp\left\{-\sum_{q\in Q_2}\frac{(x_q-x'_q)^2}{2\rho_q^2}\right\},\end{aligned}$$

and Q_1 denotes the index set of target latent variables and Q_2 is the complement set.

Let us denote by $\tilde{y}_j \equiv (y_j - W\hat{a}_j - Z\hat{\zeta})/\hat{\sigma}_j$ the normalised expression for gene j. We assume the gene j belongs to one of the C differential expression categories in the target space, in which f_c denotes a GP capturing the cth spatial expression pattern ($c = 1, \ldots, C$). Specifically, we model

$$\begin{aligned}\tilde{y}_j|f_c,f_j,\delta_{cj},b_j &\sim \mathcal{N}(\delta_{cj}f_c + f_j + Zb_j, \hat{\Omega}) \\ f_c &\sim \mathcal{N}(0,\hat{\theta}\mathcal{K}_{NN}^{(1)}) \\ f_j &\sim \mathcal{N}(0,\hat{\theta}\mathcal{K}_{NN}^{(2)}) \\ b_j &\sim \mathcal{N}(\hat{\zeta},\hat{\Delta}) \\ \delta_{cj} &\sim \mathcal{N}(0,1)\end{aligned}$$

where δ_{cj} is a coefficient to allow the direction and magnitude of the cth spatial component varying across genes. Note that $C = 3$ in our result.

In reality, $\mathcal{K}^{(1)}_{NN}$ or $\mathcal{K}^{(2)}_{NN}$ are not tractable for large N, we introduce the inducing variables β_c and u_j, such that

$$\begin{aligned} f_c|\beta_c &\sim \mathcal{N}(X\beta_c, \hat{\theta}\tilde{\mathcal{K}}^{(1)}_{NN}), \\ f_j|u_j &\sim \mathcal{N}(\mathcal{K}^{(2)}_{NM}{K^{(2)}}^{-1}u_j, \hat{\theta}\tilde{\mathcal{K}}^{(2)}_{NN}), \\ \beta_c &\sim \mathcal{N}(0, \hat{\theta}K^{(1)}), \\ u_j &\sim \mathcal{N}(0, \hat{\theta}K^{(2)}), \end{aligned}$$

with $X = \mathcal{K}^{(1)}_{NM}{K^{(1)}}^{-1}$ to compute the lower bound

$$\begin{aligned} \log p(\tilde{y}_j|\beta_c, u_j, \delta_{cj}, b_j) &= \log \int p(\tilde{y}_j|f_c, f_j, \delta_{cj}, b_j)p(f_c|\beta_c)p(f_j|u_j)df_c df_j \\ &\geq \int \log p(\tilde{y}_j|f_c, f_j, \delta_{jc}, b_j)p(f_c|\beta_c)p(f_j|u_j)df_c df_j \\ &= \log \mathcal{N}(\tilde{y}_j|\delta_{cj}\bar{f}_c + \bar{f}_j + Zb_j, \hat{\Omega}) - \frac{1}{2}\mathrm{tr}\{\hat{\Omega}^{-1}\hat{\theta}(\delta^2_{cj}\tilde{\mathcal{K}}^{(1)}_{NN} + \tilde{\mathcal{K}}^{(2)}_{NN})\} \\ &\equiv \mathcal{L}^{(1)}_{cj}. \end{aligned}$$

Then Titsias lower bounds can be obtained by

$$\begin{aligned} p_0(\tilde{y}_j) &\geq \int \exp\{\mathcal{L}^{(1)}_{cj}|_{\beta_c=0,\delta_{cj}=0}\}p(u_j)p(b_j)du_j db_j \\ &= \mathcal{N}(\tilde{y}_j|0, \hat{\Omega} + Z\hat{\Delta}Z^\top + \hat{\theta}\mathcal{K}^{(2)}_{NM}{K^{(2)}}^{-1}\mathcal{K}^{(2)}_{NM}) \exp\left[-\frac{1}{2}\mathrm{tr}\{\hat{\Omega}^{-1}\hat{\theta}\tilde{\mathcal{K}}^{(2)}_{NN}\}\right] \\ &\equiv \exp\{\mathcal{L}^{(2)}_j\}, \\ p(\tilde{y}_j|\beta_c, \delta_{cj}) &\geq \int \exp\{\mathcal{L}^{(1)}_{cj}\}p(u_j)p(b_j)du_j db_j \\ &= \mathcal{N}(\tilde{y}_j|\delta_{cj}X\beta_c, \hat{\Omega} + Z\hat{\Delta}Z^\top + \hat{\theta}\mathcal{K}^{(2)}_{NM}{K^{(2)}}^{-1}\mathcal{K}^{(2)}_{NM}) \exp\left[-\frac{1}{2}\mathrm{tr}\{\hat{\Omega}^{-1}\hat{\theta}(\delta^2_{cj}\tilde{\mathcal{K}}^{(1)}_{NN} + \tilde{\mathcal{K}}^{(2)}_{NN})\}\right] \\ &\equiv \exp\{\mathcal{L}^{(2)}_{cj}\}. \end{aligned}$$

The complete log likelihood of the mixture model is then written as

$$\mathcal{L} = \sum_{j=1}^{J} w_{0j}\mathcal{L}^{(2)}_j + w_{0j}\log \pi_0 + \sum_{c=1}^{C}\sum_{j=1}^{J}[w_{cj}\mathcal{L}^{(2)}_{cj} + w_{cj}\log \pi_c] + \sum_{c=1}^{C}\log p(\beta_c) + \sum_{c=1}^{C}\sum_{j=1}^{J}\log p(\delta_{cj}).$$

We use EM-algorithm to maximise the likelihood. The E-step computes

$$\begin{aligned} \mathbb{E}_{\beta_c|\tilde{\mathcal{Y}}}\left[\mathcal{L}^{(2)}_{cj}\right] &= -\frac{N}{2}\log(2\pi) - \frac{1}{2}\log|V| - \frac{1}{2}(\tilde{y}_j - \delta_{cj}X\overline{\beta}_c)^\top V^{-1}(\tilde{y}_j - \delta_{cj}X\overline{\beta}_c) \\ &\quad - \frac{1}{2}\mathrm{tr}\left\{\hat{\Omega}^{-1}\hat{\theta}(\delta^2_{cj}\tilde{\mathcal{K}}^{(1)}_{NN} + \tilde{\mathcal{K}}^{(2)}_{NN})\right\} - \frac{1}{2}\mathrm{tr}\left\{\delta^2_{cj}X^\top V^{-1}X\mathbb{V}_{\beta_c}\right\}, \\ \mathbb{E}_{\delta_{cj}|\tilde{\mathcal{Y}}}\left[\mathcal{L}^{(2)}_{cj}\right] &= -\frac{N}{2}\log(2\pi) - \frac{1}{2}\log|V| - \frac{1}{2}(\tilde{y}_j - \overline{\delta}_{cj}X\beta_c)^\top V^{-1}(\tilde{y}_j - \overline{\delta}_{cj}X\beta_c) \\ &\quad - \frac{1}{2}\mathrm{tr}\left[\hat{\Omega}^{-1}\hat{\theta}\left\{\mathbb{E}[\delta^2_{cj}|\tilde{\mathcal{Y}}]\tilde{\mathcal{K}}^{(1)}_{NN} + \tilde{\mathcal{K}}^{(2)}_{NN}\right\}\right] - \frac{1}{2}\mathrm{Var}(\delta_{cj}|\tilde{\mathcal{Y}})\beta_c^\top X^\top V^{-1}X\beta_c, \end{aligned}$$

$$\begin{aligned}
\mathbb{E}_{\delta_{cj}|\tilde{\mathcal{Y}}}\left[\mathbb{E}_{\beta_c|\tilde{\mathcal{Y}}}\left[\mathcal{L}^{(2)}_{cj}\right]\right] &= -\frac{N}{2}\log(2\pi) - \frac{1}{2}\log|V| - \frac{1}{2}(\tilde{y}_j - \overline{\delta}_{cj}X\overline{\beta}_c)^\top V^{-1}(\tilde{y}_j - \overline{\delta}_{cj}X\overline{\beta}_c) - \frac{1}{2}\mathrm{Var}(\delta_{cj}|\tilde{\mathcal{Y}})\overline{\beta}_c^\top X^\top V^{-1}X\overline{\beta}_c \\
&\quad - \frac{1}{2}\mathrm{tr}\left[\hat{\Omega}^{-1}\hat{\theta}\left\{\mathbb{E}[\delta^2_{cj}|\tilde{\mathcal{Y}}]\tilde{\mathcal{K}}^{(1)}_{NN} + \tilde{\mathcal{K}}^{(2)}_{NN}\right\}\right] - \frac{1}{2}\mathrm{tr}\left\{\mathbb{E}[\delta^2_{cj}|\tilde{\mathcal{Y}}]X^\top V^{-1}X\mathbb{V}_{\beta_c}\right\} \\
&\equiv \overline{\mathcal{L}}^{(2)}_{cj}, \\
\mathbb{E}[w_{cj}|\tilde{\mathcal{Y}}] &= \frac{\pi_c\exp\{\overline{\mathcal{L}}^{(2)}_{cj}\}}{\pi_0\exp\{\mathcal{L}^{(2)}_j\} + \sum_{c=1}^C \pi_c\exp\{\overline{\mathcal{L}}^{(2)}_{cj}\}} \\
&\equiv \overline{w}_{cj},
\end{aligned}$$

where $\mathbb{E}[\beta_c|\tilde{\mathcal{Y}}] = \overline{\beta}_c$, $\mathrm{Var}[\beta_c|\tilde{\mathcal{Y}}] = \mathbb{V}_{\beta_c}$, $\mathbb{E}[\delta_{cj}|\tilde{\mathcal{Y}}] = \overline{\delta}_{cj}$, $\mathbb{E}[\delta^2_{cj}|\tilde{\mathcal{Y}}] = \mathrm{Var}(\delta_{cj}|\tilde{\mathcal{Y}}) + \overline{\delta}^2_{cj}$ and $V = \hat{\Omega} + Z\hat{\Delta}Z^\top + \hat{\theta}\mathcal{K}^{(2)}_{NM}K^{(2)^{-1}}\mathcal{K}^{(2)}_{NM}$.

The M-step becomes

$$\begin{aligned}
\pi_c &= \frac{1}{J}\sum_{j=1}^J \overline{w}_{cj}, \\
\beta_c|\tilde{\mathcal{Y}} &\sim \mathcal{N}(\mathbb{V}_{\beta_c}X^\top V^{-1}\sum_{j=1}^J \overline{w}_{cj}\overline{\delta}_{cj}\tilde{y}_j, \mathbb{V}_{\beta_c}), \\
\delta_{cj}|\tilde{\mathcal{Y}} &\sim \mathcal{N}(\mathbb{V}_{\delta_{cj}}\overline{w}_{cj}\overline{\beta}_c^\top X^\top V^{-1}\tilde{y}_j, \mathbb{V}_{\delta_{cj}}),
\end{aligned}$$

where

$$\begin{aligned}
\mathbb{V}_{\beta_c} &= \left[\sum_{j=1}^J \overline{w}_{cj}\mathbb{E}[\delta^2_{cj}|\tilde{\mathcal{Y}}]X^\top V^{-1}X + (\hat{\theta}K^{(1)})^{-1}\right]^{-1}, \\
\mathbb{V}_{\delta_{cj}} &= \frac{1}{\overline{w}_{cj}\mathrm{tr}\left\{X^\top V^{-1}X(\mathbb{V}_{\beta_c} + \overline{\beta}_c\overline{\beta}_c^\top)\right\} + \overline{w}_{cj}\mathrm{tr}\{\hat{\Omega}^{-1}\hat{\theta}\tilde{\mathcal{K}}^{(1)}_{NN}\} + 1},
\end{aligned}$$

since

$$\begin{aligned}
\frac{\partial}{\partial\beta_c}\mathbb{E}_{\delta_{cj}|\tilde{\mathcal{Y}}}\left[\sum_{j=1}^J \overline{w}_{cj}\mathcal{L}^{(2)}_{cj} + \log p(\beta_c)\right] &= \sum_{j=1}^J \overline{w}_{cj}\overline{\delta}_{cj}X^\top V^{-1}\tilde{y}_j - \left[\sum_{j=1}^J \overline{w}_{cj}\mathbb{E}[\delta^2_{cj}|\tilde{\mathcal{Y}}]X^\top V^{-1}X + (\hat{\theta}K^{(1)})^{-1}\right]\beta_c, \\
\frac{\partial}{\partial\delta_{cj}}\mathbb{E}_{\beta_c|\tilde{\mathcal{Y}}}\left[\overline{w}_{cj}\mathcal{L}^{(2)}_{cj} + \log p(\delta_{cj})\right] &= \overline{w}_{cj}\overline{\beta}_c^\top X^\top V^{-1}\tilde{y}_j - \delta_{cj}\left[\overline{w}_{cj}\mathrm{tr}\left\{X^\top V^{-1}X(\mathbb{V}_{\beta_c} + \overline{\beta}_c\overline{\beta}_c^\top)\right\} + \overline{w}_{cj}\mathrm{tr}\{\hat{\Omega}^{-1}\hat{\theta}\tilde{\mathcal{K}}^{(1)}_{NN}\} + 1\right].
\end{aligned}$$

1.4.1 GP mixture for classifying eQTL effect sizes

This model can be readily extended to classify eQTL effect sizes into finite categories. The corresponding baseline and eQTL models can be given by

$$\begin{aligned}
p_0(\tilde{y}_j) &\geq \mathcal{N}(\tilde{y}_j|0, \hat{\Omega} + Z\hat{\Delta}Z^\top + \hat{\theta}\mathcal{K}_{NM}K^{-1}\mathcal{K}_{NM})\exp\left[-\frac{1}{2}\mathrm{tr}\{\hat{\Omega}^{-1}\hat{\theta}\tilde{\mathcal{K}}_{NN}\}\right] \\
&\equiv \exp\{\mathcal{L}^{(2)}_j\}, \\
p(\tilde{y}_j|\beta_c, \delta_{cj}) &\geq \mathcal{N}(\tilde{y}_j|\delta_{cj}X_j\beta_c, \hat{\Omega} + Z\hat{\Delta}Z^\top + \hat{\theta}\mathcal{K}_{NM}K^{-1}\mathcal{K}_{NM})\exp\left[-\frac{1}{2}\mathrm{tr}\{\hat{\Omega}^{-1}\hat{\theta}(\delta^2_{cj}G_j\tilde{\mathcal{K}}^{(1)}_{NN}G_j + \tilde{\mathcal{K}}_{NN})\}\right] \\
&\equiv \exp\{\mathcal{L}^{(2)}_{cj}\}.
\end{aligned}$$

where $X_j = G_j(\mathcal{K}_{NM}{}^{(1)}K^{(1)-1}, 1)$ and G_j is a diagonal matrix whose diagonal elements are genotype dosages appropriately normalised by the allele frequency. Here we assume

$$\beta_c \sim \mathcal{N}\left(0, \begin{pmatrix} \hat{\theta}K^{(1)} & 0 \\ 0 & 1 \end{pmatrix}\right)$$

for $c = 1, \ldots, C$ independently. Then we have

$$\begin{aligned}
\mathbb{E}_{\delta_{cj}|\tilde{\mathcal{Y}}}\left[\mathbb{E}_{\beta_c|\tilde{\mathcal{Y}}}\left[\mathcal{L}_{cj}^{(2)}\right]\right] &= -\frac{N}{2}\log(2\pi) - \frac{1}{2}\log|V| - \frac{1}{2}(\tilde{y}_j - \overline{\delta}_{cj}X_j\overline{\beta}_c)^\top V^{-1}(\tilde{y}_j - \overline{\delta}_{cj}X_j\overline{\beta}_c) - \frac{1}{2}\mathrm{Var}(\delta_{cj}|\tilde{\mathcal{Y}})\overline{\beta}_c^\top X_j^\top V^{-1}X_j\overline{\beta}_c \\
&\quad - \frac{1}{2}\mathrm{tr}\left[\hat{\Omega}^{-1}\hat{\theta}\left\{\mathbb{E}[\delta_{cj}^2|\tilde{\mathcal{Y}}]G_j\tilde{\mathcal{K}}_{NN}^{(1)}G_j + \tilde{\mathcal{K}}_{NN}^{(2)}\right\}\right] - \frac{1}{2}\mathrm{tr}\left\{\mathbb{E}[\delta_{cj}^2|\tilde{\mathcal{Y}}]X_j^\top V^{-1}X_j\mathbb{V}_{\beta_c}\right\} \\
&\equiv \overline{\mathcal{L}}_{cj}^{(2)},
\end{aligned}$$

where $V = \hat{\Omega} + Z\hat{\Delta}Z^\top + \hat{\theta}\mathcal{K}_{NM}K^{-1}\mathcal{K}_{NM}$. The M-step is partly replaced by

$$\begin{aligned}
\beta_c|\tilde{\mathcal{Y}} &\sim \mathcal{N}(\mathbb{V}_{\beta_c}\sum_{j=1}^{J} X_j^\top V^{-1}\overline{w}_{cj}\overline{\delta}_{cj}\tilde{y}_j, \mathbb{V}_{\beta_c}), \\
\delta_{cj}|\tilde{\mathcal{Y}} &\sim \mathcal{N}(\mathbb{V}_{\delta_{cj}}\overline{w}_{cj}\overline{\beta}_c^\top X_j^\top V^{-1}\tilde{y}_j, \mathbb{V}_{\delta_{cj}}),
\end{aligned}$$

where

$$\begin{aligned}
\mathbb{V}_{\beta_c} &= \left[\sum_{j=1}^{J}\overline{w}_{cj}\mathbb{E}[\delta_{cj}^2|\tilde{\mathcal{Y}}]X_j^\top V^{-1}X_j + (\hat{\theta}K^{(1)})^{-1}\right]^{-1}, \\
\mathbb{V}_{\delta_{cj}} &= \frac{1}{\overline{w}_{cj}\mathrm{tr}\left\{X_j^\top V^{-1}X_j(\mathbb{V}_{\beta_c} + \overline{\beta}_c\overline{\beta}_c^\top)\right\} + \overline{w}_{cj}\mathrm{tr}\{\hat{\Omega}^{-1}\hat{\theta}G_j\tilde{\mathcal{K}}_{NN}^{(1)}G_j\} + 1}.
\end{aligned}$$

1.5 GP regression for mapping eQTLs and Bayes factor calculation

The genetic association mapping model also uses the estimated model parameter of the GPLVM. Let g_l denotes the vector of genotype dosages, the ith element of which indicates the number of alternative alleles of the genetic origin of cell i at the biallelic genetic variant l. We model the genetic association as a gene-environment interaction between the genotype and the GP f_{jl} governed by the kernel $\hat{\theta}\mathcal{K}_{NN}^{(1)}$ for the target cell state:

$$\begin{aligned}
y_j|f_{jl}, f_j, b_j &\sim \mathcal{N}(v_{jl}g_l + f_{jl}\odot g_l + W\hat{a}_j + f_j + Zb_j, \sigma_{jl}^2\hat{\Omega}) \\
v_{jl} &\sim \mathcal{N}(0, \delta_g^2\sigma_{jl}^2) \\
f_{jl} &\sim \mathcal{N}(0, \delta_g^2\sigma_{jl}^2\hat{\theta}\mathcal{K}_{NN}^{(1)}) \\
f_j &\sim \mathcal{N}(0, \sigma_{jl}^2\hat{\theta}\mathcal{K}_{NN}) \\
b_j &\sim \mathcal{N}(\hat{\zeta}, \sigma_{jl}^2\hat{\Delta})
\end{aligned}$$

where δ_g^2 is an arbitrary genetic variance parameter. For a rigorous association mapping, we the gene-specific residual σ_{jl} as a free parameter.

In order to make the computation tractable, we assume

$$\begin{aligned} f_{jl}|u_{jl} &\sim \mathcal{N}(\mathcal{K}_{NM}{}^{(1)}K^{(1)}{}^{-1}u_{jl}, \delta_g^2\sigma_{jl}^2\hat{\theta}\tilde{\mathcal{K}}_{NN}^{(1)}) \\ f_j|u_j &\sim \mathcal{N}(\mathcal{K}_{NM}K^{-1}u_j, \sigma_{jl}^2\hat{\theta}\tilde{\mathcal{K}}_{NN}) \\ u_{jl} &\sim \mathcal{N}(0, \delta_g^2\sigma_{jl}^2\hat{\theta}K^{(1)}) \\ u_j &\sim \mathcal{N}(0, \sigma_{jl}^2\hat{\theta}K) \end{aligned}$$

then a lower bound can be written as

$$\begin{aligned} \log p(y_j|v_{jl}, u_{jl}, u_j, b_j) &= \log \int p(y_j|f_{jl}, f_j, b_j)p(f_{jl}|\beta_c)p(f_j|u_j)df_{jl}df_j \\ &\geq \int \log p(y_j|f_{jl}, f_j, \delta_{jc}, b_j)p(f_{jl}|\beta_c)p(f_j|u_j)df_{jl}df_j \\ &= \log \mathcal{N}(y_j|v_{jl}g_l + \bar{f}_{jl} \odot g_l + W\hat{a}_j + \bar{f}_j + Zb_j, \sigma_{jl}^2\hat{\Omega}) - \frac{1}{2}\text{tr}\{\hat{\Omega}^{-1}\hat{\theta}(\delta_g^2 G_l\tilde{\mathcal{K}}_{NN}^{(1)}G_l + \tilde{\mathcal{K}}_{NN})\} \\ &\equiv \mathcal{L}_{jl}^{(1)}, \end{aligned}$$

where $G_l = \text{diag}(g_l)$. We then arrive at the Titsias bounds:

$$\begin{aligned} p_0(y_j) &\geq \int \exp\{\mathcal{L}_{jl}^{(1)}|_{v_{jl}=0, u_{jl}=0}\}p(u_j)p(b_j)du_jdb_j \\ &= \mathcal{N}\left(y_j|W\hat{a}_j, \sigma_j^2(\hat{\Omega} + Z\hat{\Delta}Z^\top + \hat{\theta}\mathcal{K}_{NM}K^{-1}\mathcal{K}_{MN})\right) \exp\left[-\frac{1}{2}\text{tr}\{\hat{\Omega}^{-1}\hat{\theta}\tilde{\mathcal{K}}_{NN}\}\right] \\ &\equiv \exp\{\mathcal{L}_j^{(2)}\}, \\ p(y_j) &\geq \int \exp\{\mathcal{L}_{jl}^{(1)}\}p(v_{jl})p(u_{jl})p(u_j)p(b_j)du_{jl}du_jdb_j \\ &= \mathcal{N}\left(y_j|W\hat{a}_j, \sigma_{jl}^2(\hat{\Omega} + Z\hat{\Delta}Z^\top + \hat{\theta}\mathcal{K}_{NM}K^{-1}\mathcal{K}_{MN} + \delta_g^2 g_l g_l^\top + \delta_g^2\hat{\theta}G_l\mathcal{K}_{NM}^{(1)}K^{(1)}{}^{-1}\mathcal{K}_{MN}^{(1)}G_l)\right) \\ &\quad\times \exp\left[-\frac{1}{2}\text{tr}\{\hat{\Omega}^{-1}\hat{\theta}(\delta_g^2 G_l\tilde{\mathcal{K}}_{NN}^{(1)}G_l + \tilde{\mathcal{K}}_{NN})\}\right] \\ &\equiv \exp\{\mathcal{L}_{jl}^{(2)}\}. \end{aligned}$$

We then performed the inference of σ_j^2 and σ_{jl}^2 on those bounds and obtained

$$\begin{aligned} \hat{\sigma}_j^2 &= \frac{\tilde{y}_j^\top(\hat{\Omega} + Z\hat{\Delta}Z^\top + \hat{\theta}\mathcal{K}_{NM}K^{-1}\mathcal{K}_{MN})^{-1}\tilde{y}_j}{N}, \\ \hat{\sigma}_{jl}^2 &= \frac{\tilde{y}_j^\top(\hat{\Omega} + Z\hat{\Delta}Z^\top + \hat{\theta}\mathcal{K}_{NM}K^{-1}\mathcal{K}_{MN} + \delta_g^2 g_l g_l^\top + \delta_g^2\hat{\theta}G_l\mathcal{K}_{NM}^{(1)}K^{(1)}{}^{-1}\mathcal{K}_{MN}^{(1)}G_l)^{-1}\tilde{y}_j}{N}. \end{aligned}$$

Therefore the Bayes factor of genetic association is obtained by

$$\begin{aligned} \log BF_{jl} &\equiv \mathcal{L}_{jl}^{(2)} - \mathcal{L}_j^{(2)} \\ &= -\frac{N}{2}\log(\hat{\sigma}_{jl}^2) + \frac{N}{2}\log(\hat{\sigma}_j^2) - \frac{1}{2}\log|\Delta_g^{-1} + Z_l^\top\hat{\Omega}^{-1}Z_l - Z_l^\top\hat{\Omega}^{-1}\tilde{Z}\Phi\tilde{Z}^\top\hat{\Omega}^{-1}Z_l| \\ &\quad - \frac{1}{2}\log|\Delta_g| - \frac{1}{2}\text{tr}\{\hat{\Omega}^{-1}\hat{\theta}\delta_g^2 G_l\tilde{\mathcal{K}}_{NN}^{(1)}G_l\} \end{aligned} \tag{1}$$

where

$$\Delta_g = \mathrm{diag}(\delta_g^2 \theta K^{(1)}, \delta_g^2),$$
$$Z_l = (G_l \mathcal{K}_{NM}{}^{(1)} K^{(1)^{-1}}, g_l),$$
$$V_l = \hat{\Omega} + \tilde{Z}\tilde{\Delta}\tilde{Z}^\top + Z_l \Delta_g Z_l^\top,$$
$$\hat{\sigma}_{jl}^2 = (y_j - W\hat{a}_j)_j^\top V_l^{-1}(y_j - W\hat{a}_j)/N,$$

and

$$\begin{aligned} |V_l| &= |\mathrm{diag}(\tilde{\Delta}, \Delta_g)^{-1} + (\tilde{Z}, Z_l)^\top \hat{\Omega}(\tilde{Z}, Z_l)||\hat{\Omega}||\mathrm{diag}(\tilde{\Delta}, \Delta_g)| \\ &= |\Phi^{-1}||\Delta_g^{-1} + Z_l^\top \hat{\Omega}^{-1} Z_l - Z_l^\top \hat{\Omega}^{-1}\tilde{Z}\Phi\tilde{Z}^\top \hat{\Omega}^{-1} Z_l||\hat{\Omega}||\mathrm{diag}(\tilde{\Delta}, \Delta_g)|. \end{aligned}$$

1.5.1 Context specific donor effect

In order to fully calibrate the type I error rate in the dynamic eQTL mapping, we further incorporate the context specific donor effect (donor × context interaction effect):

$$f_{ij}|u_{ij} \sim \mathcal{N}(\mathcal{K}_{NM}{}^{(1)} K^{(1)^{-1}} u_{ij}, \delta_{d\times c}^2 \sigma_j^2 \hat{\theta} \tilde{\mathcal{K}}_{NN}^{(1)})$$
$$u_{ij} \sim \mathcal{N}(0, \delta_{d\times c}^2 \sigma_j^2 \hat{\theta} K^{(1)})$$

for $i = 1, \ldots, N_d$, where N_d is the number of donors used in the model. These GPs are then added to the null model:

$$y_j | f_j, u_j, \{f_{ij}\}_{i=1}^{N_d} \sim \mathcal{N}\left(W\hat{a}_j + f_j + Zb_j + \sum_{i=1}^{N_d} f_{ij} \odot z_i \middle| \sigma_j^2 \hat{\Omega} \right)$$

where z_i is an indicator vector whose element is 1 if the corresponding cell came from the donor i; otherwise 0. Then we obtain the lower bound

$$p_0(y_j) \geq \mathcal{N}\left(y_j | W\hat{a}_j, \sigma_j^2 \{\hat{\Omega} + Z\hat{\Delta}Z^\top + \hat{\theta}\mathcal{K}_{NM} K^{-1} \mathcal{K}_{MN} + \delta_{d\times c}^2 \hat{\theta}(\mathcal{K}_{NM}^{(1)} K^{(1)^{-1}} \mathcal{K}_{MN}^{(1)}) \odot (Z_d Z_d^\top)\} \right)$$
$$\exp\left[-\frac{1}{2} \mathrm{tr}\{\hat{\Omega}^{-1} \hat{\theta}(\tilde{\mathcal{K}}_{NN} + \delta_{d\times c}^2 \tilde{\mathcal{K}}_{NN}^{(1)})\} \right]$$

where $Z_d = (z_1, \ldots, z_{N_d})$.

Likewise, for the alternative model of mapping an eQTL with the variant g_l, we introduce a set of GPs:

$$f_{ijl}|u_{ijl} \sim \mathcal{N}(\mathcal{K}_{NM}{}^{(1)} K^{(1)^{-1}} u_{ijl}, \delta_{d\times c}^2 \sigma_{jl}^2 \hat{\theta} \tilde{\mathcal{K}}_{NN}^{(1)})$$
$$u_{ijl} \sim \mathcal{N}(0, \delta_{d\times c}^2 \sigma_{jl}^2 \hat{\theta} K^{(1)})$$

for $i = 1, \ldots, N_d$.

We further modified matrix notations

$$\tilde{Z} = (Z, \mathcal{K}_{NM}, \mathrm{diag}(z_1)\mathcal{K}_{NM}^{(1)}, \ldots, \mathrm{diag}(z_{N_d})\mathcal{K}_{NM}^{(1)}),$$

$$\tilde{\Delta} = \begin{pmatrix} \hat{\Delta} & 0 & 0 & \cdots & 0 \\ 0 & \hat{\theta}K^{-1} & 0 & \cdots & 0 \\ 0 & 0 & \delta_{d\times c}^2 \hat{\theta} K^{(1)-1} & \cdots & 0 \\ \vdots & \vdots & \vdots & \ddots & \vdots \\ 0 & 0 & 0 & \cdots & \delta_{d\times c}^2 \hat{\theta} K^{(1)-1} \end{pmatrix},$$

the same equation 1 above can be applied to obtain the Bayes factor adjusted by the context specific donor effect.

The additional variance parameter $\delta_{d\times c}$ is estimated by maximising the Titsias lower bound of null model across all genes:

$$\tilde{\mathcal{L}}_2 = \log \mathcal{N}(\mathcal{Y}|W\hat{\mathcal{A}} + Z\hat{\zeta}1_J^\top, \hat{\Omega} + \tilde{Z}\tilde{\Delta}\tilde{Z}^\top, \hat{\Sigma}) - \frac{J}{2}\mathrm{tr}\{\hat{\Omega}^{-1}\hat{\theta}(\tilde{\mathcal{K}}_{NN} + \delta^2_{d\times c}\tilde{\mathcal{K}}^{(1)}_{NN})\}$$

whose first and second derivatives with respect to $\delta_{d\times c}$ are given by

$$\partial_{\tilde{\Delta}}\tilde{\mathcal{L}}_2 = -\frac{J}{2}\mathrm{tr}\{\mathbb{A}(\partial\tilde{\Delta})\} + \frac{J}{2}\mathrm{tr}\{\mathbb{B}(\partial\tilde{\Delta})\} - \frac{J}{2}\mathrm{tr}\{\hat{\theta}\hat{\Omega}^{-1}(I - \mathcal{K}_{NM}{}^{(1)}K^{(1)-1}\mathcal{K}_{MN}{}^{(1)})\}(\partial\delta^2_{d\times c}),$$
$$\partial^2_{\tilde{\Delta}}\tilde{\mathcal{L}}_2 = -\frac{J}{2}\mathrm{tr}\{\mathbb{A}(\partial\tilde{\Delta})\mathbb{A}(\partial\tilde{\Delta})\},$$

where the following matrices should be precomputed for faster implementation:

$$\begin{aligned}
\mathbb{A} &\equiv \tilde{Z}^\top V^{-1}\tilde{Z} = \tilde{\Delta}^{-1}(\tilde{\Delta} - \Phi)\tilde{\Delta}^{-1},\\
\mathbb{B} &\equiv \tilde{\Delta}^{-1}\Phi\tilde{Z}^\top\Omega^{-1}S\Omega^{-1}\tilde{Z}\Phi\tilde{\Delta}^{-1} = \tilde{\Delta}^{-1}\mathbb{C}\Sigma^{-1}\mathbb{C}^\top\tilde{\Delta}^{-1}/J,\\
\mathbb{C} &\equiv \Phi(\mathbb{D}^\top - \mathbb{E}\tilde{\mathcal{A}}),\\
\mathbb{D} &\equiv \mathcal{Y}^\top\Omega^{-1}\tilde{Z},\\
\mathbb{E} &\equiv \tilde{Z}^\top\Omega^{-1}\tilde{W}
\end{aligned}$$

1.5.2 Posterior estimate of eQTL effect size

The posterior distribution of $\tilde{u}_{jl} = (u_{jl}^\top, v_{jl})^\top$ given y_j is obtained by

$$\begin{aligned}
p(\tilde{u}_{jl}|y_j) &\propto p(y_j|\tilde{u}_{jl})p(\tilde{u}_{jl})\\
&= \mathcal{N}(\tilde{y}_j|Z_l\tilde{u}_{jl}, \sigma^2_{jl}V)\mathcal{N}(\tilde{u}_{jl}|0, \sigma^2_{jl}\Delta_g),
\end{aligned}$$

where $\tilde{y}_j = y_j - W\hat{a}_j$. The posterior mean of $\tilde{u}_{jl}$ is given by

$$\mathbb{E}[\tilde{u}_{jl}|y_j] = (Z_l^\top V^{-1}Z_l + \Delta_g^{-1})^{-1}Z_l V^{-1}\tilde{y}_j \equiv \bar{u}_{jl}.$$

Therefore the posterior estimate of eQTL effect size is obtained by

$$\bar{f}_{jl} = (\mathcal{K}_{NM}{}^{(1)}K^{(1)^{-1}}, 1_N)\bar{u}_{jl}.$$

1.6 Score-based test statistics

In order to assure that the GP based eQTL mapping test statistic is well calibrated, we also implemented the score based test employed by CellRegMap [2]. The statistic is defined by

$$\mathcal{S}_{jl} = \frac{1}{\hat{\sigma}^2_j}\tilde{y}_j^\top V^{-1}Z_l\Delta'_g Z_l^\top V^{-1}\tilde{y}_j \tag{2}$$

where $\Delta'_g = \mathrm{diag}(\hat{\theta}K^{(1)}, 1)$, whose distribution is given by a mixture of χ^2 distributions with scaling factors obtained by the non-negative eigenvalues of $RZ_l^\top V^{-1}Z_l R^\top$ where $\Delta'_g = R^\top R$ is given by Cholesky decomposition. The P-value of the score statistic is computed using the Davies' exact method implemented in the R package `CompQuadForm`.

In addition, the existence of only dynamic eQTL effect is captured by

$$\mathcal{S}_{jl}^{(\text{dynamic}-\text{only})} = \frac{1}{\hat{\sigma}_j^2}\tilde{y}_j^\top V_g^{-1} G_l \mathcal{K}_{NM}{}^{(1)}(K^{(1)})^{-1}\mathcal{K}_{MN}{}^{(1)} G_l Z_l^\top V_g^{-1}\tilde{y}_j \tag{3}$$

whose distribution is given by a mixture of χ^2 distributions with scaling factors obtained by the non-negative eigenvalues of $R^{-\top}\mathcal{K}_{MN}{}^{(1)} G_l V_g^{-1} G_l \mathcal{K}_{NM}{}^{(1)} R^{-1}$ where $V_g = \hat{\Omega} + \tilde{Z}\tilde{\Delta}\tilde{Z}^\top + g_l g_l^\top$ and $K^{(1)} = R^\top R$ is given by Cholesky decomposition.

The Score-based test statistic in Eq.2 is also reduced to

$$\mathcal{S}_{jl}^{(\text{static})} = \frac{(\tilde{y}_j^\top V^{-1} g_l)^2}{\hat{\sigma}_j^2 (g_l^\top V^{-1} g_l)} \tag{4}$$

for the static eQTL effect, which follows the χ^2 distribution with one degree of freedom. These score-based test statistics were used to assess the sensitivity and specificity of mapping eQTLs using synthetic data (Extended Data Figure 2 and 3).

1.7 Model comparison with CellRegMap

CellRegMap [2] is a state-of-the-art approach to map QTLs using single-cell genomics experiments. The model has many similar features as we implemented in GASPACHO's GP regression model. CellRegMap's regression model is identical with ours, such that

$$y_j | f_{jl}, f_j, b_j \sim \mathcal{N}(v_{jl} g_l + f_{jl} \odot g_l + W a_j + f_j + Z b_j, \sigma_{jl}^2 \Omega).$$

However, the parametrisation is different:

$$\begin{aligned}
&v_{jl} : \text{ fixed effect parameter} \\
&f_{jl} \sim \mathcal{N}(0, \delta_g^2 \mathcal{X}\mathcal{X}^\top) \quad \text{(linear GxC effect)} \\
&a_j : \text{ fixed effect parameter (same as GASPACHO)} \\
&f_j \sim \mathcal{N}(0, \delta_c^2 \mathcal{X}\mathcal{X}^\top) \quad \text{(linear context effect)} \\
&b_j \sim \mathcal{N}(0, \delta_{d\times c}^2 I) \quad \text{(context-dependent donor effect)} \\
&\Omega = I \quad \text{(identity matrix for non cell-specific residual variance)}
\end{aligned}$$

Here GxC effect is linear with respect to the latent factors $\mathcal{X}$ in CellRegMap, while it is nonlinear and modeled by the ARD-SE kernel in GASPACHO's GP regression model.

The other significant difference is modeling of the donor effect which is context-dependent in CellRegMap, such that

$$ZZ^\top = \mathcal{X}\mathcal{X}^\top \odot R,$$

where R is an appropriate relatedness matrix of cells obtained from the kinship matrix of donors. On the other hand, in the reduced GASPACHO's GP regression model, we essentially hypothesized $ZZ^\top = R$, thereby the donor effect is independent from the context and constant across cellular states (defined by $\mathcal{X}$). We acknowledge that the donor effect could be context specific in reality and a lack of the donor-by-context interaction effect term will lead to an inflation of Type-I error in GASPACHO. Therefore we've also implemented an alternative model to map eQTLs while taking account of the context-specific donor effect as in section 1.5.1 in this Supplementary Notes. This model is referred to as the full model in this manuscript.

The inference approach is also different. CellRegMap uses the Score-based test approach to test $v_{jl} = 0$ and $\delta_g^2 = 0$ while estimating the variance parameters δ_c^2, $\delta_{d\times c}^2$ and σ_{jl}^2. In addition, the latent

factors $\mathcal{X}$ needs to be pre-estimated with a dimensional reduction approach (e.g., PCA, MOFA). On the other hand, GASPACHO is a Bayesian approach to compute the Bayes factor described above and variance parameters and the latent factors were estimated by the GPLVM a priori in the GASPACHO framework.

1.8 Generating synthetic data

To assess both sensitivity and specificity of the GP regression model, we draw samples from the lower bound $\mathcal{L}_1$ to generate synthetic data under the null hypothesis, such as

$$\mathcal{Y}_{\text{null}}^{(m,s)} = Z^{(m,s)}B^{(m,s)} + C^{(s)}U^{(s)} + E,$$

where

$$B^{(m,s)} \sim \begin{cases} \mathcal{N}(0,\Delta,\Sigma), & m=1, s=1,2 \text{ (static donor effect in GASPACHO)} \\ \mathcal{N}(0,\Delta\otimes I_Q,\Sigma), & m=2, s=1 \text{ (linear donor by context interaction effect in CellRegMap)} \\ \mathcal{N}(0,\Delta\otimes\theta K,\Sigma), & m=2, s=2 \text{ (nonlinear donor by context interaction effect)} \end{cases}$$

denotes the donor effect size (either static donor effect or donor-by-context interaction effect) with

$$Z^{(m,s)} = \begin{cases} Z, & m=1, s=1,2 \text{ (static donor effect in GASPACHO)} \\ (Z\otimes 1_Q^\top)\odot(1_P^\top\otimes\mathcal{X}), & m=2, s=1 \text{ (linear donor by context interaction effect in CellRegMap)} \\ (Z\otimes 1_M^\top)\odot(1_P^\top\otimes\mathcal{K}_{NM}K^{-1}), & m=2, s=2 \text{ (nonlinear donor by context interaction effect)} \end{cases}$$

and

$$U^{(s)} \sim \begin{cases} \mathcal{N}(0,I_Q,\Sigma), & s=1 \text{ (linear context effect in CellRegMap)} \\ \mathcal{N}(0,\theta K,\Sigma), & s=2 \text{ (nonlinear context effect in GASPACHO)} \end{cases}$$

denotes the context effect size with

$$C^{(s)} = \begin{cases} \mathcal{X}, & s=1 \text{ (linear context effect in CellRegMap)} \\ \mathcal{K}_{NM}K^{-1}, & s=2 \text{ (nonlinear context effect in GASPACHO)} \end{cases}$$

Here $E \sim \mathcal{N}(0,\Omega,\Sigma)$, and $\mathcal{X} \sim \mathcal{N}(0,I_N,I_Q)$ and $\mathcal{T} \sim \mathcal{N}(0,I_M,I_Q)$ are also drawn from the independent matrix normal distributions to construct $\mathcal{K}_{NM}$ and K a priori.

Under the alternative hypothesis, we considered the four different genetic effect scenarios $s = 1,\ldots,4$:

$$\mathcal{Y}_{\text{alt}}^{(m,s)} = \begin{cases} \mathcal{Y}_{\text{null}}^{(m,1)} + (\mathcal{X}V^{(1)})\odot GC_1, & s=1 \text{ (linear GxC interaction effect in CellRegMap)} \\ \mathcal{Y}_{\text{null}}^{(m,2)} + P_{\mathcal{X}}(\mathcal{K}_{NM}K^{-1}V^{(2)})\odot GC_2, & s=2 \text{ (nonlinear GxC interaction effect in GASPACHO)} \\ \mathcal{Y}_{\text{null}}^{(m,2)} + (\mathcal{K}_{NM}K^{-1}V^{(2)})\odot GC_3, & s=3 \text{ (nonlinear and linear GxC interaction effect in GASPACHO)} \\ \mathcal{Y}_{\text{null}}^{(m,2)} + GC_4, & s=4 \text{ (static effect)} \end{cases}$$

with

$$V^{(s)} \sim \begin{cases} \mathcal{N}(0,I_Q,\Sigma), & s=1 \\ \mathcal{N}(0,\theta K,\Sigma), & s=2,3 \end{cases}$$

where $G \in \mathbb{R}^{N\times J}$ is a matrix of genotype dosages whose column g_j is a vector of genotypes at the putative causal variant for the corresponding gene j. Here the projection matrix $P_{\mathcal{X}}$ maps an N-dimensional vector to the linear space orthogonal to $(1,\mathcal{X})$, that is, $P_{\mathcal{X}} = I_N - \tilde{\mathcal{X}}(\tilde{\mathcal{X}}^\top\tilde{\mathcal{X}})^{-1}\tilde{\mathcal{X}}^\top$, where $\tilde{\mathcal{X}} = (1_N,\mathcal{X})$. This projection matrix allows us to generate an artificial eQTL effect size whose average across the context space $\mathcal{X}$ becomes 0. This means that CellRegMap and pseudo-bulk approaches cannot technically capture this nonlinear effect. The coefficients $C_s \in \mathbb{R}^{J\times J}(s=1,\ldots,4)$ are diagonal matrices whose J diagonal elements specify the effect sizes c_{sj} ($j=1,\ldots,J$) of J genetic variants given explained variance ρ_j for $j=1,\ldots,J$ (see below for the detail).

We also converted the Gaussian synthetic data into discrete expression data as if it were observed from single-cell RNA-seq data. We drew read count data from a Poisson distribution whose log mean parameter is given by $\mathcal{Y}_{\text{null}}^{(m,s)}$ (or $\mathcal{Y}_{\text{alt}}^{(m,s)}$), such that

$$\mathcal{Z}_{\text{null}}^{(m,s)} \sim \text{Pois}(\exp\{\mathcal{Y}_{\text{null}}^{(m,s)}\}),$$

and then transformed back to log normalised expression as $\tilde{\mathcal{Y}}_{\text{null}}^{(m,s)} = \log(\mathcal{Z}_{\text{null}}^{(m,s)} + 1)$. Note that this Poisson distribution is, in nature, overdispersed, because we retain the error term $E \sim \mathcal{N}(0, \Omega, \Sigma)$ in the log mean. This is analogous to Gamma distribution whose mixture with Poisson distribution becomes a negative bionomial distribution. In fact, we sampled $\omega_i^2 \sim \text{Gamma}(10, 10)$ for $i = 1, \ldots, N$ and $\sigma_j^2 \sim \text{Gamma}(1, 10)$ for $j = 1, \ldots, J$, so that the sparsity of single-cell data is well mimicked.

Specifically, we used $\{\mathcal{Y}_{\text{null}}^{(m,s)}, \mathcal{Y}_{\text{alt}}^{(m,s)}; m = 1, 2 \text{ and } s = 1, 2\}$ for the comparison of GASPACHO with CellRegMap and the pseudo-bulk approach (Extended Data Figure 2), $\{\mathcal{Y}_{\text{alt}}^{(1,3)}\}$ with various numbers of cells and donors for the sensitivity and runtime analyses (Extended Data Figure 3a-d, 3m-n), $\{\mathcal{Y}_{\text{null}}^{(1,2)}, \tilde{\mathcal{Y}}_{\text{null}}^{(1,2)}\}$ to assess the impact of discretisation under the null hypothesis (Extended Data Figure 3f-i) and $\{\mathcal{Y}_{\text{alt}}^{(1,4)}, \tilde{\mathcal{Y}}_{\text{alt}}^{(1,4)}\}$ to assess the specificity of GxC interaction effect under the alternative hypothesis only with a static genetic effect (Extended Data Figure 3k-l) with the dynamic effect-only Score-based statistic in Eq.3.

1.8.1 Generating genotype matrix G

The genotype vector g_j of the genotype matrix $G = (g_1, \ldots, g_J)$ was generated from Binomial distribution with the probability parameter p_j. The genotype $\mathcal{G}_{ij} \sim \mathcal{B}(2, p_j)$ of donor i at the putative causal variant for the gene j was first drawn. Then the genotype was scaled such as

$$\mathcal{G}_{ij}^* = \frac{\mathcal{G}_{ij} - 2p_j}{\sqrt{2p_j(1 - p_j)}}$$

and finally expanded according to the donor-cell assignment matrix $Z_1 \in \mathbb{R}^{N \times N_d}$, so that

$$g_j = Z_1 \mathcal{G}_j^*$$

where $\mathcal{G}_j^* \in \mathbb{R}^{N_d}$ is the vector of genotypes across all donors and N_d denotes the number of donors.

1.8.2 Selection of the effect size matrices

The effect sizes $C_1, \ldots, C_4$ are diagonal matrices to specify the static and dynamic effects of each variant. The Let us define the projection matrix $P = I_N - 11^\top / N$ to obtain the sample variance of any vector $a \in \mathbb{R}^N$ so that $S(a) = \sum_{i=1}^N (a_i - \sum_{k=1}^N a_k / N)^2 / N = a^\top P a / N$. For the given explained variance ρ_j relative to the residual σ_j^2, such as

$$\rho_j = \frac{c_{sj}^2 S(a_{sj} \odot g_j)}{c_{sj}^2 S(a_{sj} \odot g_j) + \sigma_j^2},$$

we have

$$c_{sj} = \sqrt{\frac{\rho_j}{1 - \rho_j} \frac{\sigma_j^2}{S(a_{sj} \odot g_j)}}$$

where

$$a_{sj} = \begin{cases} \mathcal{X} v_j^{(1)}, & s = 1 \\ P_{\mathcal{X}} \mathcal{K}_{NM} K^{-1} v_j^{(2)}, & s = 2 \\ \mathcal{K}_{NM} K^{-1} v_j^{(2)}, & s = 3 \\ 1_N, & s = 4 \end{cases}$$

To compute c_{sj}, we replaced $S(a_{sj} \odot g_j)$ with $\mathbb{E}_{G,V}[S(a_{sj} \odot g_j)]$, such that

$$\begin{aligned} \mathbb{E}_{G,V}[S(a_{sj} \odot g_j)] &= \mathbb{E}_{G,V}[S(G_j a_{sj})] \\ &= \frac{1}{N} \mathbb{E}_{G,V}[\mathrm{tr}\{G_j P G_j a_{sj} a_{sj}^\top\}] \\ &= \frac{1}{N} \mathrm{tr}\{\mathbb{E}_G[\mathrm{diag}(g_j \odot g_j) - g_j g_j^\top / N] \mathbb{E}_V[a_{sj} a_{sj}^\top]\} \\ &= \frac{1}{N} \mathrm{tr}\{(I_N - Z_1 Z_1^\top / N) \mathbb{E}_V[a_{sj} a_{sj}^\top]\} \\ &= \begin{cases} \sigma_j^2 \theta \mathrm{tr}\{\mathcal{X}^\top \mathcal{X} - \mathcal{X}^\top Z_1 Z_1^\top \mathcal{X} / N\} / N, & s = 1 \\ \sigma_j^2 \theta \mathrm{tr}\{\mathcal{K}_{MN} P_{\mathcal{X}} \mathcal{K}_{NM} K^{-1} - \mathcal{K}_{MN} P_{\mathcal{X}} Z_1 Z_1^\top P_{\mathcal{X}} \mathcal{K}_{NM} K^{-1} / N\} / N, & s = 2 \\ \sigma_j^2 \theta \mathrm{tr}\{\mathcal{K}_{MN} \mathcal{K}_{NM} K^{-1} - \mathcal{K}_{MN} Z_1 Z_1^\top \mathcal{K}_{NM} K^{-1} / N\} / N, & s = 3 \\ 1 - \sum_{i=1}^{N_d} n_i^2 / N^2, & s = 4 \end{cases} \end{aligned}$$

where $G_j = \mathrm{diag}(g_j)$,

$$\mathbb{E}_V[a_{sj} a_{sj}^\top] = \begin{cases} \mathcal{X} \mathcal{X}^\top, & s = 1 \\ \theta \sigma_j^2 P_{\mathcal{X}} \mathcal{K}_{NM} K^{-1} \mathcal{K}_{MN} P_{\mathcal{X}}, & s = 2 \\ \theta \sigma_j^2 \mathcal{K}_{NM} K^{-1} \mathcal{K}_{MN}, & s = 3 \\ 1_N 1_N^\top, & s = 4 \end{cases}$$

and n_i is the number of cells for the donor i.

1.8.3 Comparing eQTL mapping approaches in Extended Data Fig. 2

We compared performance of the following four approaches with synthetic data:

1. Pseudo-bulk approach: we took the average expression level from the single cell expression y_j as

   $$\tilde{y}_j = (Z_1 y_j) / (Z_1 1_{N_d})$$

   which was regressed on $\mathcal{G}_j$.

2. GASPACHO (dynamic genetic effect without DxC): we used the score-based statistic (Eq. 2) to map dynamic eQTLs (GxC interaction effect) where the context-specific donor effects were omitted.

3. GASPACHO (dynamic genetic effect with DxC): we used the score-based statistic (Eq. 2) to map dynamic eQTLs (GxC interaction effect) where the context-specific donor effects were incorporated as in section 1.5.1 in this Supplementary Notes.

4. CellRegMap (dynamic genetic effect): we used CellRegMap's `run_interaction` to map dynamic eQTLs.

2 Bayesian pairwise hierarchical model

The Bayesian pairwise hierarchical model is a simple extension of the hierarchical model first used in mapping eQTLs [3] or the pairwise fGWAS model to estimate a shared genetic architecture between paired GWAS traits [4]. To introduce the model, we begin by constructing the hierarchical model for mapping eQTLs in a single cell/tissue type. Then we increase the number of cell/tissue types to build up the pairwise model. The prior probabilities of the model are empirically estimated from the data. We incorporate a two-stage optimisation which allows us to reduce the computational complexity and to increase the stability of model fitting process as in [5].

2.1 Hierarchical model for mapping eQTLs in single cell/tissue type

The hierarchical model for mapping eQTLs in single cell/tissue type is equivalent to the Bayesian hierarchical model proposed in [3]. We used genetic associations in cis window $\mathcal{W}_j$ of 1Mb centred at transcription start site (TSS) for each gene j. The association is measured by the Bayes factor BF_{jl} obtained from the GP regression model (described in the previous section) for each genetic variant $l \in \mathcal{W}_j$. For GTEx and other eQTL summary statistic data, we used the asymptotic Bayes factor [6] which can be easily obtained from the estimated effect size $\hat{\beta}_{jl}$ and its standard error $\hat{\sigma}_{jl}$ of a variant l on expression of gene j, such that

$$BF_{jl} = \sqrt{1 - r_{jl}} \exp \left\{ \frac{z_{jl}^2}{2} r_{jl} \right\},$$

where

$$z_{jl} = \mathcal{Z} \left(\frac{\hat{\beta}_{jl}}{\hat{\sigma}_{jl}} \right)$$

and

$$r_{jl} = \frac{W}{W + \hat{\sigma}_{jl}^2}.$$

Here we use $\mathcal{Z}(\cdot)$ to convert student t statistic into normal z statistic to deal with small sample sizes (see Supplementary Note of [5] for details).

The hierarchical model is a mixture of the following two hypotheses:

H_0 $(null)$: there are no genetic variants in $\mathcal{W}_j$ that associate with the expression of gene j;

H_1 $(eQTL)$: there is one causal variant in $\mathcal{W}_j$ that affects the expression of gene j.

We introduce the prior probability Π_1 with which a gene j is an eQTL. Assuming that there are J genes genome-wide and their expression levels are conditionally independent, the likelihood of the hierarchical model is then written as a product of mixture probability over $j = 1, \ldots, J$, such that

$$L(\Pi_1) = \prod_{j=1}^{J} \left[(1 - \Pi_1) + \Pi_1 RBF_j \right], \tag{5}$$

where RBF_j denotes the regional Bayes factor which is the genetic association for gene j averaged over all variants $l \in \mathcal{W}_j$, defined as

$$RBF_j = \frac{1}{\#\mathcal{W}_j} \sum_{l \in \mathcal{W}_j} BF_{jl}. \tag{6}$$

Note that $\#\mathcal{W}_j$ denotes the number of variants in $\mathcal{W}_j$, assuming there is one variant causal to expression of gene j. The maximum likelihood estimator $\hat{\Pi}_1$ can be obtained by a standard EM algorithm.

We can extend the RBF with variant-level annotations (such as ChIP-seq peaks) in which a variant is overlapping [3]. Let x_l be a indicator vector of the variant l to be overlapping with those annotations

and β be a coefficient vector, then the variant-level prior probability that the variant l is the causal eQTL variant for gene j is defined as

$$\pi_{jl} = \frac{\exp\{x_l^\top \beta\}}{\sum_{k \in \mathcal{W}_j} \exp\{x_k^\top \beta\}}.$$

Using the prior probability, we can now extend the RBF as

$$RBF_j = \sum_{l \in \mathcal{W}_j} \pi_{jl} BF_{jl}.$$

The coefficient vector β can be obtained as the maximum likelihood estimator in conjunction with Π_1 using a standard EM algorithm [5].

The posterior probability that the gene j is an eQTL is given by

$$Z_j = \frac{\hat{\Pi}_1 RBF_j}{(1 - \hat{\Pi}_1) + \hat{\Pi}_1 RBF_j}.$$

2.2 Hierarchical model for jointly mapping eQTLs in two cell/tissue types

We then extend the hierarchical model for a pair of two cell/tissue types. Again, we use genetic associations of variant $l \in \mathcal{W}_j$ that alters expression of gene j for two different cell/tissue types 1 and 2. We consider the following 5 different hypotheses:

- H_0 $(null)$: there are no genetic variants in $\mathcal{W}_j$ that associate with expression of gene j in either cell/tissue types;
- H_1 $(single)$: there is one causal variant in $\mathcal{W}_j$ that affects expression of gene j of cell/tissue type 1;
- H_2 $(single)$: there is one causal variant in $\mathcal{W}_j$ that affects expression of gene j of cell/tissue type 2;
- H_3 $(linkage)$: there are two independent causal variants in $\mathcal{W}_j$, one of which affects expression of gene j in cell/tissue type 1 and the other one affects expression of gene j in cell/tissue type 2, independently;
- H_4 $(colocalisation)$: there is one causal variant in $\mathcal{W}_j$ that affects expression of gene j in both two cell/tissue types simultaneously.

The likelihood of the model is given by a product of a finite mixture of the 5 different hypotheses,

$$L(\Psi_{12}, \Pi_{12}) = \prod_{j=1}^{J} \left[\Phi_0 + \sum_{h=1}^{4} \Phi_h RBF_j^{[h]} \right], \tag{7}$$

where

$$\Phi_h = \begin{cases} (1-\Psi_{12})(1-\Pi_1)(1-\Pi_2) + \Psi_{12}(1-\Pi_{12}) & h = H_0 \\ (1-\Psi_{12})\Pi_1(1-\Pi_2) & h = H_1 \\ (1-\Psi_{12})(1-\Pi_1)\Pi_2 & h = H_2 \\ (1-\Psi_{12})\Pi_1\Pi_2 & h = H_3 \\ \Psi_{12}\Pi_{12} & h = H_4 \end{cases} \tag{8}$$

denotes the prior probability that gene j belongs to one of the hypotheses $h = 0, \ldots, 4$. The prior probability Φ_h is a function of the probability Ψ_{12} that the gene j is pleiotropic in cell/tissue type 1 and 2 and the probability Π_{12} that the gene j is an eQTL driven by a same variant in $\mathcal{W}_j$. The probability Π_1 (or Π_2) is the probability that the gene j is an eQTL in cell/tissue type 1 (or cell/tissue type 2), independently from the other cell/tissue type. The maximum likelihood estimators, $\Pi_1 = \hat{\Pi}_1$ and $\Pi_2 = \hat{\Pi}_2$, are

obtained by maximising Eq.5 for cell/tissue type 1 and 2 independently, and plugged into Eq.8, so that the likelihood (Eq.7) is a function of $\{\Psi_{12}, \Pi_{12}\}$. The regional Bayes factor for a hypothesis h, $RBF_j^{[h]}$, is defined by

$$RBF_j^{[h]} = \begin{cases} RBF_j^{(1)} & h = H_1 \\ RBF_j^{(2)} & h = H_2 \\ RBF_j^{(1)} RBF_j^{(2)} & h = H_3 \\ RBF_j^{(12)} & h = H_4 \end{cases}$$

where $RBF_j^{(1)}$ (or $RBF_j^{(2)}$) denotes the regional Bayes factor of gene j being an eQTL in cell/tissue type 1 (or cell/tissue type 2) defined in Eq.6, and

$$RBF_j^{(12)} = \frac{1}{\#\mathcal{W}_j} \sum_{l \in \mathcal{W}_j} BF_{jl}^{(1)} BF_{jl}^{(2)} \tag{9}$$

denotes the joint association on gene expression j averaged over $l \in \mathcal{W}_j$ in cell/tissue type 1 and 2 under the conditional independence of gene expression in two cell/tissue types. We use a standard EM algorithm to maximise Eq.7 with respect to $\{\Psi_{12}, \Pi_{12}\}$.

The posterior probability for each hypothesis was computed from

$$Z_j^{(h)} = \begin{cases} \dfrac{\hat{\Phi}_j^{(0)}}{\hat{\Phi}_j^{(0)} + \sum_{i \in \mathcal{H}_1} \hat{\Phi}_j^{(i)} RBF_j^{(i)}} & h = H_0 \\ \dfrac{\hat{\Phi}_j^{(h)} RBF_j^{(h)}}{\hat{\Phi}_j^{(0)} + \sum_{i \in \mathcal{H}_1} \hat{\Phi}_j^{(i)} RBF_j^{(i)}} & h \in H_1, \ldots, H_4 \end{cases}$$

where $\hat{\Phi}_j^{(h)}$ denotes the maximum likelihood estimation of $\Phi_j^{(h)}$. The posterior probability of colocalisation under the condition that the gene j is an eQTL in the first cell/tissue type is given by

$$p(\text{colocalisaiton}|\text{gene } j \text{ is an eQTL in cell type 1}) = \frac{Z_j^{(4)}}{Z_j^{(1)} + Z_j^{(3)} + Z_j^{(4)}},$$

which was used for the enrichment analysis of eQTLs and the 7 differential expression categories under the condition that the gene j is an eQTL and also colocalised with the GTEx fibroblast eQTL (as the tissue type 2).

2.3 Colocalisation anlaysis with GWAS traits

We again used the Wakefield approximation [6] to compute the Bayes factor of a GWAS locus (defined by $\mathcal{W}_j$) from the estimated effect size $\hat{\beta}_l^{(\text{GWAS})}$ and its standard error $\hat{\sigma}_l^{(\text{GWAS})}$ of a variant $l \in \mathcal{W}_j$, such that

$$BF_l^{(\text{GWAS})} = \sqrt{1 - r_l} \exp\left\{ \frac{z_l^2}{2} r_l \right\},$$

where

$$z_l = \frac{\hat{\beta}_l^{(\text{GWAS})}}{\hat{\sigma}_l^{(\text{GWAS})}}$$

and

$$r_l = \frac{W}{W + \hat{\sigma}_l^2}.$$

We then compute the regional Bayes factor for the corresponding gene j as

$$RBF_j^{(2)} = \frac{1}{\#\mathcal{W}_j} \sum_{l \in \mathcal{W}_j} BF_l^{(\text{GWAS})},$$

and the joint Bayes factor as

$$RBF_j^{(12)} = \frac{1}{\#\mathcal{W}_j} \sum_{l \in \mathcal{W}_j} BF_{jl}^{(1)} BF_l^{(\text{GWAS})}.$$

Here $BF_{jl}^{(1)}$ is the eQTL Bayes factor for the first cell/tissue type and the eQTL for the second cell/tissue was essentially replaced by the GWAS locus within $\mathcal{W}_j$.

To compute the posterior probability of colocalisation $Z_j^{(4)}$, we used the fixed prior probabilities $\{\Pi_1, \Pi_2, \Psi_{12}\} = \{0.2, 0.05, 0.01\}$ to compare different GWAS traits with varying sample sizes.

References

[1] Titsias M, Lawrence ND (2010) Bayesian Gaussian process latent variable model. Thirteenth International Conference on Artificial Intelligence and Statistics .

[2] Cuomo ASE, Heinen T, Vagiaki D, Horta D, Marioni JC, et al. (2021) CellRegMap: A statistical framework for mapping context-specific regulatory variants using scRNA-seq. bioRxiv .

[3] Veyrieras JB, Kudaravalli S, Kim SY, Dermitzakis ET, Gilad Y, et al. (2008) High-resolution mapping of expression-qtls yields insight into human gene regulation. PLoS Genet 4: e1000214.

[4] Pickrell JK, Berisa T, Liu JZ, Segurel L, Tung JY, et al. (2016) Detection and interpretation of shared genetic influences on 42 human traits. Nat Genet 48: 709–717.

[5] Kumasaka N, Knights AJ, Gaffney DJ (2019) High-resolution genetic mapping of putative causal interactions between regions of open chromatin. Nat Genet 51: 128–137.

[6] Wakefield J (2010) Bayesian methods for examining hardy-weinberg equilibrium. Biometrics 66: 257-65.

3. Benchmarking the GP regression approach for eQTL mapping using synthetic data sets

3.1. Synthetic data generation

We generated synthetic data based on our GP regression model under the null or alternative hypotheses by varying the number of donors ranging from 5-1000, the number of cells ranging from 1-1000, the variance explained by a variant ranging from 0-10% and the minor allele frequency between 0.01-0.5. As with the GPLVM fitted to the fibroblast data, we stuck to 6 latent variables and one cell cycle latent variable generated from the standard normal distribution and the uniform distribution with the support [0-π], respectively. We only introduced the donor as a random effect (in Z matrix in the GP regression) with the variance parameter of 0.01.

We specifically considered the four different simulation scenarios, which are combinations of linear/nonlinear context effect and context dependent/independent donor effect according to the model specifications of GASPACHO and CellRegMap (see section 1.7 of **Supplementary Notes** for details in model comparison). We also generated synthetic data only with a static genetic effect and an additive effect of both linear and nonlinear dynamic genetic effects. A part of synthetic data was converted into discrete count data using a Poisson distribution in which simulated logCPM values were treated as log mean parameters. The generated Poisson random numbers were then converted back to log normalised expression (see section 1.8 of **Supplementary Notes** for more details). Those synthetic data were used for comparing GASPACHO to other approaches as well as power analysis and runtime analysis demonstrated in **Fig. S1** and S2. See section 1.8 of **Supplementary Notes** for more details.

3.2. Score-based test for assessing statistical calibration

We also implemented the Score-based test statistic to obtain P-values in the frequentist framework to assess statistical calibration of GASPACHO's GP regression in contrast with other approaches, such as CellRegMap. Because the score statistic can be obtained with model parameters inferred under the null hypothesis, it is straightforward to compute it utilising the maximum a posteriori parameter estimate from GPLVM. We used the similar score statistic implemented in CellRegMap (version 0.0.3; https://limix.github.io/CellRegMap/) which follows a mixture of Chi-square distributions. See Section 1.6 of **Supplementary Notes** for more details.

3.3. Sensitivity and specificity analysis of GASPACHO in comparison to CellRegMap

We performed an intensive analysis of sensitivity and specificity to map eQTLs using synthetic data under various scenarios. In order to demonstrate that we chose a sensible approach, we compared our GP regression model with (1) a pseudo-bulk

approach, (2) a reduced version of our model that only takes account of static donor effects, and (3) CellRegMap - a recently developed method for dynamic QTL mapping using single-cell genomics data, which assumes linear relationship context-specific genetic effects (**Table S1**; **Fig. S1**; Methods in the main text). We specifically considered the following four different scenarios, where two of them were generated based on linear dynamic eQTL (genotype-by-context or GxC interaction) effects, as assumed by CellRegMap, and another two with nonlinear dynamic eQTL effects, as assumed by GASPACHO. For both linear/nonlinear effects, the donor effect was assumed to be either dependent on the context or independent of the context. The results suggest that, in terms of specificity (first column of **Fig. S1**), the P-values are well calibrated (i.e., uniformly distributed) both for CellRegMap and GASPACHO, while less well calibrated for the reduced GASPACHO model when the donor effect is context-dependent (second and fourth rows of **Fig. S1**). In terms of sensitivity, GASPACHO outperforms CellRegMap in cases where the genetic effects are nonlinear (third and fourth rows of **Fig. S1**). For both approaches the sensitivity and specificity performances match the model assumptions used by the methods (i.e. CellRegMap and GASPACHO assumptions match the second and fourth scenario, respectively, and the reduced GASPACHO model matches the third scenario in **Fig. S1,** and their performances in these scenarios are shown to be the best). As expected, the linear regression for pseudo-bulk data was statistically calibrated, but there was no power to detect dynamic eQTLs. In addition, there was also no power to detect nonlinear dynamic eQTL effects using CellRegMap even with single-cell expression data (third and fourth rows of **Fig. S1**).

It is also important to emphasise that, specifically for immune responses, the dynamic genetic effect is frequently expected to be nonlinear and may also be confounded with context-dependent environmental and trans genetic effects. Thus, the most realistic scenario would be the fourth scenario in our simulations (the fourth row of **Fig. S1**), and in this case, GASPACHO outperforms CellRegMap.

3.4. Sensitivity analysis of GASPACHO's GP regression model

We further performed a power analysis of GASPACHO, by varying the numbers of donors and cells. For common genetic variants (minor allele frequency; MAF=0.2), the power to detect dynamic eQTLs increased monotonically with the total number of cells (N x n_c). It also increased when more donors were included for the same number of total cells (**Fig. S2a**). Although, interestingly, the increase in power quickly reaches a saturation when the sample size is high enough (compare N=50 and 100 in **Fig. S2b**). This tendency was similar when the same amount of variance is explained by a variant whose MAF is low (MAF=0.05) (**Fig. S2c-d**). Our analysis suggests that in our stimulated fibroblast system, with 68 donors and 22,188 cells (an average of ~300 cells per individual), the power is ~70% for an average 5% variance explained (**Fig. S2a**). This suggests that had we used 10 times more cells (~3,000 cells), we would have

increased our power to 85%.

3.5. Assessment of Normality

Our approach assumes a normal distribution of gene expression that may skew the result of mapping eQTLs, because the readout of single-cell data should typically follow an overdispersed count distribution such as a negative binomial (NB) or a zero-inflated NB distribution. Indeed, our *in vitro* fibroblast data did not follow a Gaussian distribution, even after logCPM normalisation (**Fig. S2e**). We therefore simulated discrete count data from the GPLVM using an overdispersed Poisson distribution (Section 3.1 of **Supplementary Notes**) and converted it into logCPM as if it were observed from scRNA-seq (**Fig. S2f-g**). We then confirmed that this discrete data does not inflate the type-I error of mapping eQTLs (**Fig. S2h**) even when the MAF of the tested variant is 1% and 99.8% of variants have no alternative homozygote (**Fig. S2i**). We note, however, that the power to detect eQTLs significantly declined at the same specificity, due to the additional Poisson noise and lower detection limit of gene expression (**Fig. S2j**). We also note that the detection limit will increase the false positive rate of dynamic eQTLs when there is a static (persistent) eQTL that is not detectable under some cellular states with weaker expression (**Fig. S2k**). This can be overcome by increasing the sequencing depth and the complexity of single-cell library preparation (compare two Poisson discretised data sets with different sequencing coverage in **Fig. S2k**). It is also important to note that, although there is an inflation of test statistics to detect dynamic eQTLs under the scenario of static eQTLs existing in the data, the Gaussian process mixture model implemented (Section 1.4.1 of **Supplementary Notes**) overcomes this. This is because the model directly compares the two different scenarios (static or dynamic eQTLs), and correctly classifies these eQTLs with false positive rate under 1-2% (**Fig. S2l**), even when the data is discretised by a Poisson distribution (**Fig. S2m-n**).

3.6. Assessment of Scalability

Lastly, we performed runtime analysis of eQTL mapping with various numbers of cells and donors on a Linux platform (Ubuntu 18.04.6) with AMD EPYC 7713 64-Core Processors (**Fig. S2o**). The runtime increases exponentially with the total number of cells, as expected. We also found that the number of donors would increase the baseline of computational time in a multiplicative fashion, even if the total numbers of cells are identical (**Fig. S2o**). We also note that the runtime of the GP regression for GASPACHO is ~5 times slower than CellRegMap, because GASPACHO takes account of nonlinear context-dependent donor effects (**Fig. S2p**). Here, all model parameters are inferred by the GPLVM *a priori*, and the GPLVM itself implemented in GASPACHO is applicable for rapid analysis of dozens of thousands of cells (approximately 24 hours for ~20K cells).

Figures of this Supplementary Note section

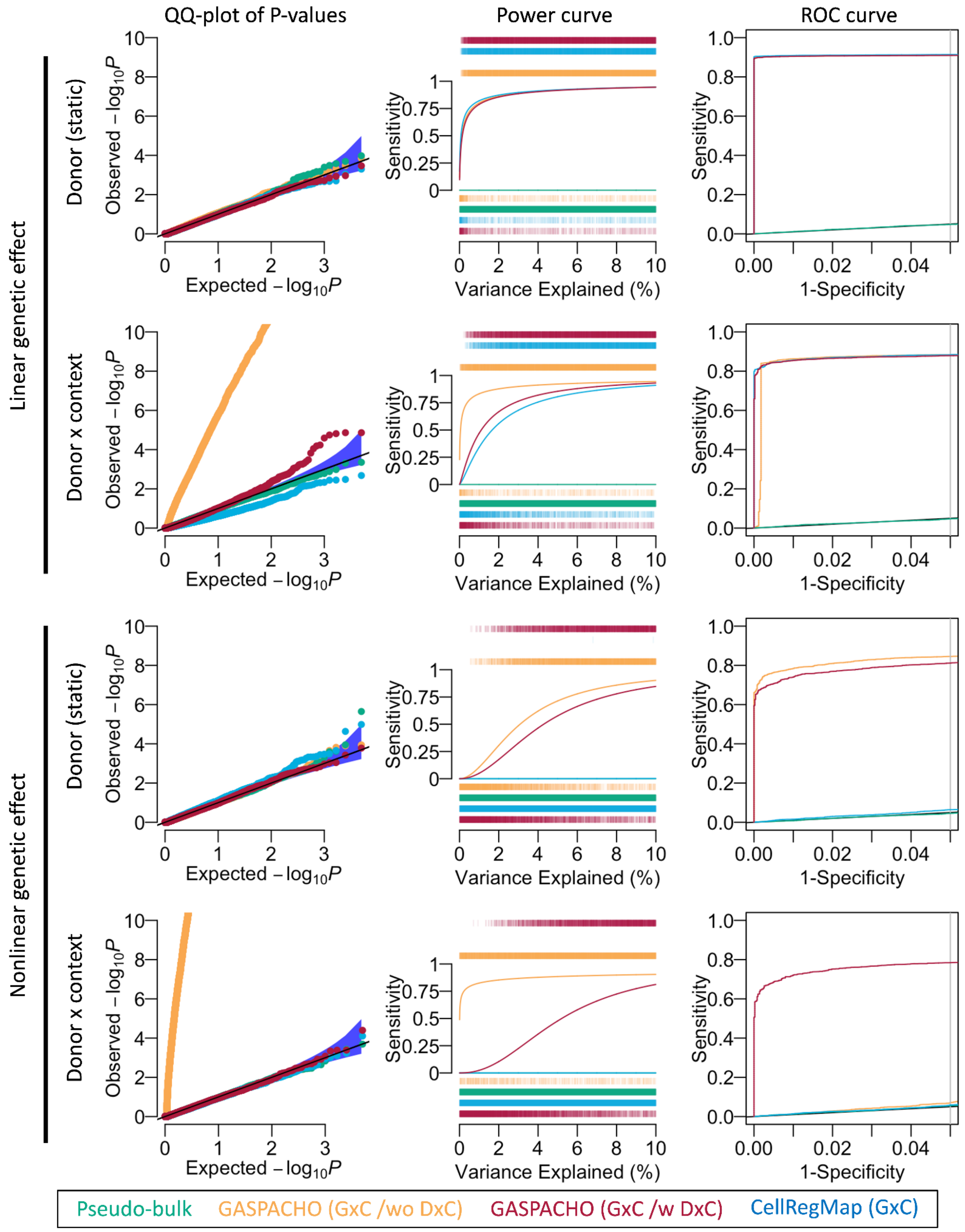

Figure S1. Comparison of sensitivity and specificity for GASPACHO models (red and orange for a dynamic eQTL effect with and without context specific donor (DxC) effect, respectively), CellRegMap (blue) and a pseudo-bulk approach (green), using synthetic data. There are four different scenarios (different rows): the top two rows

show results under linear dynamic eQTL effects (assumed by CellRegMap) and the bottom two rows show results from nonlinear dynamic eQTL effects (assumed by GASPACHO). The first and third rows show results from donor effects that are independent of the context and the second and fourth rows show results from the context-dependent donor effects (i.e., donor-context interactions. The leftmost column shows QQ-plots of *P*-values (without multiple testing correction) obtained under the null hypothesis for 5 thousand stimulated genes using the Score-based test which is boiled down to a one-sided Chi-square test (see Section 1.6 of **Supplementary Notes**). The middle column shows sensitivity (power) curves under the alternative hypothesis where the explained variance of a genetic variant (x-axis) was varied from 0 to 10% of the total variance. The power is defined at the family-wise error rate of 0.05 across 5 thousand stimulated genes. The rightmost column is ROC curves based on the same synthetic data shown in the first two columns.

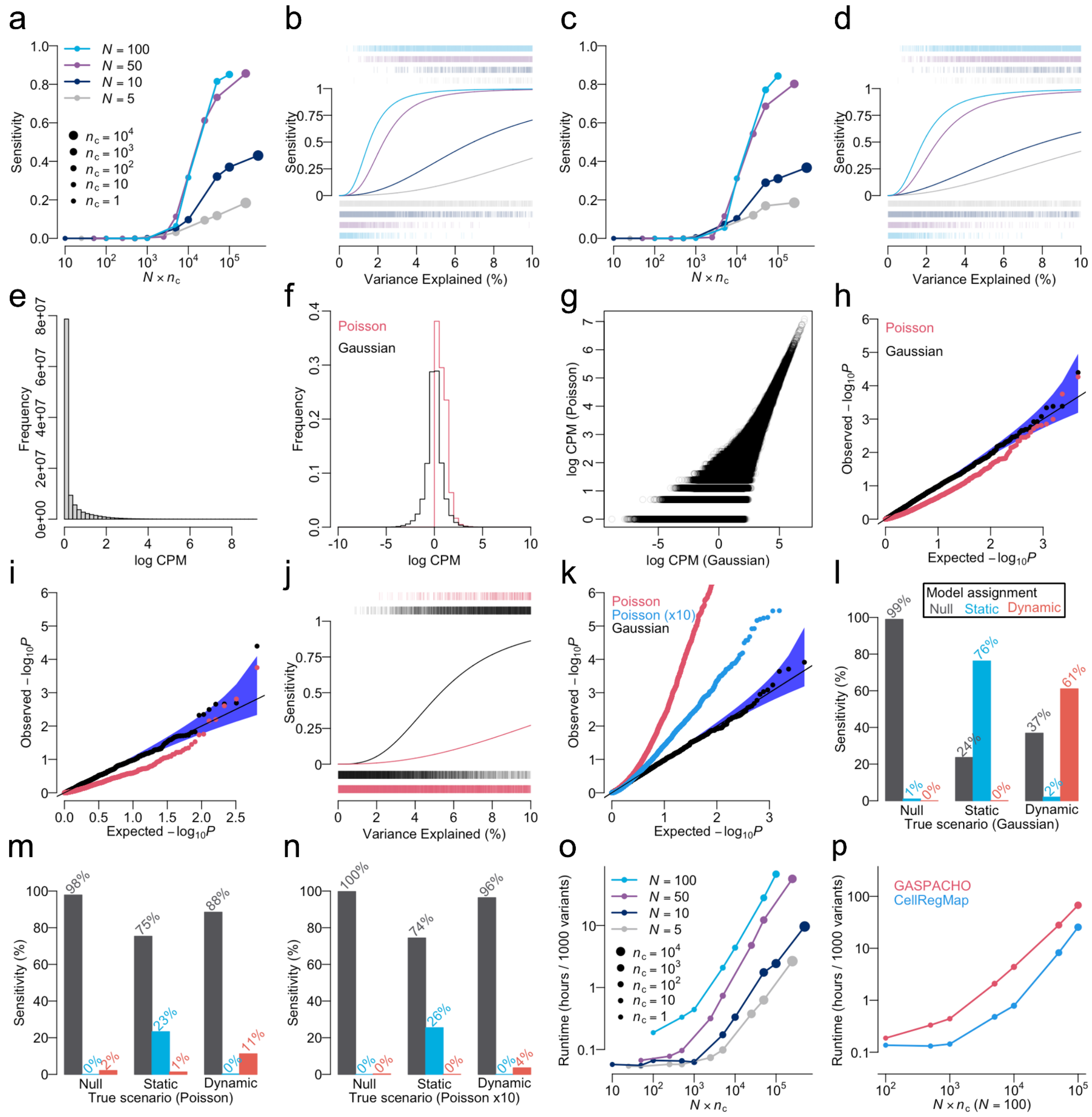

Figure S2. a. Sensitivity of GASPACHO for various numbers of donors (N) and cells (n_c) with MAF=0.2. **b**. Power curve for MAF=0.2 under the constraint $N \times n_c = 50,000$. **c.** Sensitivity of GASPACHO for various numbers of donors and cells with MAF=0.05. **d**. Power curve for MAF=0.05 under the constraint $N \times n_c = 50,000$. **e.** Distribution of logCPM of the in-vitro Fibroblast data sequenced by SmartSeq2. **f.** Distributions of simulated logCPM from the GPVLM (black) and its converted value with a Poisson distribution (red). See Section 3.1 of **Supplementary Notes** for more details for the simulation procedure. **g**. Scatterplot of logCPM values from the GPLVM and its converted values with a Poisson distribution. **h**. QQ-plot of P-values (without multiple testing correction) under the null hypothesis using the Score-based test which is boiled down to a one-sided Chi-square test (see Section 1.6 of **Supplementary Notes**). Black dots are based on simulated logCPM values and red

dots are based on the logCPM values with Poisson discretisation (see Section 3.1 of **Supplementary Notes**). **i**. QQ-plot of the same *P*-values as in panel (h) restricted with MAF=0.01 where 99.8% of variants have no alternative homozygote. The *P*-values (without multiple testing correction) were computed using the Score-based test which is boiled down to a one-sided Chi-square test (see Section 1.6 of **Supplementary Notes**). **j**. Power curve of simulated data under the alternative hypothesis. The black line indicates Gaussian data and the red line indicates Poisson converted data. **k**. QQ-plot of *P*-values to detect dynamic eQTL effects under the scenario of only static genetic effects in the data. The *P*-values (without multiple testing correction) were computed using the Score-based test which is boiled down to a one-sided Chi-square test (see Section 1.6 of **Supplementary Notes**). The black dots indicate P-values of the Gaussian expression data, the red and blue dots indicate Poisson discretised data with the expression frequency at ~60% (red) and >90% (blue) on average, where the Poisson mean is multiplied by 10 to simulate high-coverage data. **l**. Barplot showing eQTL classification results obtained from the Gaussian process mixture model (see Section 1.4.1 of **Supplementary Notes**) for the simulated logCPM values used in the panel j. The bottom categories are true scenarios under which logCPM values were generated, where "Null" indicates the null hypothesis with no eQTL effects, "Static" indicates the alternative hypothesis with only static eQTL effects and "Dynamic" indicates the alternative hypothesis with only dynamic eQTL effects. The colour codings show the model assignment (grey: Null; blue: Static; red: Dynamic). **m**. Barplot showing the eQTL classification result obtained from the Gaussian process mixture model (see Section 1.4.1 of **Supplementary Notes**) for the simulated logCPM values descritised by a Poisson distribution used in the panel j. The colour codings are the same as the panel l. **n**. Barplot showing the eQTL classification results obtained from the Gaussian process mixture model (see Section 1.4.1 of **Supplementary Notes**) for the simulated logCPM values descritised by a Poisson distribution with 10 times larger coverage than panel m. The colour codings are the same as in l. **o**. Runtimes of GASPACHO with various numbers of donors (N) and cells (n_c). **p**. Runtime comparison between GASPACHO and CellRegMap for N=100.

Table of this Supplementary Note section

	Linear regression	GASPACHO (reduced model)	GASPACHO	CellRegMap (dynamic effect)
**Model**	$\boldsymbol{y}^* = \alpha\mathbf{1} + \beta_g \boldsymbol{g}^* + \boldsymbol{\varepsilon}$<br>$\beta_g$: genetic effect (fixed parameter)<br>$\boldsymbol{\varepsilon} \sim N(0, \sigma^2 I)$: residual<br><br>$\boldsymbol{y}^*$: Pseudo-bulk gene expression vector of N_d individuals<br>$\boldsymbol{g}^*$: genotype dosage of N_d individuals	$\boldsymbol{y} = \boldsymbol{\alpha} + \boldsymbol{\beta} \odot \boldsymbol{g} + Z\boldsymbol{\gamma} + \boldsymbol{\varepsilon}$<br>$\boldsymbol{\alpha} \sim N(0, \sigma^2 K_X)$: context effect<br>$\boldsymbol{\beta} \sim N(0, \delta^2\sigma_j^2(11^T + K_X))$: genetic effect<br>$\gamma \sim N(\eta, \sigma^2\Delta)$: donor effect<br>$\varepsilon \sim N(0, \sigma^2\Omega)$: residual<br><br>$\boldsymbol{y}$: Single-cell expression vecrot of N cells<br>$\boldsymbol{g}$: genotype dosage of N cells<br>Z: design matrix of donor assignment and other confounding factors, such as sequencing plates<br>X: Latent variables<br>K_X: Nonlinear kernel matrix based on X<br>Ω: Cell specific residual variance<br>σ^2: Gene specific residual variance<br>Δ: Variance parameters for donor (and other confounding factors, such as sequencing plates).	$\boldsymbol{y} = \boldsymbol{\alpha} + \boldsymbol{\beta} \odot \boldsymbol{g} + Z\boldsymbol{\gamma} + \boldsymbol{u} + \boldsymbol{\varepsilon}$<br>$\boldsymbol{\alpha} \sim N(0, \sigma^2 K_X)$: context effect<br>$\boldsymbol{\beta} \sim N(0, \delta_{gxc}^2\sigma^2(11^T + K_X))$: genetic effect<br>$\gamma \sim N(\eta, \sigma^2\Delta)$: donor effect<br>$\boldsymbol{u} \sim N(0, \delta_{dxc}^2\sigma^2 K_X \odot R)$: donor x context effect<br>$\varepsilon \sim N(0, \sigma^2\Omega)$: residual<br><br>$\boldsymbol{y}$: Single-cell expression vecrot of N cells<br>$\boldsymbol{g}$: genotype dosage of N cells<br>Z: design matrix of donor assignment and other confounding factors, such as sequencing plates<br>X: Latent variables<br>K_X: Nonlinear kernel matrix based on X<br>Ω: Cell specific residual variance<br>σ^2: Gene specific residual variance<br>Δ: Variance parameters for donor (and other confounding factors, such as sequencing plates).	$\boldsymbol{y} = \boldsymbol{\alpha} + \beta_g\boldsymbol{g} + \boldsymbol{\beta} \odot \boldsymbol{g} + \boldsymbol{u} + \boldsymbol{\varepsilon}$<br>$\boldsymbol{\alpha} \sim N(0, \nu_1 XX^T)$: context effect<br>$\beta_g$: genetic effect (fixed parameter)<br>$\boldsymbol{\beta} \sim N(0, \nu_2 XX^T)$: genetic effect<br>$\boldsymbol{u} \sim N(0, \nu_3 XX^T \odot R)$: donor effect<br>$\boldsymbol{\varepsilon} \sim N(0, \nu_4 I_N)$: residual<br><br>$\boldsymbol{y}$: Single-cell expression vecrot of N cells<br>$\boldsymbol{g}$: genotype dosage of N cells<br>X: Latent variables<br>$\nu_1, \nu_2, \nu_3, \nu_4$: Variance parameters
**Expression data**	Pseudo-bulk	Single-cell	Single-cell	Single-cell
**Dimensional reduction**	N/A	GPLVM	GPLVM	any (requied separately before mapping eQTLs)
**Context effect**	N/A	nonlinear	nonlinear	linear
**Donor effect**	N/A (marginalised in the residula)	context independent	context dependent	context dependent (donor x context interaction)
**Dymanic genetic effect (GxC interaction)**	N/A	nonlinear	nonlinear	linear
**Model parameters**	inferred when testing genetic effects	infered by GPLVM a priori	infered by GPLVM a priori	inferred when testing genetic effects
**Hypothesis testing**	static genetic effect	static and dynamic genetic effect	static and dynamic genetic effects	static and dynamic genetic effects (tested separately)
**Test statistic**	Wald	Bayes factor/Score-based test statistic for simulation	Bayes factor/Score-based test statistic for simulation	Score-based test statistic

Table S1: Schematic comparison of a pseudo-bulk approach, a reduced GP regression model of GASPACHO for mapping static genetic effect, full GASPACHO model and CellRegMap used in **Figure. S1**.
