# Supplementary material for: Mapping interindividual dynamics of innate immune response at single-cell resolution
Source: Nat Genet. 2023 Jun 12;55(6):1066–75. doi: 10.1038/s41588-023-01421-y (PMC10260404; doi:10.1038/s41588-023-01421-y)
Supplement: Supplementary file 2 — Reporting Summary [file 41588_2023_1421_MOESM2_ESM.pdf]

nature portfolio

Corresponding author(s): Sarah Teichmann
Tzachi Hagai

Last updated by author(s): 19/4/22

Reporting Summary

Nature Portfolio wishes to improve the reproducibility of the work that we publish. This form provides structure for consistency and transparency in reporting. For further information on Nature Portfolio policies, see our Editorial Policies and the Editorial Policy Checklist.

Statistics

For all statistical analyses, confirm that the following items are present in the figure legend, table legend, main text, or Methods section.

n/a	Confirmed	
☐	☒	The exact sample size (*n*) for each experimental group/condition, given as a discrete number and unit of measurement
☐	☒	A statement on whether measurements were taken from distinct samples or whether the same sample was measured repeatedly
☐	☒	The statistical test(s) used AND whether they are one- or two-sided<br>*Only common tests should be described solely by name; describe more complex techniques in the Methods section.*
☐	☒	A description of all covariates tested
☐	☒	A description of any assumptions or corrections, such as tests of normality and adjustment for multiple comparisons
☐	☒	A full description of the statistical parameters including central tendency (e.g. means) or other basic estimates (e.g. regression coefficient) AND variation (e.g. standard deviation) or associated estimates of uncertainty (e.g. confidence intervals)
☐	☒	For null hypothesis testing, the test statistic (e.g. *F*, *t*, *r*) with confidence intervals, effect sizes, degrees of freedom and *P* value noted<br>*Give P values as exact values whenever suitable.*
☐	☒	For Bayesian analysis, information on the choice of priors and Markov chain Monte Carlo settings
☐	☒	For hierarchical and complex designs, identification of the appropriate level for tests and full reporting of outcomes
☐	☒	Estimates of effect sizes (e.g. Cohen's *d*, Pearson's *r*), indicating how they were calculated

Our web collection on statistics for biologists contains articles on many of the points above.

Software and code

Policy information about availability of computer code

Data collection	CEVIChE (App 2019): https://saezlab.shinyapps.io/ceviche/
Data analysis	R 3.6.1 (https://www.r-project.org/)<br>Beagle 4.0 (https://faculty.washington.edu/browning/beagle/b4_0.html)<br>skewer 0.1.127 (https://github.com/relipmoc/skewer)<br>STAR 2.5.3a (https://github.com/alexdobin/STAR/releases)<br>featureCounts 2.0.0 (http://subread.sourceforge.net/)<br>demuxlet 0.1.0 (https://github.com/statgen/demuxlet)<br>CrossMap 0.5.2 (https://crossmap.readthedocs.io/en/latest/)<br>cellRegMap 0.0.3 (https://limix.github.io/CellRegMap/)<br>GASPACHO 0.0.1 (https://github.com/natsuhiko/GASPACHO)

For manuscripts utilizing custom algorithms or software that are central to the research but not yet described in published literature, software must be made available to editors and reviewers. We strongly encourage code deposition in a community repository (e.g. GitHub). See the Nature Portfolio guidelines for submitting code & software for further information.

Data

Policy information about availability of data

All manuscripts must include a data availability statement. This statement should provide the following information, where applicable:

- Accession codes, unique identifiers, or web links for publicly available datasets
- A description of any restrictions on data availability
- For clinical datasets or third party data, please ensure that the statement adheres to our policy

All Smart-seq2 cram files of our fibroblast data are available from the European Nucleotide Archive (Accession ID: PRJEB20147). The genotype data of fibroblast samples is under managed access and available through the HIPSCI portal (https://www.hipsci.org/data). The lines used in this study have the identifiers: HPSI0114pf-eipl, HPSI0114pf-fikt, HPSI0114pf-joxm, HPSI0114pf-lexy, HPSI0114pf-rozh, HPSI0114pf-vabj, HPSI0114pf-vass, HPSI0114pf-zoxy, HPSI0115pf-gifk, HPSI0115pf-melw, HPSI0115pf-zihe, HPSI0214pf-feec, HPSI0214pf-heja, HPSI0214pf-pelm, HPSI0215pf-deyz, HPSI0215pf-fawm, HPSI0215pf-hipn, HPSI0215pf-oilg, HPSI0314pf-bubh, HPSI0314pf-cuhk, HPSI0314pf-qonc, HPSI0314pf-wigw, HPSI0314pf-xugn, HPSI0414pf-ceik, HPSI0414pf-gesg, HPSI0414pf-naju, HPSI0414pf-oaqd, HPSI0514pf-fiaj, HPSI0514pf-kuco, HPSI0514pf-puie, HPSI0514pf-rutc, HPSI0514pf-sohd, HPSI0514pf-vuna, HPSI0614pf-ciwj, HPSI0614pf-miaj, HPSI0614pf-oicx, HPSI0714pf-pipw, HPSI0913pf-diku, HPSI0913pf-eika, HPSI0913pf-lise, HPSI0914pf-euts, HPSI0914pf-kajh, HPSI0914pf-laey, HPSI1013pf-garx, HPSI1013pf-jogf, HPSI1013pf-pamv, HPSI1013pf-sebz, HPSI1013pf-wopl, HPSI1013pf-wuye, HPSI1014pf-qayj, HPSI1014pf-sehl, HPSI1014pf-tixi, HPSI1014pf-toss, HPSI1014pf-tuju, HPSI1014pf-vils, HPSI1113pf-bima, HPSI1113pf-dons, HPSI1113pf-eofe, HPSI1113pf-ieki, HPSI1113pf-oaaz, HPSI1113pf-qolg, HPSI1113pf-wahn, HPSI1113pf-wetu, HPSI1114pf-ualf, HPSI1213pf-hehd, HPSI1213pf-nusw, HPSI1213pf-tolg and HPSI1213pf-xuja. The genotype data of COVID-19 PBMC samples is under managed access and available from European Genome-Phenome Archive (Accession ID: XXXXXXX). The annotation of the CpG site was downloaded from the UCSC website (https://hgdownload.soe.ucsc.edu/goldenPath/hg38/database/cpgIslandExt.txt.gz). The position weight matrices (PWM) of transcription factor motifs were obtained from CIS-BP (http://cisbp.ccbr.utoronto.ca/bulk.php). The PWM used to find TATA-box in the gene promotor have the following identifiers: M1641_1.02, M2191_1.02, M4011_1.02, M4266_1.02, M6502_1.02, M1642_1.02, M4010_1.02, M4014_1.02 and M4708_1.02. OpenTargets GWAS summary statistics are available from GWAS Catalog (https://www.ebi.ac.uk/gwas/), FINNGEN (https://www.finngen.fi/en/access_results) and UK Biobank (https://www.nealelab.is/uk-biobank). COVID-19 GWAS summary statistics (release 5) are available from the COVID-19 Host Genetics Initiative (https://www.covid19hg.org/results/r5/). The Open Targets colocalisation data is obtained from the website (https://ftp.ebi.ac.uk/pub/databases/opentargets/genetics/210608/). The eQTL summary statistics of GTEx 48 tissues as well as immune cells (iPSC derived macrophages and monocytes) under different stimulation conditions were obtained from the eQTL catalogue (http://ftp.ebi.ac.uk/pub/databases/spot/eQTL/). The 1000 Genomes Project VCF data (version: shapeit2_integrated_snvindels_v2a_27022019.GRCh38.phased) is obtained from (http://hgdownload.soe.ucsc.edu/gbdb/hg38/1000Genomes/). The summary statistics of fibroblast eQTLs are available from ZENODO (https://doi.org/10.5281/zenodo.7680146).

Field-specific reporting

Please select the one below that is the best fit for your research. If you are not sure, read the appropriate sections before making your selection.

☒ Life sciences ☐ Behavioural & social sciences ☐ Ecological, evolutionary & environmental sciences

For a reference copy of the document with all sections, see nature.com/documents/nr-reporting-summary-flat.pdf

Life sciences study design

All studies must disclose on these points even when the disclosure is negative.

Sample size	The total sample size is 68 fibroblast lines. The number was determined so that the probability to observe at least one heterozygous/minor homozygous patient at a genetic variant with minor allele frequency 0.05 is greater than 0.9. With N=68, the probability was 0.9990659.
Data exclusions	We haven't excluded any sample.
Replication	We discovered 1,275 expression QTLs in the fibroblast scRNA-seq data obtained from 68 unrelated donors of HIPSCI. Of which, we replicated the OAS1 eQTL using two independent model systems: (1) the PBMC scRNA-seq data from 112 donors including 84 COVID-19 positive individuals; and, (2) the scRNA-seq data of nasal brushing samples obtained from 33 adult COVID-19 positive patients.
Randomization	Randomization is not applicable, because this is a population based eQTL mapping study. Based on the principles of mendelian inheritance, it is hypothesized that our study design is protected against typical forms of confounding factors on gene expression, because any confounding factor is unable to alter genotypes of common germline genetic variants.
Blinding	Blinding is not applicable, because this is a population based eQTL mapping study. In principle, samples were recruited blind with respect to their genotypes and any bias in the association between a genetic variant and gene expression is unexpected.

Reporting for specific materials, systems and methods

We require information from authors about some types of materials, experimental systems and methods used in many studies. Here, indicate whether each material, system or method listed is relevant to your study. If you are not sure if a list item applies to your research, read the appropriate section before selecting a response.

Materials & experimental systems

n/a	Involved in the study
☒	☐ Antibodies
☒	☐ Eukaryotic cell lines
☒	☐ Palaeontology and archaeology
☒	☐ Animals and other organisms
☒	☐ Human research participants
☒	☐ Clinical data
☒	☐ Dual use research of concern

Methods

n/a	Involved in the study
☒	☐ ChIP-seq
☒	☐ Flow cytometry
☒	☐ MRI-based neuroimaging
